# TRACER: response-protected cross-domain alignment of cancer pharmacogenomic models with patient tumors

**DOI:** 10.1093/bib/bbag593

**Published:** 2026-10-28

**Authors:** Zhenxing Wang, Qihe Wang, Guangchao Wang, Guohua Wang, Yuming Zhao

**Affiliations:** College of Computer Science and Artificial Intelligence, Northeast Forestry University, No. 26 Hexing Road, Xiangfang District, Harbin, Heilongjiang 150040, China; College of Computer Science and Artificial Intelligence, Northeast Forestry University, No. 26 Hexing Road, Xiangfang District, Harbin, Heilongjiang 150040, China; College of Computer Science and Artificial Intelligence, Northeast Forestry University, No. 26 Hexing Road, Xiangfang District, Harbin, Heilongjiang 150040, China; College of Computer Science and Artificial Intelligence, Northeast Forestry University, No. 26 Hexing Road, Xiangfang District, Harbin, Heilongjiang 150040, China; Department of Computer Science and Technology, Faculty of Computing, Harbin Institute of Technology, No. 92 West Dazhi Street, Nangang District, Harbin, Heilongjiang 150001, China; College of Computer Science and Artificial Intelligence, Northeast Forestry University, No. 26 Hexing Road, Xiangfang District, Harbin, Heilongjiang 150040, China

**Keywords:** cancer pharmacogenomics, domain adaptation, representation alignment, multitask learning, gradient conflict, pharmacological response subtyping

## Abstract

Pharmacogenomics integrates tumor molecular features with drug responses to support precision oncology, but biological domain shifts and optimization conflicts hinder transfer from screened cell lines to unlabeled patient tumors. We developed TRACER (Transfer of Response Across domains via Conflict-aware End-to-end Regulation), an end-to-end framework combining supervised drug-response prediction, biologically conditioned neighbor alignment and response-protected gradient regulation. By projecting only conflicting components of the auxiliary alignment gradient, TRACER preserves the response-optimization direction learned from experimental labels. Across ModelID-disjoint, seen-drug splits, TRACER-Full achieved a global Pearson correlation of 0.5125 *± * 0.0192 on per-drug z-standardized test pairs, compared with 0.5179 *± * 0.0193 for Response-only, a mean difference of *-0.0054*. Mean oncogenic Hallmark neighbor consistency increased by *0.1162 ± 0.0210* in alignment-excluded The Cancer Genome Atlas audit samples. At the patient level, a source-validation-defined drug partition and uniform stability analysis supported four predicted pharmacological response subtypes (PRSs) in breast invasive carcinoma (BRCA), four in uterine corpus endometrial carcinoma (UCEC) and two in sarcoma (SARC). PRS-construction-excluded drugs characterized distinct predicted mechanism-level profiles, and pan-cancer indication recovery provided a lineage-aware internal consistency assessment of the patient rankings. TRACER provides an interpretable and auditable framework for response-preserving cross-domain alignment and it generates patient pharmacogenomic hypotheses for subsequent functional validation.

## Introduction

Therapeutic response in patients with cancer is shaped jointly by tissue of origin, driver alterations, and oncogenic signaling states, giving cancer types and their molecular subtypes distinct therapeutic vulnerabilities [1, 2]. Transcriptional and epigenetic plasticity and clonal evolution further increase interpatient and intratumoral heterogeneity [3]. The mechanical properties and cellular composition of the tumor microenvironment also influence tumor heterogeneity and treatment response [4]. Immune selection and immunoediting can further reshape tumor-cell populations, producing divergent response trajectories among patients with similar histologic diagnoses [5]. Identifying potential drug sensitivities at the patient level is therefore a central goal of precision oncology [6]. Studies of genomically matched therapy and functional precision medicine suggest that integrating molecular testing with functional drug screening can improve treatment selection for some patients [7, 8]. However, patient datasets remain constrained by limited sample sizes, substantial clinical heterogeneity, incomplete treatment records, and inconsistent efficacy end points. Consequently, patient–drug response matrices spanning multiple cancer types and drugs remain scarce [9, 10]. Addressing this label gap requires patient-level drug-response models to leverage reproducible external pharmacogenomic knowledge while clearly distinguishing computational prediction, pharmacological plausibility, and clinically validated treatment benefit.

The Genomics of Drug Sensitivity in Cancer (GDSC) and the Cancer Cell Line Encyclopedia (CCLE) provide large-scale cell-line drug-response and molecular profiles [11, 12]. DepMap adds genetic dependencies and lineage context, whereas PRISM expands compound coverage [13, 14]. These resources support diverse prediction models, but reliability and cross-study comparability remain limited [15, 16]. Platform, preprocessing, and split design materially affect performance estimates [17]. Cell-line evolution and missing stromal, immune and tissue architecture further constrain transfer to tumors [4, 18]. Thus, performance in held-out cell-line test samples establishes source-domain generalization; patient-domain transfer requires separate evaluation.

Existing studies address the gap between cancer cell lines and patient tumors through drug-response prediction, representation alignment, and cross-domain transfer. Celligner constructs a comparable transcriptomic space for tumors and cancer cell lines [19], whereas CellHit combines transcriptomic preprocessing, cross-domain representation alignment, and pretrained drug-response models in a patient-analysis workflow [20]. CaDRReS [21] uses a low-dimensional drug–sample latent space for recommender-style drug-response prediction, and HiDRA [22] improves biological interpretability through hierarchical pathway representations and attention. drug Graph and gene Pathway based Drug response prediction method (GPDRP) [23] jointly models drug molecular graphs and sample pathway activity, whereas context-aware deconfounding autoencoder (CODE-AE) decomposes shared and domain-specific representations to extract cross-domain signals [24]. Precision Medicine Prediction using an Adversarial Network for Cancer Drug Response (PANCDR) uses unlabeled clinical data and adversarial learning to reduce distributional differences between preclinical and patient samples [25]. Recent work has also linked patient predictions with pathway-level interpretation and external experimental validation [26]. Although these methods establish the feasibility of cross-domain drug-response prediction, few frameworks explicitly encode the evidential priority of a labeled response task over an unlabeled alignment task during joint training, or provide an optimization rule that directly audits whether auxiliary alignment alters the drug-response discriminative direction.

Gradient conflict and scale imbalance can impair multitask learning [27, 28]. PCGrad projects conflicting gradients, whereas GradNorm balances training rates and magnitudes [27, 29]. CAGrad balances average and worst local improvement, while FAMO dynamically balances loss reduction [30, 31]. Because response regression is label-supervised and alignment uses unlabeled structure, TRACER assigns priority to the response gradient.

At the framework level, TRACER integrates supervised drug-response prediction with biologically conditioned cross-domain alignment. TRACER uses GDSC cell-line drug-response data as the supervised primary task [11] and unlabeled gene-expression and mutation profiles from The Cancer Genome Atlas (TCGA) pan-cancer samples to define target-domain constraints [32]. A shared sample encoder jointly learns drug-response discrimination and cross-domain representations, while MSigDB Hallmark gene sets characterize sample-level pathway activity [33]. The model defines biologically conditioned neighbors by cancer type, mutation-profile comparability, and Hallmark pathway activity, thereby favoring cross-domain matches between local regions with comparable biological contexts. TRACER introduces asymmetric task prioritization by preserving the supervised response gradient and projecting only the conflicting component of the auxiliary alignment gradient. We evaluate this design through source-domain drug-response prediction, Hallmark neighbor consistency, gradient-optimization diagnostics, stability-guided PRS selection, characterization on drugs excluded from partition construction, and a lineage-aware internal consistency analysis of indication recovery. The predicted patient drug-response profiles can in turn generate testable pharmacological hypotheses for functional models such as patient-derived tumor organoids [34].

## Materials and methods

### Problem definition and study design

We considered GDSC2 cancer cell lines with measured drug responses as a labeled source domain and TCGA patient tumors without systematic drug-response labels as an unlabeled target domain. Let $\mathcal D=\{d_{j}\}_{j=1}^{N_{d}}$ be the global drug set, and let $\mathcal D_{i}\subseteq \mathcal D$ contains the drugs measured in source sample *i*. The learning problem is 


(1) \begin{align*}& \begin{aligned} \mathcal S&=\{(x_{i}^{e},x_{i}^{m},d_{j},y_{ij})\mid i=1,\ldots,N_{s},\ d_{j}\in\mathcal D_{i}\},\\ \mathcal T&=\{(u_{k}^{e},u_{k}^{m})\mid k=1,\ldots,N_{t}\}, \end{aligned}\end{align*}

where $x_{i}^{e},x_{i}^{m}$ and $u_{k}^{e},u_{k}^{m}$ are expression and mutation features for source cell line *i* and target patient *k*, respectively. $y_{ij}$ is the observed LN_IC50 response to drug *j*, while $N_{s},N_{t}$, and $N_{d}$ denote the numbers of source samples, target samples, and drugs, respectively. Cell-line ModelIDs were mutually exclusive among training, validation, and test sets. TCGA samples contributed only to unsupervised alignment and patient inference. All per-drug label statistics used below were estimated exclusively from source-domain training data.

### Overall architecture

TRACER couples supervised drug-response prediction to cross-domain representation alignment within one end-to-end model. Its components are a sample encoder shared by cell lines and patients, a multimodal drug encoder, a sample–drug interaction module, a response-prediction head, and an alignment-projection head (Fig. 1). The common forward mapping is 


(2) \begin{align*}& \begin{aligned} h_{i}^{s}&=f_{\mathrm{cell}}(x_{i}^{e},x_{i}^{m}),& h_{k}^{t}&=f_{\mathrm{cell}}(u_{k}^{e},u_{k}^{m}),\\ z_{j}&=f_{\mathrm{drug}}(d_{j}),& v_{ij}^{s}&=\Phi(h_{i}^{s},z_{j}),& v_{kj}^{t}&=\Phi(h_{k}^{t},z_{j}), \end{aligned}\end{align*}

**Figure 1:**
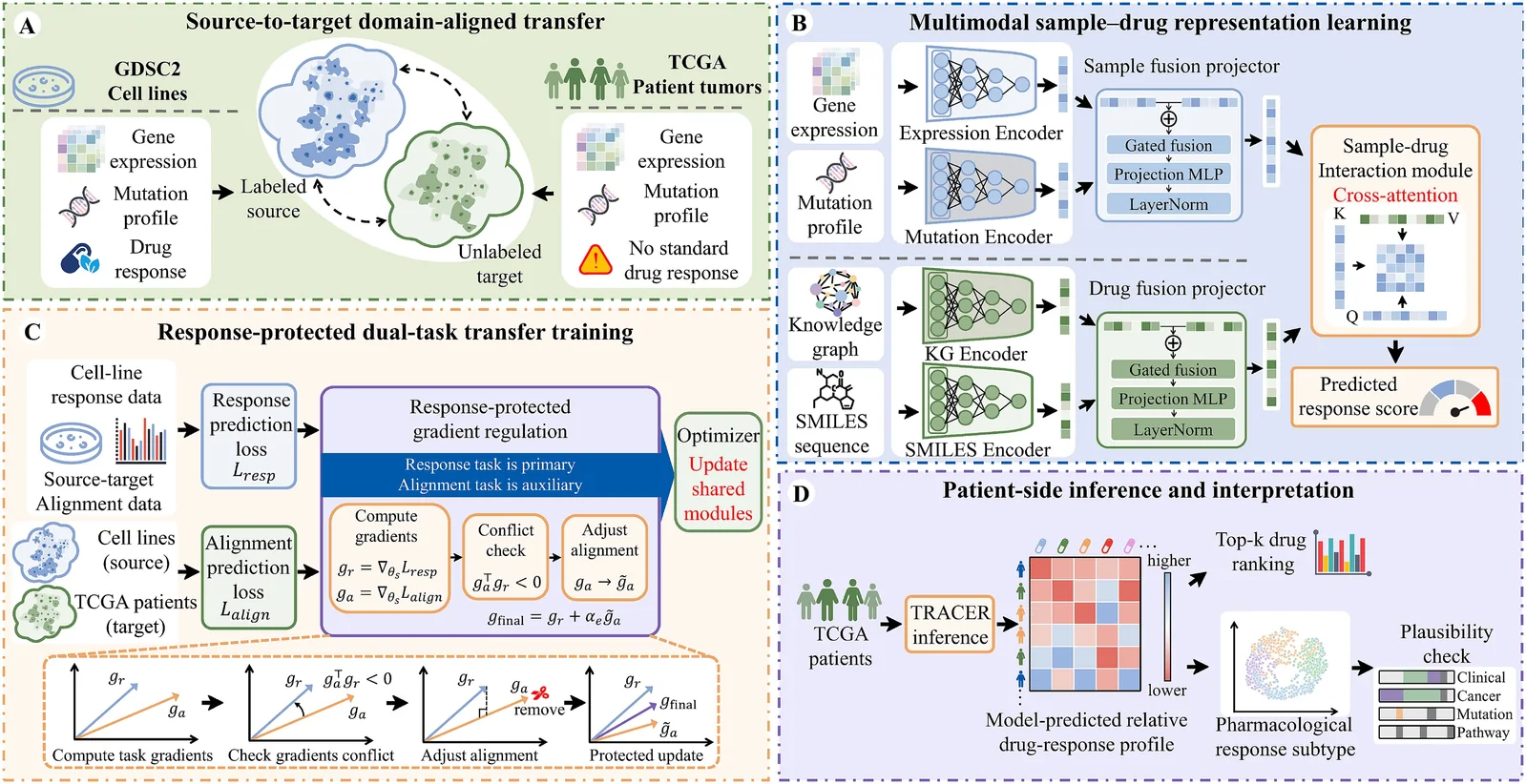
TRACER framework for response-protected cross-domain alignment of cell-line pharmacogenomic models with patient tumors. (A) Study task and cross-domain alignment setting. GDSC2 cancer cell lines provide expression, mutation, and drug-response supervision, whereas TCGA patient tumors provide unlabeled molecular features. The model learns source-domain drug-response patterns under a ModelID-disjoint setting and applies the aligned model to patient-domain molecular profiles. (B) Multimodal sample–drug representation learning. The shared sample encoder extracts expression and mutation features, and the drug encoder integrates continuous structural descriptors, SMILES sequences, and drug-target and target-pathway neighborhoods. The cross-attention module produces sample-conditioned drug features used to derive raw and standardized responses and predictive uncertainty. Source cell lines and target tumors share the expression–mutation sample encoder. Source sample–drug pairs contribute the supervised response loss, and source–target sample pairs contribute the biologically conditioned alignment loss. (C) Response-protected two-task training. The response loss defines the primary gradient and alignment defines the auxiliary gradient. During conflict, the supervised response gradient is retained and the conflicting auxiliary component is removed before the combined update. (D) Patient-level inference and interpretation. The model generates predicted patient drug-response profiles. The main patient analysis uses an uncertainty-adjusted standardized residual sensitivity score; a higher score indicates higher predicted relative sensitivity.

where $h_{i}^{s}$ and $h_{k}^{t}$ are source and target sample representations, respectively, $z_{j}$ is the drug representation, and $v_{ij}^{s}$ and $v_{kj}^{t}$ are the corresponding source and target sample–drug interaction representations, respectively. $f_{\mathrm{cell}}$, $f_{\mathrm{drug}}$, and *Φ * denote the shared sample encoder, drug encoder, and interaction module, respectively. The same response-prediction head operates on $v_{ij}^{s}$ during supervised source-domain learning and on $v_{kj}^{t}$ during patient inference. Experimental responses directly supervise the source representation and its interaction with each drug. The alignment objective acts on the same sample encoder, so the labeled response task directly constrains how patient representations move during alignment.

### Multimodal representation learning

The drug encoder combines continuous chemical descriptors, a Transformer representation of the SMILES sequence, and embeddings of drug-target and target-pathway relational tokens. Each branch is projected to a common space and fused through modality-aware gates: 


(3) \begin{align*}& z_{j}=G_{z}\!\left([m_{j}^{\mathrm{str}}z_{j}^{\mathrm{str}},m_{j}^{\mathrm{smi}}z_{j}^{\mathrm{smi}},m_{j}^{\mathrm{kg}}z_{j}^{\mathrm{kg}}]\right),\end{align*}

where $z_{j}^{\mathrm{str}},z_{j}^{\mathrm{smi}},z_{j}^{\mathrm{kg}}$ are branch representations, $m_{j}^{(\cdot )}\in \{0,1\}$ indicate modality availability, $G_{z}$ is the gated fusion function, and brackets denote concatenation. The shared sample encoder contains expression and mutation branches. Mutation features modulate the expression representation before gated fusion: 


(4) \begin{align*}\kern-.5pc e=E(x^{e}),\ m=M(x^{m}),\ (\gamma,\beta)=W_{m} m,\ h=G_{h}((1+\gamma)\odot e+\beta,m),\end{align*}

where *E* and *M* are branch encoders, $W_{m}$ produces feature-wise scale and offset vectors, $G_{h}$ is the sample-fusion gate, and *⊙ * denotes element-wise multiplication. The identical mapping is applied to source and target samples. Cross-attention subsequently conditions each drug representation on the sample state to form $v_{ij}^{s}$ or $v_{kj}^{t}$. Drug–disease, drug–indication, approval, treatment, and off-label-use relations were not used for model training or inference. PrimeKG indication relations were used only to curate the downstream indication-recovery evaluation set. Exact network dimensions and layer configurations are provided in the Supplementary Information.

### Drug-response objective

For drug *j*, let $\mu _{j}$ be its source-training response mean and define the protected standard deviation as $\sigma _{j}=\max \{\operatorname{SD}_{\mathrm{train},j},\epsilon _\sigma \}$, where $\epsilon _\sigma>0$. The standardized response is $y_{ij}^{z}=(y_{ij}-\mu _{j})/\sigma _{j}$. The response head directly predicts a standardized sample–drug residual $q_{ij}$, a bounded drug-prior correction $\delta _{j}$, and a z-scale log variance $\ell _{ij}$. The reported response means and uncertainty are deterministic: 


(5) \begin{align*}& \hat y_{ij}^{\mathrm{raw}}=\mu_{j}+\delta_{j}+\sigma_{j}q_{ij},\qquad \hat y_{ij}^{z}=\frac{\hat y_{ij}^{\mathrm{raw}}-\mu_{j}}{\sigma_{j}},\qquad \hat v_{ij}^{z}=e^{\ell_{ij}},\end{align*}

where $\hat y_{ij}^{\mathrm{raw}}$ and $\hat y_{ij}^{z}$ are raw and standardized predictions, respectively, and $\hat v_{ij}^{z}$ is predictive variance on the z scale. Raw LN_IC50 and its standardized form $y_{ij}^{z}$ share the same direction: lower values indicate greater sensitivity. The patient score $s_{kj}$ reverses this direction so that higher values indicate greater predicted relative sensitivity. The predictive standard deviation $\exp (\ell _{ij}/2)$ quantifies dispersion and has no sensitivity direction. The response objective combines eight principal terms: 


(6) \begin{align*}& \begin{aligned} L_{\mathrm{resp}}={}&\lambda_{\mathrm{raw}}L_{\mathrm{raw}} +\lambda_{z}L_{z}+\lambda_{\mathrm{res}}L_{\mathrm{res}} +\lambda_{\mathrm{Huber}}L_{\mathrm{Huber}}+\lambda_{\mathrm{NLL}}L_{\mathrm{NLL}}\\ &+\lambda_{\mathrm{center}}L_{\mathrm{center}} +\lambda_{\mathrm{drug}}L_{\mathrm{rank}}^{\mathrm{drug}} +\lambda_{\mathrm{sample}}L_{\mathrm{rank}}^{\mathrm{sample}}+L_{\mathrm{guard}}. \end{aligned}\end{align*}

$L_{\mathrm{raw}}$ and $L_{z}$ are raw- and z-scale mean-squared errors, respectively; $L_{\mathrm{res}}$ fits training-centered responses, and $L_{\mathrm{Huber}}$ provides robust regression. $L_{\mathrm{NLL}}$ models heteroscedastic z-scale uncertainty, while $L_{\mathrm{center}}$ matches drug-centered residuals. The ranking terms compare samples within drug and drugs within cell line; $L_{\mathrm{guard}}$ imposes drug-prior and residual-identifiability constraints. Exact definitions and fixed nonnegative weights are provided in the Supplementary Information.

### Biologically conditioned neighbor alignment

The alignment-projection head maps source and target representations to $a_{i}^{s}=A(h_{i}^{s})$ and $a_{k}^{t}=A(h_{k}^{t})$. Their representation similarity is 


(7) \begin{align*}& S_{ik}=\frac{\langle a_{i}^{s},a_{k}^{t}\rangle}{\|a_{i}^{s}\|_{2}\|a_{k}^{t}\|_{2}},\end{align*}

where *A* is the alignment-projection head, $\langle \cdot ,\cdot \rangle $ is the inner product, and $\|\cdot \|_{2}$ is the Euclidean norm. Three matrices encode biological comparability. Let $I_{ik}$ be the shared-mutation count, $B_{i}^{s},B_{k}^{t}$ the mutation burdens, $B_{\min }=\min (B_{i}^{s},B_{k}^{t})$, and $B_{\max }=\max (B_{i}^{s},B_{k}^{t})$. Mutation-profile comparability is 


(8) \begin{align*} & D_{ik}=\frac{2I_{ik}}{\max(B_{i}^{s}+B_{k}^{t},1)},\ Q_{ik}=\frac{I_{ik}}{\max(B_{\min},1)},\ R_{ik}=\left(\frac{B_{\min}}{\max(B_{\max},1)}\right)^\eta\kern-5pt.\end{align*}

(9) \begin{align*}& C_{ik}^{\mathrm{mut}}=\mathbf1\{I_{ik}\ge I_{\min}\} \operatorname{clip}_{[0,1]}\!\left[ \frac{w_{D}D_{ik}+w_{C}Q_{ik}}{w_{D}+w_{C}}R_{ik} \right].\end{align*}

Here, $D_{ik}$ and $Q_{ik}$ are Dice and containment overlaps, respectively,$R_{ik}$ discounts burden imbalance, and $I_{\min }$ is the shared-mutation eligibility floor. The fixed parameters satisfy $w_{D},w_{C}\geq 0$, $w_{D}+w_{C}>0$, and *η>0*; consequently, $C_{ik}^{\mathrm{mut}}=0$ whenever $I_{ik}<I_{\min }$. $C_{ik}^{\mathrm{cancer}}=\mathbf 1\{\kappa (i)=\kappa (k)\}$, where *κ * maps source lineage and target diagnosis to a common cancer-type code. For the Hallmark condition, stable L2-normalized vectors are defined by 


(10) \begin{align*}& \tilde p_{i}^{s}=\frac{p_{i}^{s}}{\max(\|p_{i}^{s}\|_{2},\epsilon_{\mathrm{path}})},\qquad \tilde p_{k}^{t}=\frac{p_{k}^{t}}{\max(\|p_{k}^{t}\|_{2},\epsilon_{\mathrm{path}})},\end{align*}

where $\epsilon _{\mathrm{path}}>0$, and $p_{i}^{s}$ and $p_{k}^{t}$ are source-training-standardized Hallmark activity vectors. Pathway comparability is $C_{ik}^{\mathrm{path}}=\max \{0,(\tilde p_{i}^{s})^\top \tilde p_{k}^{t}\}$; a zero Hallmark vector therefore contributes a condition score of zero.

For each source cell line, the operator $\mathcal N_{K}(C,\tau )$ retains up to *K* target patients with the largest condition scores satisfying $C_{ik}>\tau $. The unconditioned term uses $C^{0}=\mathbf 1$ and a fixed equal-score target subset. If no eligible pair exists, its loss is zero; otherwise, 


(11) \begin{align*}& L_{\mathrm{NN}}(C)= \frac{\sum_{(i,k)\in\mathcal N_{K}(C,\tau)}C_{ik}(1-S_{ik})} {\max\{\sum_{(i,k)\in\mathcal N_{K}(C,\tau)}C_{ik},\epsilon_{\mathrm{NN}}\}},\end{align*}

where *τ * is the eligibility threshold and $\epsilon _{\mathrm{NN}}>0$ stabilizes edge-weighted aggregation. To prevent low-dispersion embeddings, let $s_{r}^{s}$ and $s_{r}^{t}$ be population standard deviations of alignment dimension *r* in a source and target batch, respectively. The implemented dispersion penalty is 


(12) \begin{align*}& L_{\mathrm{disp}}=\frac1d\sum_{r=1}^{d}\left[\max(0,m-s_{r}^{s})+\max(0,m-s_{r}^{t})\right],\end{align*}

where *d* is alignment dimensionality and *m* is a standard-deviation floor. The full auxiliary objective is 


(13) \begin{align*} L_{\mathrm{align}}=&\ \lambda_{\mathrm{align}}\!\left[ \lambda_{0}L_{\mathrm{NN}}(C^{0})+\lambda_{\mathrm{mut}}L_{\mathrm{NN}}(C^{\mathrm{mut}}) +\lambda_{\mathrm{cancer}}L_{\mathrm{NN}}(C^{\mathrm{cancer}})\right.\nonumber\\ &\left.+\lambda_{\mathrm{path}}L_{\mathrm{NN}}(C^{\mathrm{path}}) +\lambda_{\mathrm{disp}}L_{\mathrm{disp}}\right].\end{align*}

All coefficients are fixed nonnegative weights. The dispersion regularizer is weighted by the effective coefficient $\lambda _{\mathrm{disp}}$.

### Response-protected gradient regulation

Let $\theta _{s}$ denotes shared sample-encoder parameters, $g_{r}=\nabla _{\theta _{s}}L_{\mathrm{resp}}$, and $g_{a}=\nabla _{\theta _{s}}L_{\mathrm{align}}$. TRACER identifies a conflict when $g_{a}^\top g_{r}<0$ and projects only the conflicting auxiliary component: 


(14) \begin{align*}& \tilde g_{a}= \begin{cases} g_{a}-\dfrac{g_{a}^\top g_{r}}{\|g_{r}\|_{2}^{2}+\epsilon_{\mathrm{grad}}}g_{r},&g_{a}^\top g_{r}<0,\\ g_{a},&g_{a}^\top g_{r}\geq0, \end{cases} \qquad g_{\mathrm{final}}=g_{r}+\alpha_{t}\tilde g_{a},\end{align*}

where $\epsilon _{\mathrm{grad}}>0$ is a gradient-stability constant and $\alpha _{t}$ is a non-negative auxiliary scaling coefficient. Projection is applied once to the flattened gradient of the shared sample encoder as a single operation across layers. Let $\rho _{t}$ be the scheduled auxiliary-to-response norm ratio and let $\bar \rho _{t}$ be that ratio after its configured bounds are applied. The scaling step is 


(15) \begin{align*}& \alpha_{t}=\min\!\left\{\alpha_{\max}, \frac{\bar\rho_{t}\|g_{r}\|_{2}}{\|\tilde g_{a}\|_{2}+\epsilon_{\mathrm{grad}}}\right\},\end{align*}

with zero auxiliary contribution when alignment is inactive or its gradient is degenerate. $\alpha _{\max }$ is the scale cap. $\rho _{t}$ is determined from training stage, cumulative raw-conflict rate, and late-training attenuation; its exact schedule is provided in the Supplementary Information. The response gradient is never projected. The resulting $\alpha _{t}$ scales all auxiliary gradients, while only shared sample-encoder gradients undergo projection. Thus, response-specific parameters receive response gradients, and alignment-specific parameters receive scaled but unprojected auxiliary gradients.

### Training, evaluation, and model selection

For the staged strategies, training first established the supervised response backbone and then activated alignment, using one frozen configuration across the five splits. The retained checkpoint minimized validation z-MSE, with validation Pearson used only to break ties. Response-only optimized the response objective throughout training and did not activate alignment. Simple joint activated both objectives from the beginning and applied no gradient projection. Unprotected joint used staged training and combined the two gradients without projection. Symmetric surgery used staged training and projected both gradients during conflict. TRACER-Full used staged training, preserved the response gradient and projected only the conflicting auxiliary component. Pearson correlation, Spearman correlation, and z-RMSE were computed over the same cell-line–drug keys and labels from the held-out cell-line test samples for every method. Let *b* index a formal split, let $\Omega _{b}$ be its complete set of test cell-line–drug pairs, and let $n_{b}=|\Omega _{b}|$. For pair $r\in \Omega _{b}$, $y_{r}^{z}$ and $\hat y_{r}^{z}$ denote the observed and predicted responses standardized with the corresponding per-drug source-training statistics. The global Pearson correlation is 


(16) \begin{align*}& \mathrm{Pearson}_{b}= \frac{ \sum_{r\in\Omega_{b}}(y_{r}^{z}-\bar y_{b}^{z})(\hat y_{r}^{z}-\bar{\hat y}_{b}^{z}) }{ \sqrt{ \sum_{r\in\Omega_{b}}(y_{r}^{z}-\bar y_{b}^{z})^{2} \sum_{r\in\Omega_{b}}(\hat y_{r}^{z}-\bar{\hat y}_{b}^{z})^{2} } }.\end{align*}

The global Spearman correlation is 


(17) \begin{align*}& \mathrm{Spearman}_{b}= \mathrm{Pearson}\!\left( \mathcal R(\{y_{r}^{z}\}_{r\in\Omega_{b}}), \mathcal R(\{\hat y_{r}^{z}\}_{r\in\Omega_{b}}) \right),\end{align*}

where *\mathcal R* is the rank transformation with average ranks assigned to ties. The standardized root mean squared error is 


(18) \begin{align*}& \mathrm{z}\mbox{-}\mathrm{RMSE}_{b}= \sqrt{ \frac{1}{n_{b}} \sum_{r\in\Omega_{b}}(\hat y_{r}^{z}-y_{r}^{z})^{2} }.\end{align*}

Target-domain assessment used split-specific alignment-excluded TCGA audit samples. For each patient, Hallmark and oncogenic Hallmark cosine similarities were averaged over the first five source-cell-line neighbors and then aggregated across patients and cancer types. Because Hallmark activity contributed to the alignment objective, Hallmark neighbor consistency was interpreted as an objective-aligned representation diagnostic. Gradient diagnostics used the common active joint-training window and treated the split as the statistical unit. Implementation and evaluation details for the external baselines are provided in Supplementary Table S14.

### Patient inference and pharmacological analysis

Predictions from the five TRACER-Full models were averaged. Fig. 3 The main TCGA atlas was used for transductive inference, whereas the split-specific TCGA audit samples reserved for target-domain evaluation were excluded from alignment. Patient sensitivity was represented by the uncertainty-adjusted standardized residual score 


(19) \begin{align*}& s_{kj}=-\hat y_{kj}^{z}-\lambda_{\mathrm{unc}}\exp(\ell_{kj}/2),\end{align*}

where $\hat y_{kj}^{z}$ is the standardized prediction, $\exp (\ell _{kj}/2)$ is its predictive standard deviation, and fixed $\lambda _{\mathrm{unc}}\geq 0$ controls the source-derived uncertainty penalty; higher scores indicate greater predicted relative sensitivity. Predictive-uncertainty calibration was evaluated on the five ModelID-disjoint held-out cell-line test samples using uncertainty–absolute-error Spearman correlation, Gaussian negative log-likelihood and empirical coverage of nominal 50%, 80%, and 95% intervals. For each split, a single multiplicative standard-deviation factor was estimated from the corresponding source validation set and applied unchanged to the test set. Patient-ranking robustness was evaluated for $\lambda _{\mathrm{unc}}\in \{0,0.01,0.02,0.05\}$, and PRS selection was repeated at $\lambda _{\mathrm{unc}}=0$ for BRCA, UCEC, and SARC. Eligible drugs required a mechanism assignment, at least 20 source-domain validation pairs in every formal split, a median source-validation per-drug Pearson correlation of at least 0.30 across the five splits, and finite patient-profile variability and cross-split consistency, yielding 160 drugs. For drug *j*, the source-validation quality score was 


(20) \begin{align*}& q_{j}=0.9R_{\mathrm{Pearson}}(r_{j}^{\mathrm{val}})+0.1R_{n}(n_{j}^{\mathrm{val}}),\end{align*}

where $r_{j}^{\mathrm{val}}$ is the median source-validation per-drug Pearson correlation and $n_{j}^{\mathrm{val}}$ is the minimum number of source-validation pairs across the five formal splits. Source-validation quality and target-, scaffold-, and fingerprint-based grouping produced 120 PRS-construction drugs and 40 PRS-construction-excluded drugs, with eight excluded drugs per mechanism module. Drug eligibility, quality ranking and set construction used source training and validation information exclusively. Source test sets were reserved for response-model evaluation. All 160 drugs had appeared during source-domain response-model training. Exact grouping and constrained selection are provided in the Supplementary Information.

Within each cancer type, patient-centered PRS-construction-drug profiles were clustered by KMeans for $k\in \{2,\ldots ,6\}$, using the same criteria across cancer types. Candidates required at least 20 patients and 10% of the cohort per cluster. The study-defined ARI threshold was 0.80, corresponding to good partition recovery [35]. PAC was required to be no >0.20 to limit consensus ambiguity under resampling-based validation principles [36–38]. These criteria were applied uniformly to all 33 cancer types. Sensitivity to alternative ARI and PAC cutoffs was evaluated in the Supplementary Information. Joint stability combined prediction strength, resampling ARI, inverse PAC, silhouette surplus, and cross-view ARI; its minimum null-standardized component defined the joint statistic. Empirical probabilities from 1000 matched Gaussian-copula unimodal null datasets were BH-adjusted across the five candidate resolutions [39], with FDR below 0.10 required. The largest passing *k* was retained. Without a passing candidate, no discrete PRS was assigned; patient variation was summarized by the first principal-component score as a continuous pharmacological response axis. Passing groups were termed model-derived predicted pharmacological response subtypes (PRSs). Exact resampling, view construction, null calibration, and continuous-axis procedures are provided in the Supplementary Information.

After PRS labels were frozen, their associations with cancer-specific reference classifications were summarized using Cramér’s *V*, adjusted Rand index, normalized mutual information and 10 000 label permutations. The reference classifications comprised PAM50 for BRCA, TCGA molecular classes for UCEC and TCGA histological types for SARC [40]. Across the five modules, each PRS was compared with the remaining patients using the 40 PRS-construction-excluded drugs and 2000 patient bootstraps. These comparisons characterized model-predicted pharmacological differences. Indication recovery used all 31 curated mappings independently of the drug split and served as a lineage-aware internal consistency assessment of cancer-type structure retained in the patient rankings.

## Results

### TRACER maintains robust drug-response prediction in held-out cell-line test samples

Across five ModelID-disjoint, seen-drug splits, we compared TRACER-Full with CaDRReS, CODE-AE, GPDRP, HiDRA, and PANCDR using identical partitions, test keys, and labels. As shown in Fig. 2A, TRACER-Full achieved Pearson *0.5125 ± 0.0192*, Spearman *0.4976 ± 0.0206*, and z-RMSE *0.8620 ± 0.0177*. TRACER-Full had the highest mean Pearson and Spearman correlations among the external baselines, while its z-RMSE was among the lowest. These values indicate preserved linear and rank agreement with <1 standardized unit of root-mean-square error. Split-level results are provided in the Supplementary Information.

**Figure 2:**
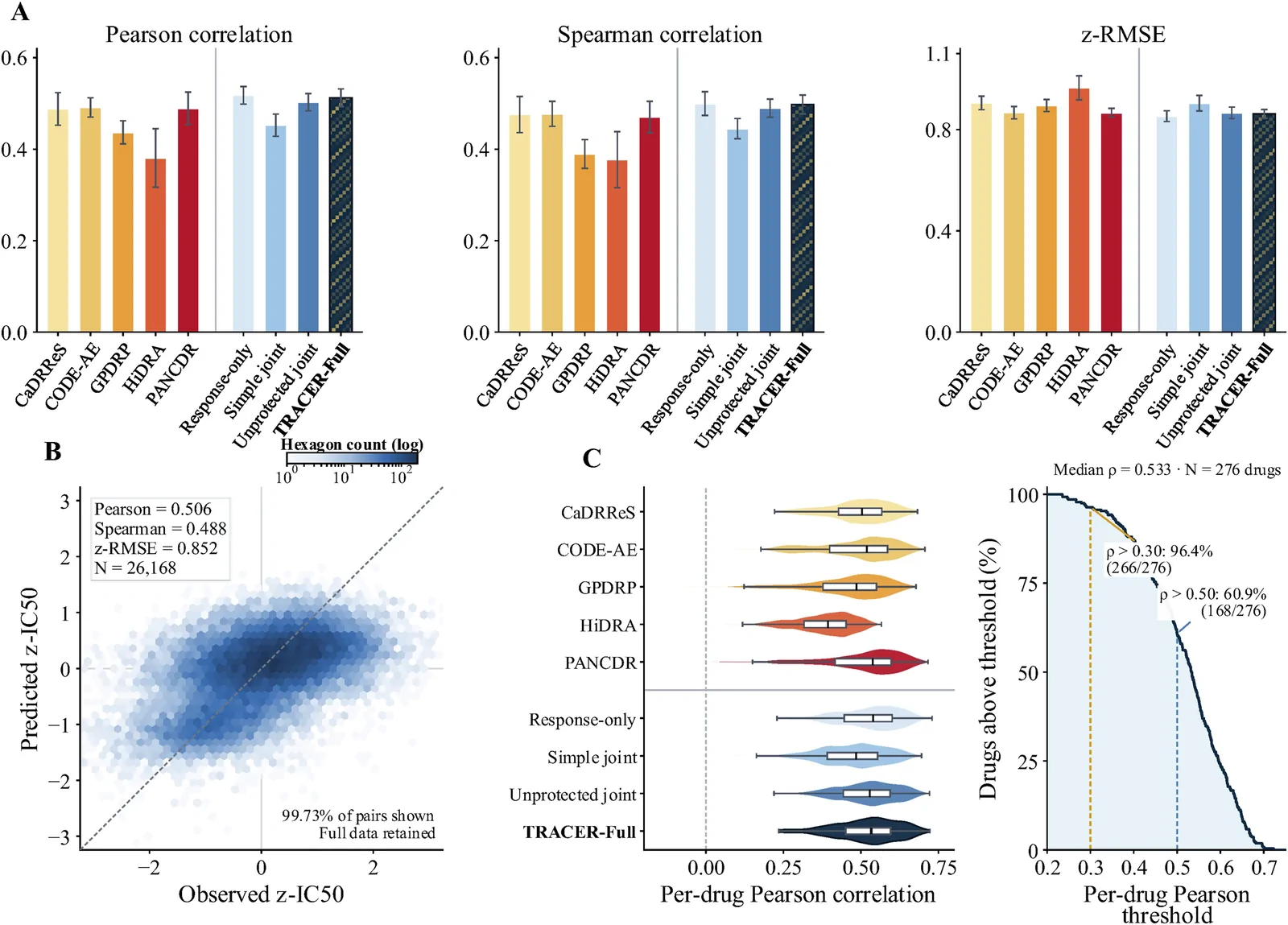
Performance comparison of TRACER and drug-response prediction baselines in ModelID-disjoint held-out cell-line test samples. (A) Overall drug-response prediction performance. CaDRReS, CODE-AE, GPDRP, HiDRA, PANCDR, and Response-only, Simple joint, Unprotected joint, and TRACER-Full were evaluated uniformly on five ModelID-disjoint, seen-drug data splits. Bars show means over the five data splits, and error bars show standard deviations. Pearson and Spearman measure linear agreement and rank agreement between predicted and observed responses; higher values indicate better performance. z-RMSE measures prediction error for standardized responses; lower values indicate better performance. Hatched bars denote TRACER-Full. (B) Observed–predicted agreement in a representative data split. The displayed split had the median TRACER-Full Pearson correlation among the five formal splits. The held-out cell-line test samples comprised 26 168 cell-line–drug pairs, and TRACER-Full achieved Pearson 0.506, Spearman 0.488, and z-RMSE 0.852. Hexagon color intensity indicates the number of cell-line–drug pairs in the corresponding region on a log10 scale, and the dashed line is the reference line for perfect agreement between observed and predicted values. The plot displays 99.73% of the cell-line–drug pairs from the held-out cell-line test samples, and all performance metrics were calculated on the complete test set. Lower z-IC50 indicates relatively higher drug sensitivity. (C) Per-drug prediction performance and coverage. The left panel shows the distributions of per-drug Pearson correlations for nine methods across 276 shared drugs, with embedded boxes indicating the median and interquartile range. The right panel shows the proportion of drugs for which the per-drug Pearson correlation of TRACER-Full exceeds different thresholds. The median per-drug Pearson correlation of TRACER-Full was 0.5334; 266 drugs were above 0.30, accounting for 96.4%, and 168 drugs were above 0.50, accounting for 60.9%. All methods used the same data splits, cell-line–drug pairs from the held-out cell-line test samples, and response labels.

**Figure 3:**
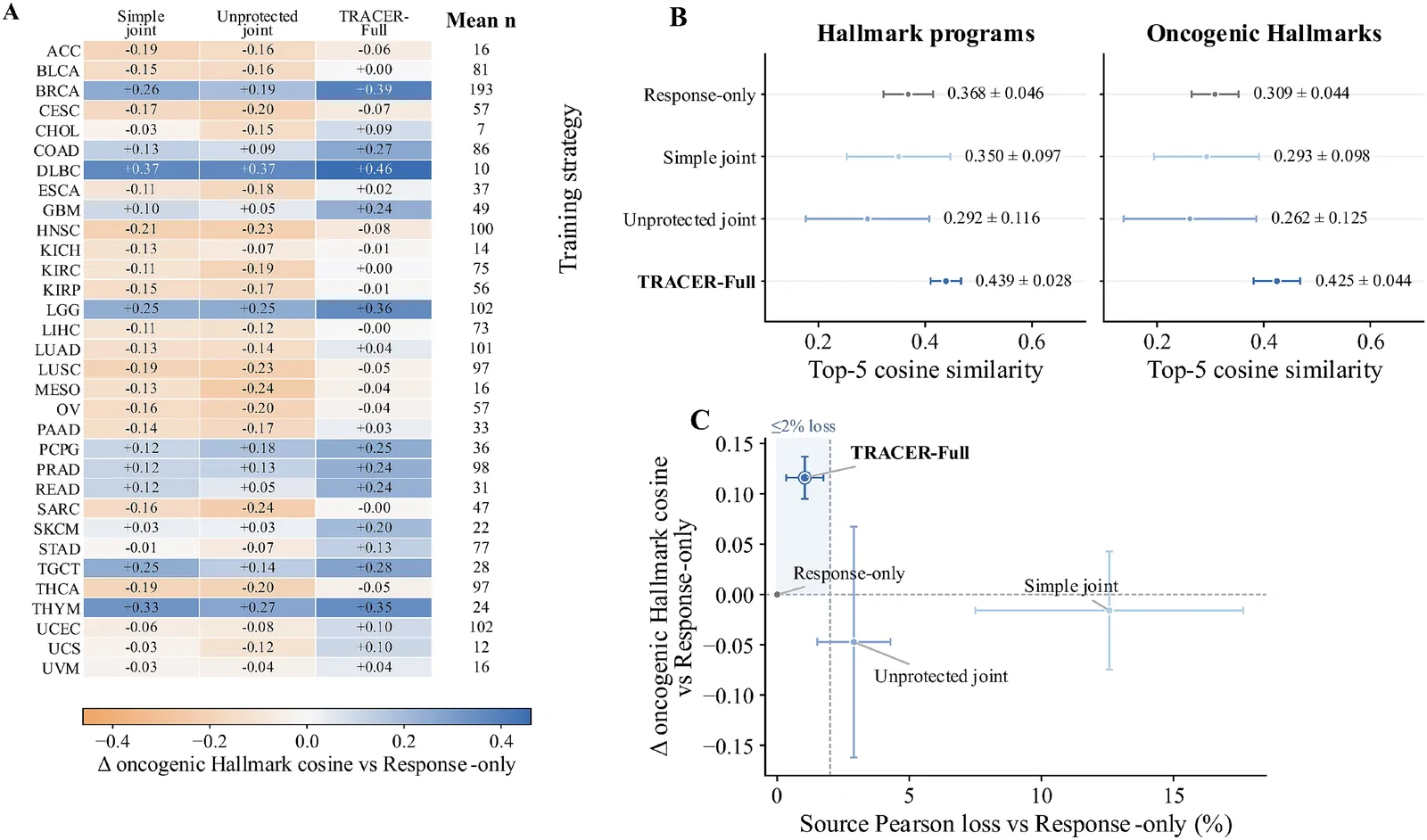
Ablation analysis of TRACER training strategies. (A) Changes in oncogenic Hallmark neighbor consistency across cancer types. The heat map shows the differences in Top-5 neighbor pathway cosine similarity for Simple joint, Unprotected joint, and TRACER-Full relative to Response-only. Positive values indicate increases in neighbor consistency, whereas negative values indicate decreases. Mean n indicates the mean number of included alignment-excluded TCGA audit samples for each cancer type. (B) Hallmark and oncogenic Hallmark top-5 neighbor consistency for four TRACER training strategies in alignment-excluded TCGA audit samples. Points indicate means across the five formal splits, and error bars indicate standard deviations. (C) Trade-off between the gain in oncogenic Hallmark neighbor consistency and the source-domain Pearson loss relative to Response-only. The x-axis is the Pearson loss relative to Response-only, and the y-axis is the increase in oncogenic Hallmark neighbor consistency. The highlighted region indicates combinations with a Pearson loss no greater than 2% and a positive improvement in pathway consistency.

Response-only served as the source-task reference because it optimized supervised drug-response prediction alone. After introducing the auxiliary cross-domain alignment objective, TRACER-Full achieved Pearson *0.5125 ± 0.0192*, compared with *0.5179 ± 0.0193* for Response-only. The mean difference was *-0.0054*, indicating that cross-domain alignment incurred only a limited reduction in source-domain correlation. As shown in Fig. 2B, the representative split contained 26 168 test pairs and yielded Pearson 0.506, Spearman 0.488, and z-RMSE 0.852. Metrics were calculated on the complete test set.

We additionally evaluated within-drug discrimination independently of between-drug response offsets. As shown in Fig. 2C, across 276 shared drugs, the median per-drug Pearson correlation of TRACER-Full was 0.5334; 266 drugs (96.4%) exceeded 0.30 and 168 drugs (60.9%) exceeded 0.50. This broad drug-level coverage indicates that the pooled performance was not driven by a small subset of compounds. Together, the pooled and per-drug results show that TRACER-Full maintained competitive source-domain drug-response prediction performance and positive within-drug response discrimination for most evaluated drugs.

### Objective-aligned hallmark neighbor consistency shows cancer-type heterogeneity

We compared four TRACER training strategies in alignment-excluded TCGA audit samples. As shown in Fig. 3B, TRACER-Full achieved Hallmark and oncogenic Hallmark neighbor consistencies of *0.439 ± 0.028* and *0.425 ± 0.044*, respectively. Response-only achieved *0.368 ± 0.046* and *0.309 ± 0.044*, respectively. The corresponding gains were 0.071 and 0.116, giving TRACER-Full the highest mean for both metrics. These measures are objective-aligned representation diagnostics because Hallmark activity contributed to alignment.

As shown in Fig. 3A, TRACER-Full exceeded Response-only in 21 evaluable cancer types. Detailed cancer-specific gains in oncogenic Hallmark neighbor consistency are provided in the Supplementary Information.

When the gain in oncogenic Hallmark neighbor consistency and the Pearson loss relative to Response-only were considered jointly, TRACER-Full provided the best observed balance among the evaluated TRACER training strategies. As shown in Fig. 3C, it incurred a Pearson loss of $1.05\% \pm 0.71\%$ while increasing oncogenic Hallmark neighbor consistency by *0.1162 ± 0.0210*. The corresponding Pearson losses were 12.57% *± * 5.07% for Simple joint and 2.90% *± * 1.39% for Unprotected joint. These comparisons show that cross-domain alignment benefits depended on how the auxiliary objective was integrated with the labeled response task. TRACER-Full improved mean Hallmark neighbor consistency between patients and cell lines while retaining nearly all of the Pearson performance of Response-only. The cancer-specific results define the observed heterogeneity around this mean improvement.

### Response-protected gradient regulation mitigates response–alignment conflicts

To determine whether response-protected gradient regulation operated as intended, we compared gradient relationships for Simple joint, Unprotected joint, Symmetric surgery, and TRACER-Full during joint training. The analysis included valid two-task batches in which both objectives produced finite gradients and used each formal split as the split-level summary unit. A negative raw gradient cosine indicates conflicting update directions in the shared sample encoder.

As shown in Fig. 4A, raw gradient conflicts were frequent for all four strategies. The rates were $72.2\% \pm 1.0\%$ for Simple joint, $70.5\% \pm 3.5\%$ for Unprotected joint, $74.4\% \pm 4.5\%$ for Symmetric surgery, and $62.0\% \pm 1.5\%$ for TRACER-Full. The persistence of negative gradient relationships across the active joint-training window indicates sustained competition between drug-response prediction and cross-domain representation alignment.

**Figure 4:**
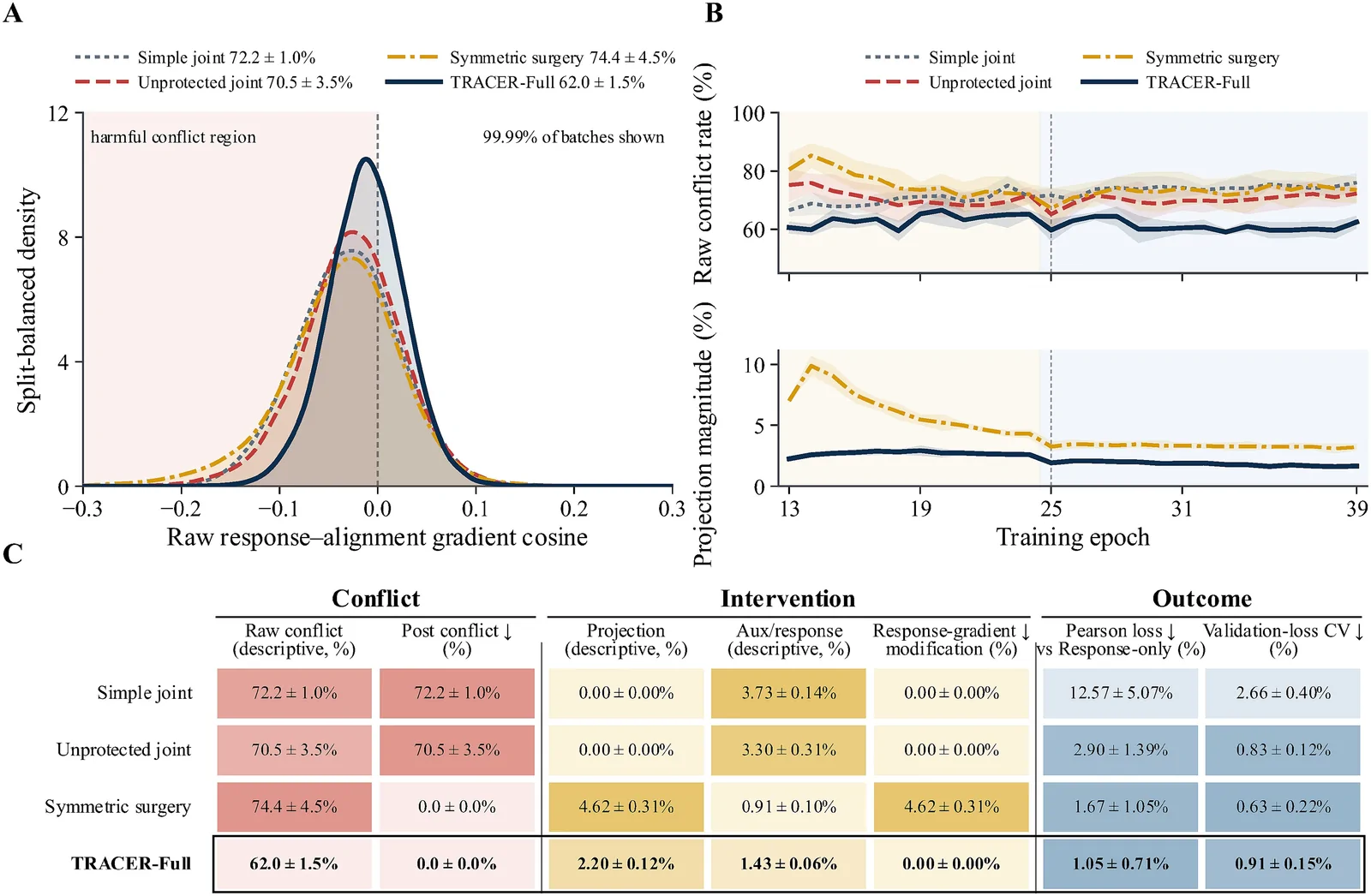
Optimization diagnostics of TRACER response-protected gradient regulation. (A) Distribution of raw gradient conflicts during joint training. Curves show data-split-balanced densities for four strategies across five data splits; the negative region indicates gradient conflict, and the legend gives the raw conflict rate. The x-axis covers 99.99% of valid two-task batches. (B) Training dynamics of gradient conflict and projection magnitude. The upper panel shows the raw conflict rate during epochs 13–39, and the lower panel shows the auxiliary-gradient projection magnitudes of Symmetric surgery and TRACER-Full. Solid lines indicate means, shaded areas indicate standard deviations, and the dashed line marks the stage transition at epoch 25. (C) Integrated comparison of conflict status, gradient intervention, and training outcome. Meaningful post-projection conflict uses a numerical tolerance of $-10^{-7}$. Projection is the proportion of the auxiliary gradient component removed, Aux/response is the norm ratio between the auxiliary and response gradients, and Response-gradient modification is the proportion of the primary response gradient modified. Pearson loss is the source-domain Pearson loss relative to Response-only, and Validation-loss CV is the coefficient of variation of validation z-MSE during epochs 26–39.

Because Simple joint and Unprotected joint did not apply auxiliary-gradient projection, their meaningful post-projection conflict rates equaled their raw conflict rates. Symmetric surgery and TRACER-Full both eliminated numerically meaningful post-projection conflicts under the $-10^{-7}$ numerical tolerance used for this diagnostic. Symmetric surgery modified both task gradients, with auxiliary-gradient projection and response-gradient modification magnitudes of $4.62\% \pm 0.31\%$. In contrast, TRACER-Full removed $2.20\% \pm 0.12\%$ of the auxiliary alignment gradient on average while maintaining zero response-gradient modification. The small mean projection magnitude indicates that only a limited auxiliary-gradient component was removed, while the optimization direction defined by experimental response labels remained unchanged.

Training dynamics supported this distinction. As shown in Fig. 4B, after alignment became active at epoch 13, all four strategies showed persistently high raw conflict rates; from epoch 25 onward, the conflict rate and projection magnitude of TRACER-Full remained comparatively stable within the analyzed window. Symmetric surgery maintained a larger projection magnitude, consistent with its broader intervention on both task gradients.

As shown in Fig. 4C, the Pearson losses relative to Response-only were $12.57\% \pm 5.07\%$, $2.90\% \pm 1.39\%$, $1.67\% \pm 1.05\%$, and $1.05\% \pm 0.71\%$ for Simple joint, Unprotected joint, Symmetric surgery, and TRACER-Full, respectively. Symmetric surgery achieved the lowest validation-loss coefficient of variation, whereas TRACER-Full had the smallest observed Pearson loss. The principal observed distinction of TRACER-Full was therefore the elimination of numerically meaningful post-projection conflicts without modifying the response gradient. These results connect response-protected gradient regulation to the observed preservation of source-domain drug-response prediction performance. Split-level gradient diagnostics and training-outcome measurements are provided in the Supplementary Information.

Across 131 114 observations from the held-out cell-line test samples, the raw predicted standard deviation had a Spearman correlation of 0.0506 with absolute error. Empirical coverage of nominal 50%, 80%, and 95% intervals was 35.7%, 61.2%, and 80.0%. Validation-set scalar calibration increased the corresponding test coverage to 54.5%, 82.8%, and 95.1% and reduced Gaussian negative log-likelihood from 1.606 to 1.286.

Patient rankings remained highly concordant after removal of the uncertainty penalty, with a median Spearman correlation of ${>}0.9999$ relative to $\lambda _{\mathrm{unc}}=0.02$. At the primary resolutions, partition agreement between $\lambda _{\mathrm{unc}}=0.02$ and zero yielded adjusted Rand indices of 0.955 for BRCA, 0.983 for UCEC, and 1.000 for SARC. The maximum supported resolution changed from *k=4* to *k=5* in BRCA, while UCEC retained *k=4* and SARC retained *k=2*. Patient rankings were stable, with BRCA resolution selection showing sensitivity at the adjacent *k=5* boundary. The stability of patient rankings across the evaluated range supports retaining $\lambda _{\mathrm{unc}}=0.02$ as a small ranking regularizer. Detailed calibration and penalty-sensitivity results are provided in Supplementary Fig. S31 and Table S12.

### Stability-guided identification of within-cancer pharmacological response subtype structures

The same stability-guided selection rule was applied to patient-centered response profiles across the 120 PRS-construction drugs. As shown in Fig. 5A, the largest candidate resolution satisfying all criteria was *k=4* for BRCA, *k=4* for UCEC, and *k=2* for SARC. The smallest groups contained 197, 108, and 105 patients, representing 20.4%, 21.2%, and 45.3% of the corresponding cohorts.

**Figure 5:**
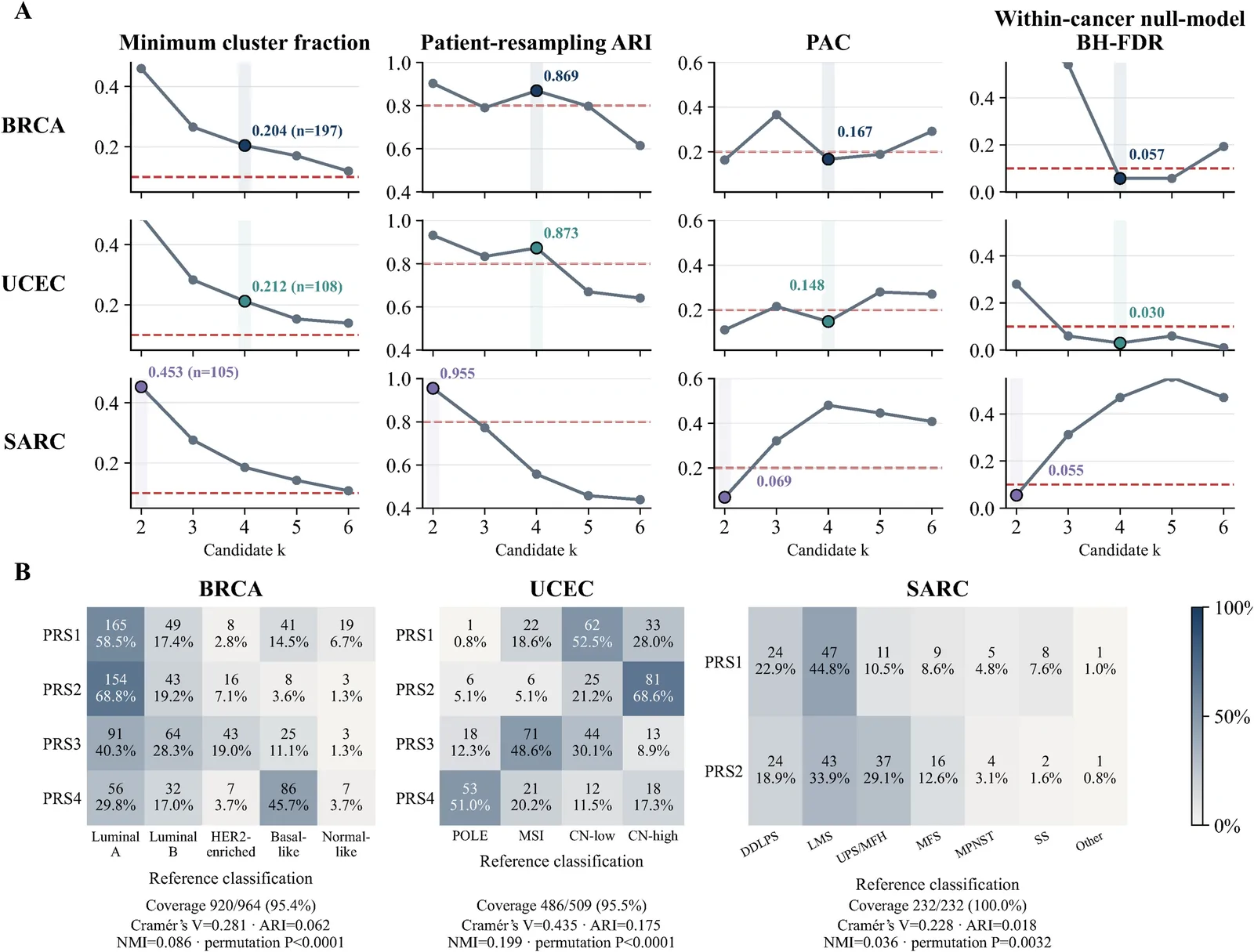
Within-cancer selection of PRS resolution and comparison with reference classifications. (A) Stability evidence across candidate *k* values in BRCA, UCEC, and SARC. Columns show minimum cluster fraction, patient-resampling adjusted Rand index, proportion of ambiguous clustering, and within-cancer null-model BH-FDR. Points represent *k=2*–6, vertical bands mark the selected resolution, and dashed lines indicate the study-defined selection thresholds. The minimum-cluster criterion also required at least 20 patients per group. (B) Association of the selected PRSs with BRCA PAM50 classes [41], TCGA UCEC molecular classes [42] and TCGA SARC histological types [40]. Cells show patient counts and percentages within each PRS. Sample-level BRCA and UCEC labels were obtained from the curated TCGA subtype resource distributed through TCGAbiolinks [43]; SARC histological types were obtained from GDC clinical records. Coverage, Cramér’s *V*, adjusted Rand index, normalized mutual information, and 10 000-permutation *P* values summarize the association between the two partitions. PRS numbers are defined only within the same cancer type.

Patient-resampling ARIs were 0.869 for BRCA, 0.873 for UCEC, and 0.955 for SARC. The corresponding PAC values were 0.167, 0.148, and 0.069, and the within-cancer null-model BH-FDR values were 0.057, 0.030, and 0.055. All three solutions satisfied the common group-size, resampling, consensus, and null-model criteria. The same resolutions were retained when the ARI threshold was increased from 0.80 to 0.85 with PAC fixed at 0.20. Together, these criteria supported four model-derived PRSs in BRCA, four in UCEC, and two in SARC. Complete results for all 33 cancer types are provided in the Supplementary Information.

We next examined how the drug-response-derived partitions related to established reference classifications. As shown in Fig. 5B, reference labels covered 95.4% of BRCA, 95.5% of UCEC, and 100.0% of SARC patients. Cramér’s *V* values were 0.281, 0.435, and 0.228, respectively. The corresponding adjusted Rand indices were 0.062, 0.175, and 0.018, and normalized mutual information values were 0.086, 0.199, and 0.036. Permutation tests indicated non-random associations for BRCA and UCEC (*P<.0001*) and SARC (*P=.0032*). The low partition-agreement measures indicate that the PRSs captured patient-response organizations distinct from the reference classifications.

### Pharmacological response subtype-construction-excluded drugs characterize predicted response profiles across pharmacological response subtypes

We next characterized the selected partitions using drugs excluded from PRS construction. As shown in Fig. 6A, the 40 PRS-construction-excluded drugs produced structured predicted response patterns across the selected PRSs. BRCA comprised groups of 295, 230, 242, and 197 patients; UCEC comprised groups of 127, 125, 149, and 108 patients; and SARC comprised groups of 105 and 127 patients. The organized response variation characterized the selected partitions after construction in the 120-drug space.

**Figure 6:**
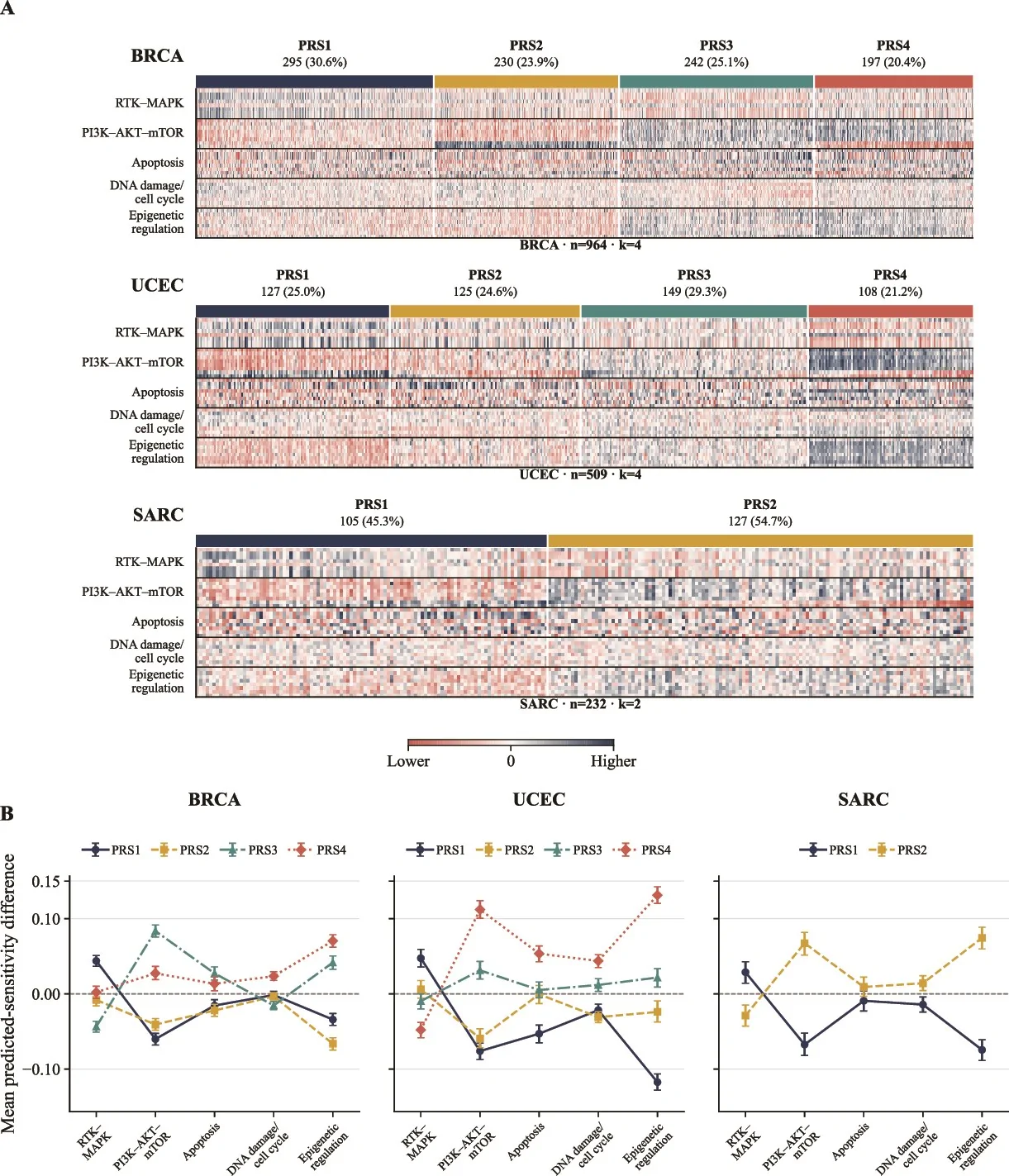
Predicted pharmacological patterns of PRSs on PRS-construction-excluded drugs. (A) Within-cancer predicted response heat maps for 40 PRS-construction-excluded drugs in BRCA, UCEC, and SARC. Each column represents a patient, and each row a PRS-construction-excluded drug; the drugs are distributed equally across five mechanism modules. Patients are grouped by PRS and ordered within each PRS by a fixed projection derived from the PRS-construction-drug space. Values above the patient-specific mean indicate higher predicted relative sensitivity, whereas values below the mean indicate lower predicted relative sensitivity. The top bands show PRS membership and patient counts. (B) Module-level predicted relative sensitivity of each PRS. Points show the mean predicted-sensitivity difference between each PRS and the remaining patients of the same cancer type across eight PRS-construction-excluded drugs per module. Error bars show 95% confidence intervals from 2000 patient bootstraps. The displayed drugs were reserved from clustering, selection of *k*, and within-PRS patient ordering.

Mechanism-level summaries further resolved the pharmacological characteristics of individual PRSs. As shown in Fig. 6B, the largest positive and negative differences in BRCA were observed for PRS3 with PI3K–AKT–mTOR drugs (0.084, 95% CI 0.076–0.092) and PRS2 with epigenetic-regulation drugs (*-0.066*, 95% CI *-0.075* to *-0.058*). In UCEC, epigenetic-regulation differences were 0.131 (95% CI 0.120–0.143) for PRS4 and *-0.117* (95% CI *-0.128* to *-0.106*) for PRS1. In SARC, the corresponding epigenetic-regulation differences were 0.075 (95% CI 0.060–0.089) for PRS2 and *-0.075* (95% CI *-0.089* to *-0.061*) for PRS1. Complete module estimates are provided in the Supplementary Information.

The PRS-construction-excluded drugs characterized mechanism-level response patterns after the partitions had been selected. These drugs were observed during source-domain model training, so the analysis describes post-selection structure within the trained drug space.

### Drug-set separation and lineage-aware internal consistency

The post-selection characterization above used drug sets separated without patient-side clustering information. As shown in Fig. 7A, source-validation information was used to divide 160 eligible drugs into 120 PRS-construction drugs and 40 PRS-construction-excluded drugs. The construction/excluded counts were 34/8 for RTK–MAPK, 12/8 for PI3K–AKT–mTOR, 8/8 for apoptosis, 44/8 for DNA damage/cell cycle, and 22/8 for epigenetic regulation. Assigning eight excluded drugs to each mechanism module enabled balanced comparison of predicted mechanism-level responses.

**Figure 7:**
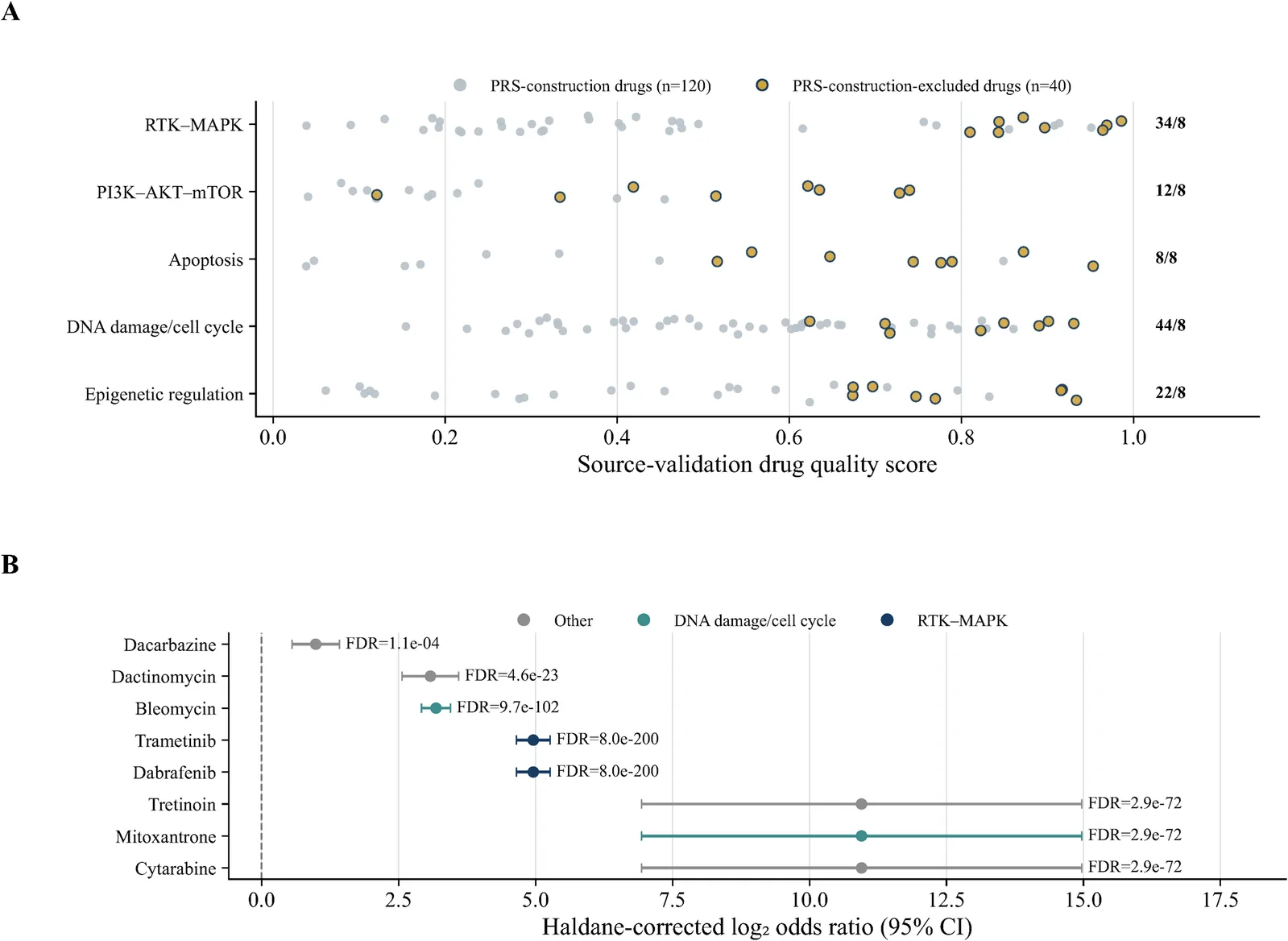
Drug-set separation and lineage-aware internal consistency of known drug–cancer indications. (A) Source-validation drug quality scores for the 120 PRS-construction and 40 PRS-construction-excluded drugs. Rows represent the five mechanism modules; gray circles denote PRS-construction drugs, and orange circles with dark outlines denote PRS-construction-excluded drugs. Numbers at right give construction/excluded counts for each module. (B) Lineage-aware internal consistency of known drug–cancer indications among predicted-sensitive patients. The complete set of 31 curated mappings was evaluated across all 9599 patients independently of the drug-set separation in (A). Points show Haldane–Anscombe-corrected log$_{2}$ odds ratios and horizontal lines show 95% confidence intervals for the eight mappings with FDR *<0.05* and an odds ratio *>1*. Colors and marker shapes identify drug mechanism modules, and log$_{2}$ odds ratio zero corresponds to an odds ratio of one.

Finally, we examined whether the model-derived patient rankings retained recognizable drug–cancer structure at the pan-cancer level. As shown in Fig. 7B, 8 of the 31 curated drug–cancer mappings met FDR *<0.05* and an odds ratio *>1* among the top 600 predicted-sensitive patients. Dabrafenib and Trametinib were positively enriched in melanoma, while Bleomycin, Mitoxantrone, Cytarabine, Tretinoin, Dactinomycin, and Dacarbazine were enriched in their corresponding indication cancer types. Thus, a subset of established lineage-associated drug–cancer relationships remained detectable in rankings derived from the predicted patient response profiles.

The threshold analysis recovered nested sets of 7, 8, and 9 mappings among the top 300, 600, and 1000 predicted-sensitive patients, respectively. Every association detected at the more restrictive threshold was retained as the ranked patient set expanded. The nested recovery across these cutoffs indicates continuity of the observed enrichment pattern as the ranked patient set expanded. These enrichments indicate that recognizable lineage-associated structure was retained in the model-derived patient rankings. Cancer-type comparability contributed to representation alignment, defining indication recovery as a lineage-aware internal consistency analysis.

## Discussion

TRACER integrates drug-response prediction, biologically conditioned neighbor alignment, and response-protected gradient regulation to align cell-line pharmacogenomic models with unlabeled patient tumors. CaDRReS [21], HiDRA [22], and GPDRP [23] focus on cell-line drug-response prediction. Celligner [19] and CellHit [20] construct cross-domain comparable spaces through independent alignment, whereas CODE-AE [24] and PANCDR [25] incorporate patient-domain information through shared representations or adversarial learning. TRACER uses the supervised drug-response objective to constrain alignment directly and connects cell-line and patient representations through biologically conditioned local neighborhoods. This end-to-end coupling differs from workflows in which alignment precedes response modeling. The source-domain results show that biologically conditioned alignment can be integrated while retaining competitive drug-response prediction.

Response-protected gradient regulation further clarifies the optimization relationship between the labeled primary task and the unlabeled auxiliary task. The ablation results connect this balance to asymmetric task prioritization, which confines gradient intervention to the auxiliary alignment objective and preserves the supervised response update. This design is suited to learning settings in which a directly labeled primary task is coupled to an unlabeled auxiliary transfer constraint.

Patient-level analyses characterized model-derived pharmacogenomic structure through complementary diagnostics. Hallmark neighbor consistency is an objective-aligned diagnostic because Hallmark activity contributes to the alignment condition. The mean improvement was heterogeneous across cancer types, supporting cancer-specific interpretation of the transferred representation. The source-validation-defined drug partition placed source test outcomes outside drug eligibility, quality ranking, and set construction. Stability-selected PRSs and their mechanism-level profiles provide cancer-specific computational hypotheses for subsequent functional and clinical evaluation. PRS-construction-excluded drugs characterize post-selection response structure within the trained drug space. Indication recovery assesses lineage-aware internal consistency because cancer-type comparability contributes to alignment.

TCGA lacks systematic patient-level treatment-response labels spanning multiple drugs and cancer types, so the present patient-side evidence characterizes computational pharmacological structure. The main TCGA atlas analysis was transductive; the split-specific alignment-excluded TCGA audit samples were reserved for Hallmark diagnostics. The uncertainty analysis showed underdispersion of the raw predictive standard deviation and improved marginal coverage after validation-set scalar calibration. Patient rankings remained stable after removal of the uncertainty penalty; BRCA showed sensitivity at the adjacent *k=5* resolution boundary. Independent clinical cohorts with well-defined treatment records and efficacy end points will enable direct evaluation of predicted response rankings. Patient-derived organoids and matched xenograft models can further support *in vitro* screening and *in vivo* validation of selected patient–drug associations [44, 45]. Drug-disjoint and scaffold-disjoint evaluation will be required to assess transfer to compounds absent from source-domain model training. Single-cell omics, spatial transcriptomics, and digital pathology may improve representation of tumor composition and microenvironmental heterogeneity [46, 47]. Cross-platform calibration guided by TRIPOD+AI principles will help define the scope of TRACER for patient drug-response analysis and functional precision oncology research [48].

## Conclusion

We developed TRACER as a unified framework integrating drug-response prediction, cross-domain representation alignment, and response-protected gradient regulation. The supervised response objective directly constrains alignment, while biologically conditioned neighborhoods connect cancer cell lines with unlabeled patient tumors through comparable local molecular contexts. Source-domain prediction, objective-aligned Hallmark diagnostics, and gradient-optimization analysis evaluated response preservation and cross-domain representation structure. Stability-guided PRS selection, characterization using PRS-construction-excluded drugs, and lineage-aware internal consistency analysis extended this computational evaluation to patient-level pharmacological structure. These predictive analyses generate testable patient–drug hypotheses for subsequent functional experiments and evaluation in independent clinical cohorts.

**Key Points**

- TRACER integrates supervised drug-response prediction, biologically conditioned cross-domain alignment, and response-protected gradient regulation within a unified end-to-end framework.
- TRACER-Full preserved strong source-domain drug-response prediction and improved mean oncogenic Hallmark neighbor consistency in alignment-excluded The Cancer Genome Atlas audit samples, with cancer-type heterogeneity.
- A source-validation-defined drug partition and uniform within-cancer stability analysis identified four model-derived pharmacological response subtypes in BRCA, four in UCEC and two in SARC.
- PRS-construction-excluded drugs characterized distinct mechanism-level response profiles across patient subtypes, and pan-cancer indication recovery assessed lineage-aware internal consistency in the predicted patient rankings.

## Supplementary Material

Supplementary_Figures_Part1_bbag593

Supplementary_Figures_Part2_bbag593

Supplementary_Figures_Part3_bbag593

Supplementary_Figures_Part4_bbag593

Supplementary_Figures_Part5_bbag593

Supplementary_Methods_and_Tables_bbag593

## Data Availability

Drug-response data were obtained from GDSC2 (https://cellmodelpassports.sanger.ac.uk/downloads); tumor molecular profiles and clinical annotations were obtained from TCGA through the Genomic Data Commons (https://portal.gdc.cancer.gov/); Hallmark gene sets were obtained from the Molecular Signatures Database (https://www.gsea-msigdb.org/gsea/msigdb); and drug–target and target–pathway relations were obtained from PrimeKG (https://github.com/mims-harvard/PrimeKG). These third-party resources are publicly accessible subject to their respective access and use conditions. Formal split assignments, processed summary tables underlying the reported results, and scripts for reconstructing the analysis-ready inputs are available through the TRACER GitHub repository. The TRACER source code, frozen configuration files, tests, documentation, split manifests, and a runnable demonstration dataset are available at https://github.com/wangdangdang01/TRACER. The code is released under the BSD-3-Clause license.
