# Supplementary material for: TRACER: response-protected cross-domain alignment of cancer pharmacogenomic models with patient tumors
Source: Brief Bioinform. 2026 Oct 28;27(5):bbag593. doi: 10.1093/bib/bbag593 (PMC13714793; doi:10.1093/bib/bbag593)
Supplement: Supplementary file 3 [file supplementary_figures_part3_bbag593.pdf]

Briefings in Bioinformatics, , pp. 1–7

Supplementary Figures

Supplementary Figures, Part 3: TRACER Patient Pharmacological Response Structures

Zhenxing Wang,[1] Qihe Wang,[1] Guangchao Wang,[1] Guohua Wang[2,1,*] and Yuming Zhao[1,*]

[1]College of Computer Science and Artificial Intelligence, Northeast Forestry University, Harbin 150040, China
[2]Department of Computer Science and Technology, Faculty of Computing, Harbin Institute of Technology, Harbin 150001, China

*Corresponding authors. Guohua Wang, Department of Computer Science and Technology, Faculty of Computing, Harbin Institute of Technology, Harbin 150001, China; College of Computer Science and Artificial Intelligence, Northeast Forestry University, Harbin 150040, China. E-mail: ghwang@nefu.edu.cn; Yuming Zhao, College of Computer Science and Artificial Intelligence, Northeast Forestry University, Harbin 150040, China. E-mail: zym@nefu.edu.cn.

© The Author(s) . Copyright and licence statements to be updated by the publisher during production.

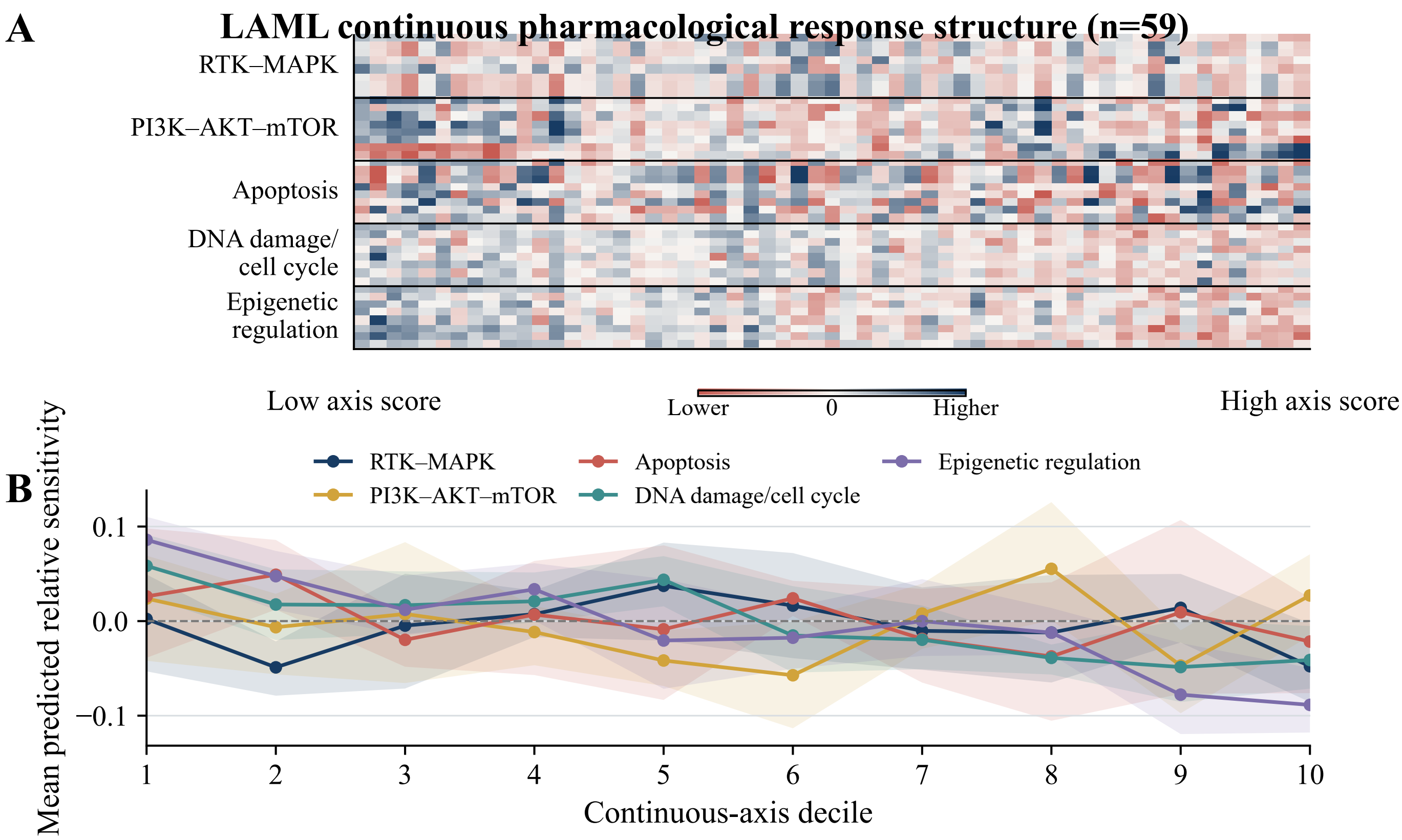

Figure S13 Continuous pharmacological response structure in LAML. A, PRS-construction-excluded-drug response heat map. Patients are ordered by the first principal-component score of the patient-centered 120-drug PRS-construction profile; the 40 displayed PRS-construction-excluded drugs did not contribute to this axis. **B,** Mean predicted relative sensitivity across five PRS-construction-excluded-drug mechanism modules in ten equal-frequency intervals of the continuous axis. Shaded bands denote 95% confidence intervals from 2,000 patient bootstraps. The axis direction is arbitrary and does not denote absolute sensitivity or resistance.

Alt text: Two-panel continuous pharmacological response structure in LAML. Panel A shows predicted responses to 40 PRS-construction-excluded drugs, with patients ordered by the first principal-component score derived from the 120-drug PRS-construction profile. Panel B shows mean predicted relative sensitivity across five drug-mechanism modules along ten equal-frequency intervals of the continuous axis, with 95% bootstrap confidence intervals.

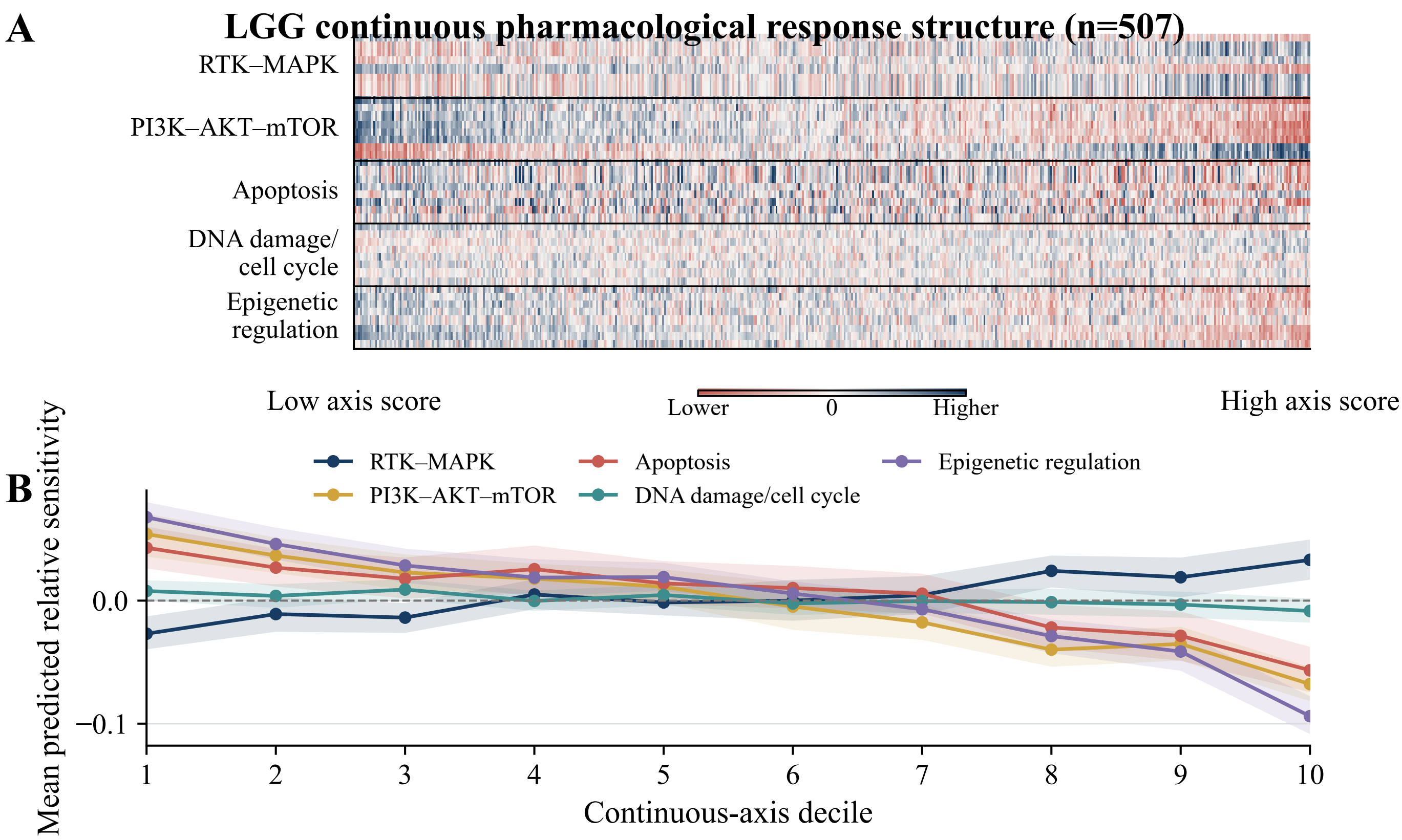

Figure S14 Continuous pharmacological response structure in LGG. A, PRS-construction-excluded-drug response heat map. Patients are ordered by the first principal-component score of the patient-centered 120-drug PRS-construction profile; the 40 displayed PRS-construction-excluded drugs did not contribute to this axis. **B,** Mean predicted relative sensitivity across five PRS-construction-excluded-drug mechanism modules in ten equal-frequency intervals of the continuous axis. Shaded bands denote 95% confidence intervals from 2,000 patient bootstraps. The axis direction is arbitrary and does not denote absolute sensitivity or resistance.

Alt text: Two-panel continuous pharmacological response structure in LGG. Panel A shows predicted responses to 40 PRS-construction-excluded drugs, with patients ordered by the first principal-component score derived from the 120-drug PRS-construction profile. Panel B shows mean predicted relative sensitivity across five drug-mechanism modules along ten equal-frequency intervals of the continuous axis, with 95% bootstrap confidence intervals.

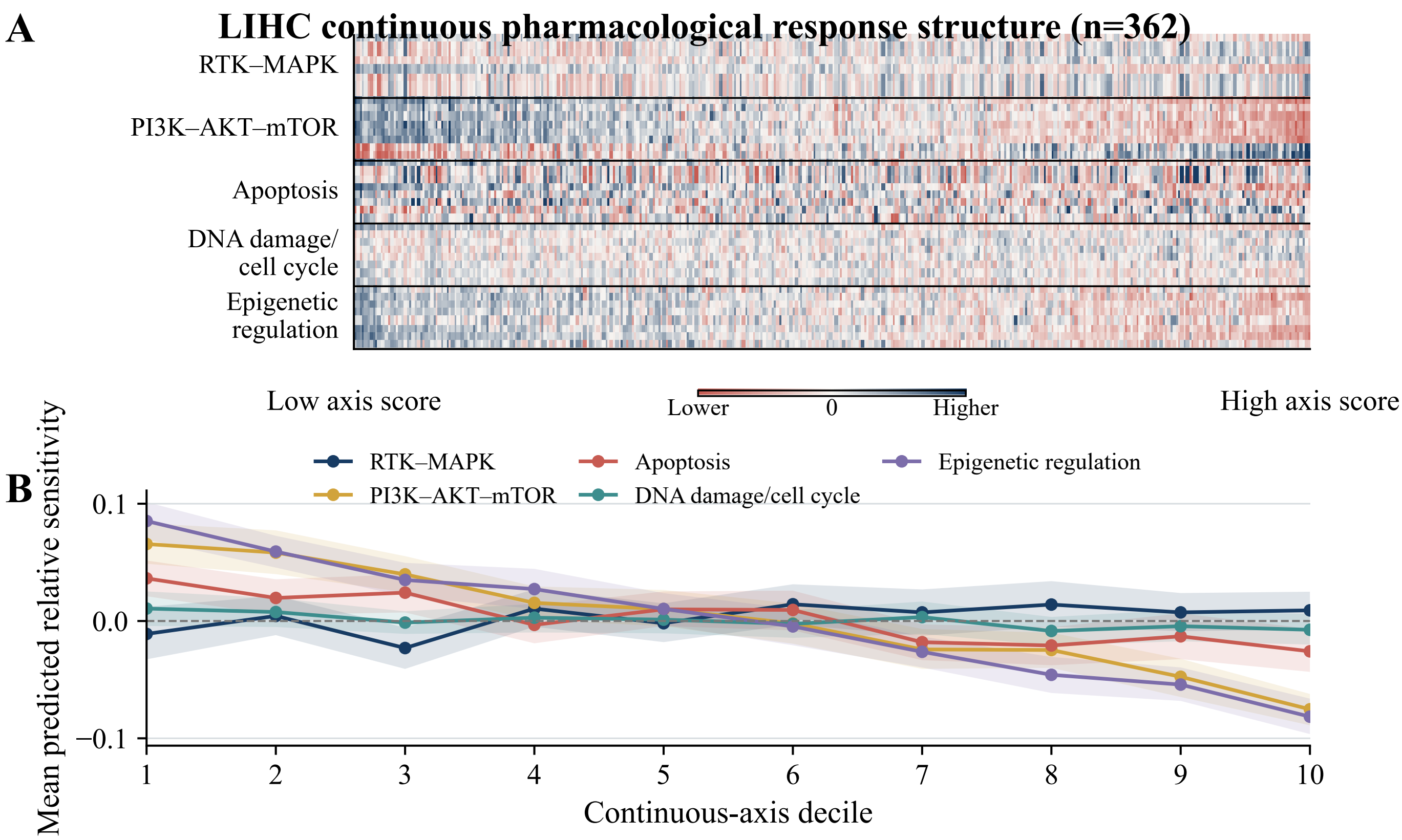

Figure S15 Continuous pharmacological response structure in LIHC. A, PRS-construction-excluded-drug response heat map. Patients are ordered by the first principal-component score of the patient-centered 120-drug PRS-construction profile; the 40 displayed PRS-construction-excluded drugs did not contribute to this axis. **B,** Mean predicted relative sensitivity across five PRS-construction-excluded-drug mechanism modules in ten equal-frequency intervals of the continuous axis. Shaded bands denote 95% confidence intervals from 2,000 patient bootstraps. The axis direction is arbitrary and does not denote absolute sensitivity or resistance.

Alt text: Two-panel continuous pharmacological response structure in LIHC. Panel A shows predicted responses to 40 PRS-construction-excluded drugs, with patients ordered by the first principal-component score derived from the 120-drug PRS-construction profile. Panel B shows mean predicted relative sensitivity across five drug-mechanism modules along ten equal-frequency intervals of the continuous axis, with 95% bootstrap confidence intervals.

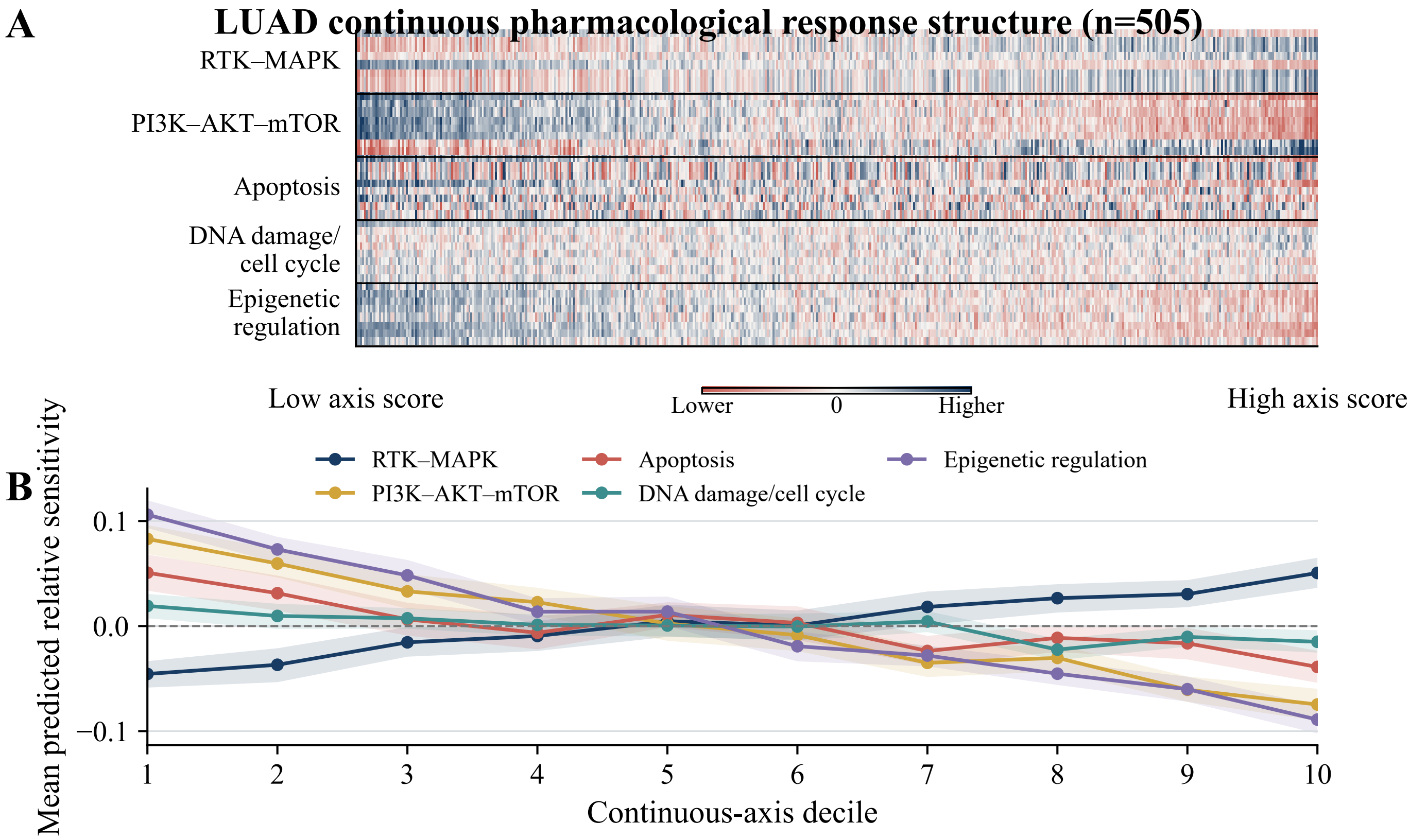

Figure S16 Continuous pharmacological response structure in LUAD. A, PRS-construction-excluded-drug response heat map. Patients are ordered by the first principal-component score of the patient-centered 120-drug PRS-construction profile; the 40 displayed PRS-construction-excluded drugs did not contribute to this axis. **B,** Mean predicted relative sensitivity across five PRS-construction-excluded-drug mechanism modules in ten equal-frequency intervals of the continuous axis. Shaded bands denote 95% confidence intervals from 2,000 patient bootstraps. The axis direction is arbitrary and does not denote absolute sensitivity or resistance.

Alt text: Two-panel continuous pharmacological response structure in LUAD. Panel A shows predicted responses to 40 PRS-construction-excluded drugs, with patients ordered by the first principal-component score derived from the 120-drug PRS-construction profile. Panel B shows mean predicted relative sensitivity across five drug-mechanism modules along ten equal-frequency intervals of the continuous axis, with 95% bootstrap confidence intervals.

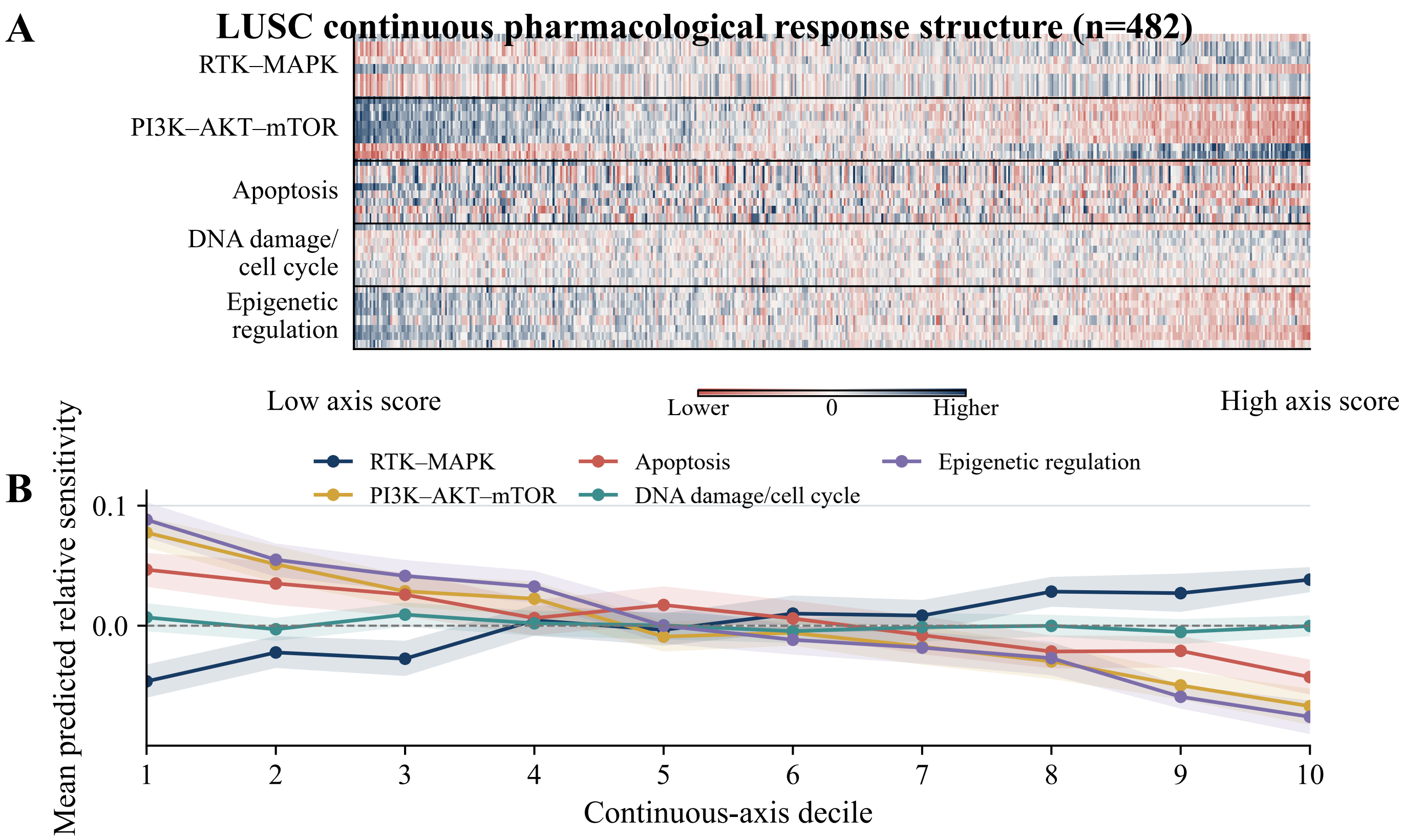

Figure S17 Continuous pharmacological response structure in LUSC. A, PRS-construction-excluded-drug response heat map. Patients are ordered by the first principal-component score of the patient-centered 120-drug PRS-construction profile; the 40 displayed PRS-construction-excluded drugs did not contribute to this axis. **B,** Mean predicted relative sensitivity across five PRS-construction-excluded-drug mechanism modules in ten equal-frequency intervals of the continuous axis. Shaded bands denote 95% confidence intervals from 2,000 patient bootstraps. The axis direction is arbitrary and does not denote absolute sensitivity or resistance.

Alt text: Two-panel continuous pharmacological response structure in LUSC. Panel A shows predicted responses to 40 PRS-construction-excluded drugs, with patients ordered by the first principal-component score derived from the 120-drug PRS-construction profile. Panel B shows mean predicted relative sensitivity across five drug-mechanism modules along ten equal-frequency intervals of the continuous axis, with 95% bootstrap confidence intervals.

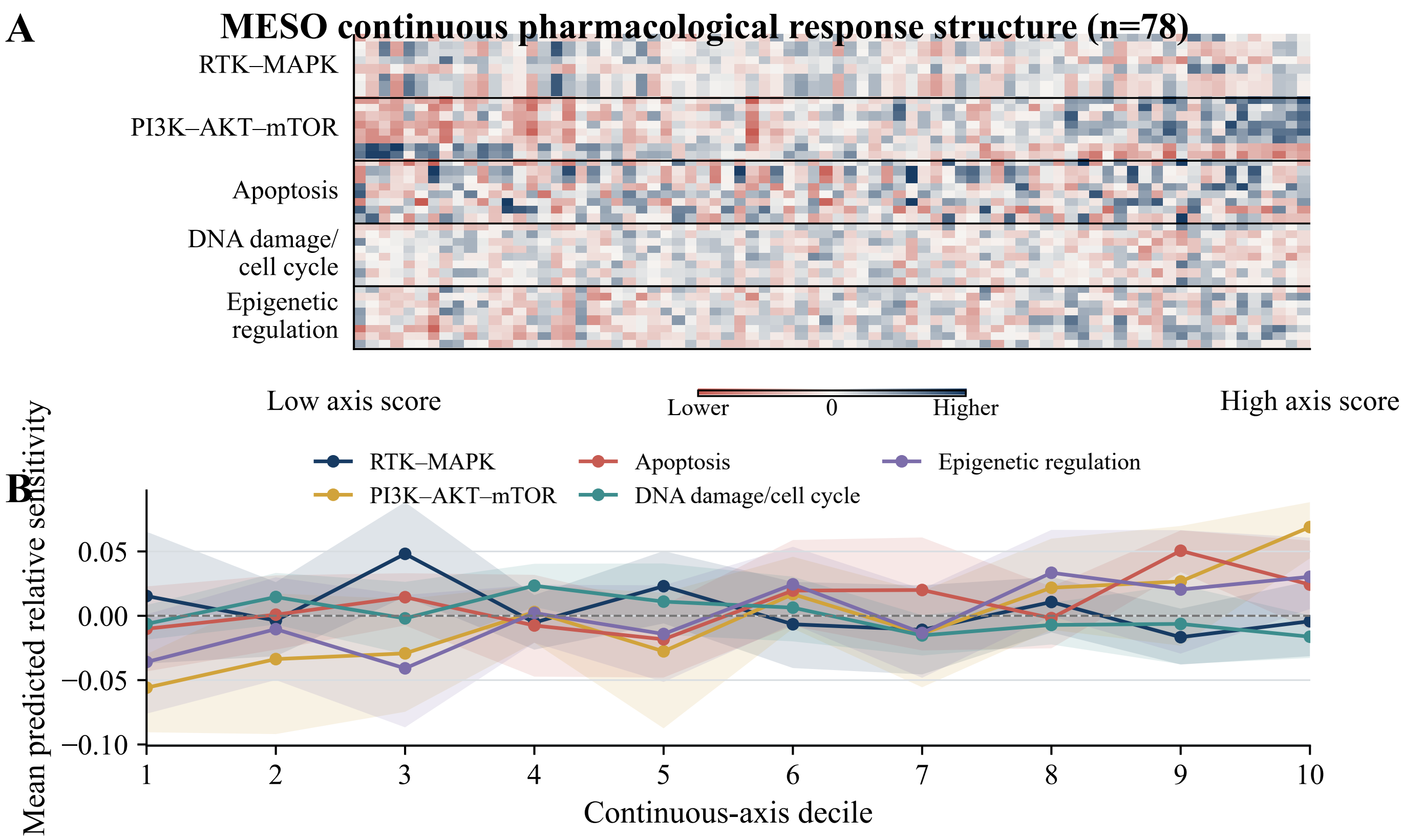

Figure S18 Continuous pharmacological response structure in MESO. A, PRS-construction-excluded-drug response heat map. Patients are ordered by the first principal-component score of the patient-centered 120-drug PRS-construction profile; the 40 displayed PRS-construction-excluded drugs did not contribute to this axis. **B,** Mean predicted relative sensitivity across five PRS-construction-excluded-drug mechanism modules in ten equal-frequency intervals of the continuous axis. Shaded bands denote 95% confidence intervals from 2,000 patient bootstraps. The axis direction is arbitrary and does not denote absolute sensitivity or resistance.

Alt text: Two-panel continuous pharmacological response structure in MESO. Panel A shows predicted responses to 40 PRS-construction-excluded drugs, with patients ordered by the first principal-component score derived from the 120-drug PRS-construction profile. Panel B shows mean predicted relative sensitivity across five drug-mechanism modules along ten equal-frequency intervals of the continuous axis, with 95% bootstrap confidence intervals.
