# Supplementary material for: TRACER: response-protected cross-domain alignment of cancer pharmacogenomic models with patient tumors
Source: Brief Bioinform. 2026 Oct 28;27(5):bbag593. doi: 10.1093/bib/bbag593 (PMC13714793; doi:10.1093/bib/bbag593)
Supplement: Supplementary file 4 [file supplementary_figures_part4_bbag593.pdf]

Briefings in Bioinformatics, , pp. 1–7

Supplementary Figures

Supplementary Figures, Part 4: TRACER Patient Pharmacological Response Structures

Zhenxing Wang,[1] Qihe Wang,[1] Guangchao Wang,[1] Guohua Wang[2,1,*] and Yuming Zhao[1,*]

[1]College of Computer Science and Artificial Intelligence, Northeast Forestry University, Harbin 150040, China
[2]Department of Computer Science and Technology, Faculty of Computing, Harbin Institute of Technology, Harbin 150001, China

[*]Corresponding authors. Guohua Wang, Department of Computer Science and Technology, Faculty of Computing, Harbin Institute of Technology, Harbin 150001, China; College of Computer Science and Artificial Intelligence, Northeast Forestry University, Harbin 150040, China. E-mail: ghwang@nefu.edu.cn; Yuming Zhao, College of Computer Science and Artificial Intelligence, Northeast Forestry University, Harbin 150040, China. E-mail: zym@nefu.edu.cn.

© The Author(s) . Copyright and licence statements to be updated by the publisher during production.

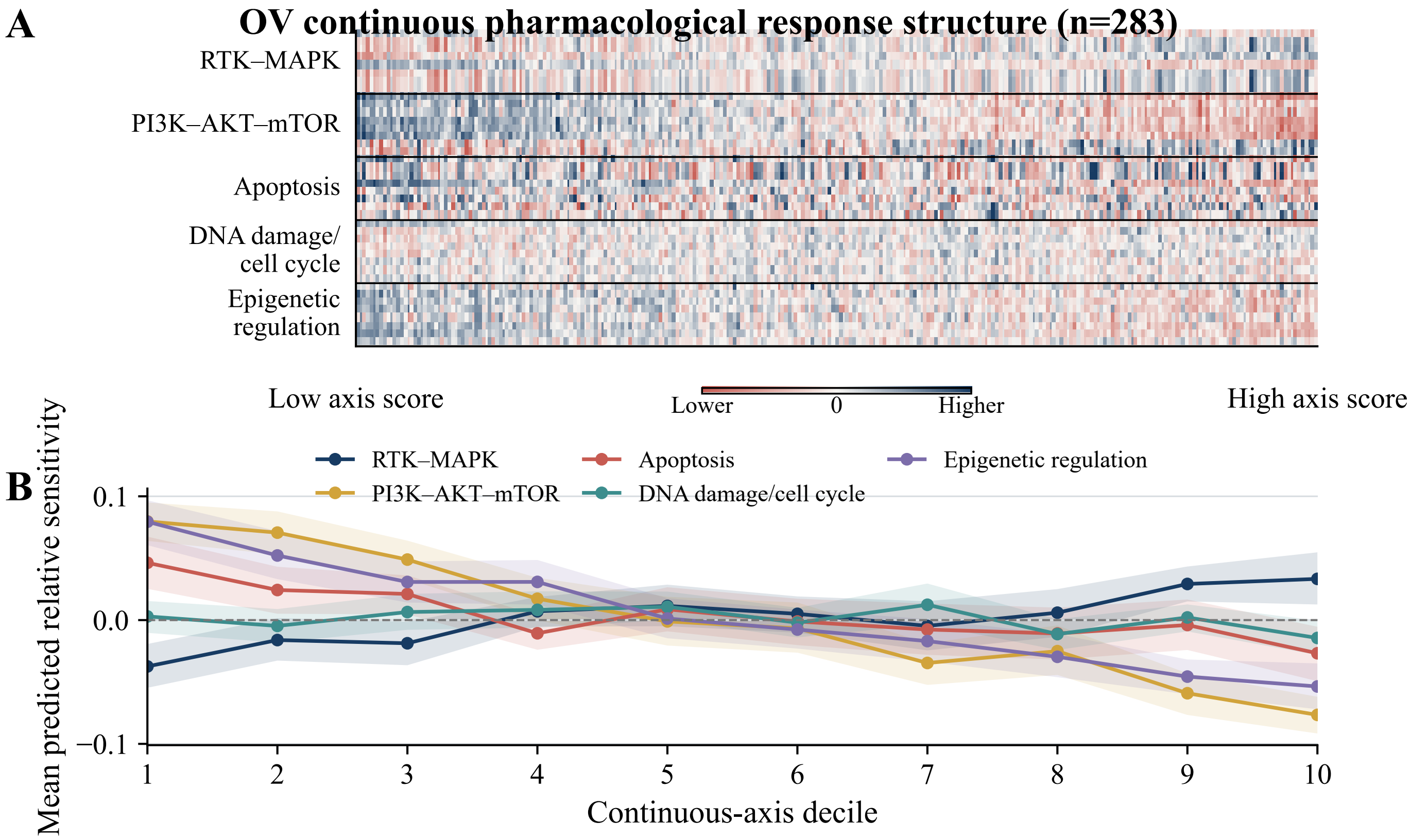

Figure S19 Continuous pharmacological response structure in OV. A, PRS-construction-excluded-drug response heat map. Patients are ordered by the first principal-component score of the patient-centered 120-drug PRS-construction profile; the 40 displayed PRS-construction-excluded drugs did not contribute to this axis. **B,** Mean predicted relative sensitivity across five PRS-construction-excluded-drug mechanism modules in ten equal-frequency intervals of the continuous axis. Shaded bands denote 95% confidence intervals from 2,000 patient bootstraps. The axis direction is arbitrary and does not denote absolute sensitivity or resistance.

Alt text: Two-panel continuous pharmacological response structure in OV. Panel A shows predicted responses to 40 PRS-construction-excluded drugs, with patients ordered by the first principal-component score derived from the 120-drug PRS-construction profile. Panel B shows mean predicted relative sensitivity across five drug-mechanism modules along ten equal-frequency intervals of the continuous axis, with 95% bootstrap confidence intervals.

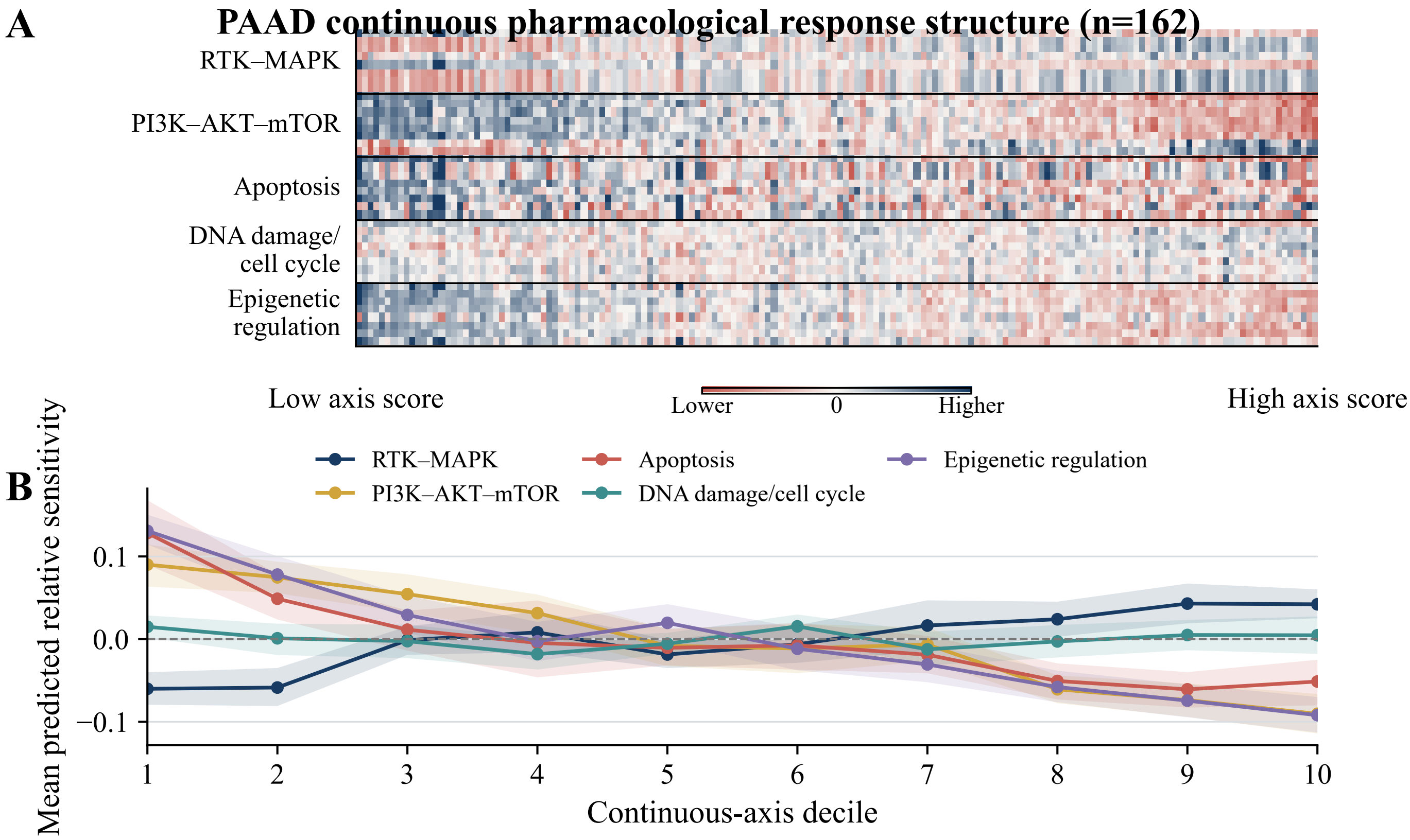

Figure S20 Continuous pharmacological response structure in PAAD. A, PRS-construction-excluded-drug response heat map. Patients are ordered by the first principal-component score of the patient-centered 120-drug PRS-construction profile; the 40 displayed PRS-construction-excluded drugs did not contribute to this axis. **B,** Mean predicted relative sensitivity across five PRS-construction-excluded-drug mechanism modules in ten equal-frequency intervals of the continuous axis. Shaded bands denote 95% confidence intervals from 2,000 patient bootstraps. The axis direction is arbitrary and does not denote absolute sensitivity or resistance.

Alt text: Two-panel continuous pharmacological response structure in PAAD. Panel A shows predicted responses to 40 PRS-construction-excluded drugs, with patients ordered by the first principal-component score derived from the 120-drug PRS-construction profile. Panel B shows mean predicted relative sensitivity across five drug-mechanism modules along ten equal-frequency intervals of the continuous axis, with 95% bootstrap confidence intervals.

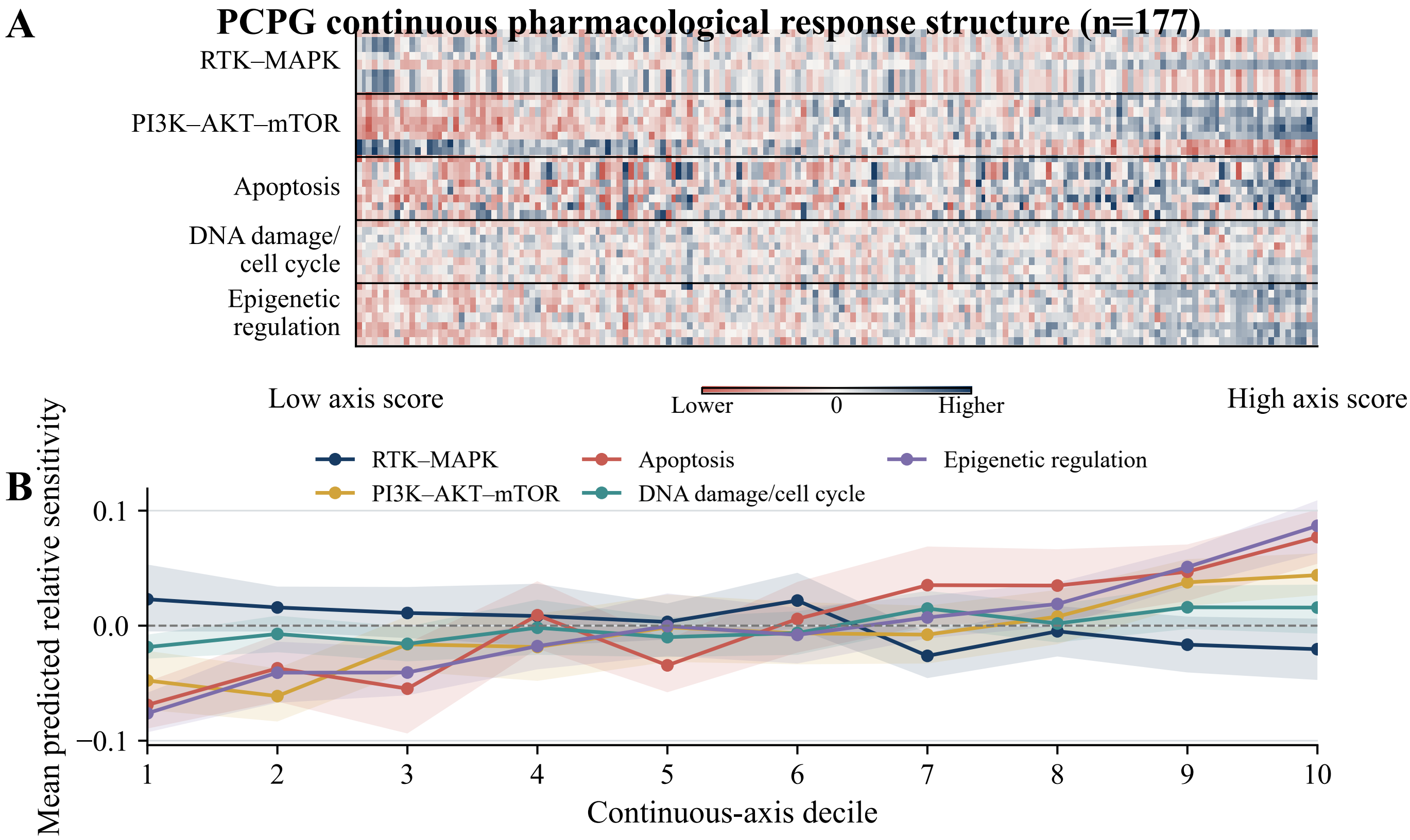

Figure S21 Continuous pharmacological response structure in PCPG. A, PRS-construction-excluded-drug response heat map. Patients are ordered by the first principal-component score of the patient-centered 120-drug PRS-construction profile; the 40 displayed PRS-construction-excluded drugs did not contribute to this axis. **B,** Mean predicted relative sensitivity across five PRS-construction-excluded-drug mechanism modules in ten equal-frequency intervals of the continuous axis. Shaded bands denote 95% confidence intervals from 2,000 patient bootstraps. The axis direction is arbitrary and does not denote absolute sensitivity or resistance.

Alt text: Two-panel continuous pharmacological response structure in PCPG. Panel A shows predicted responses to 40 PRS-construction-excluded drugs, with patients ordered by the first principal-component score derived from the 120-drug PRS-construction profile. Panel B shows mean predicted relative sensitivity across five drug-mechanism modules along ten equal-frequency intervals of the continuous axis, with 95% bootstrap confidence intervals.

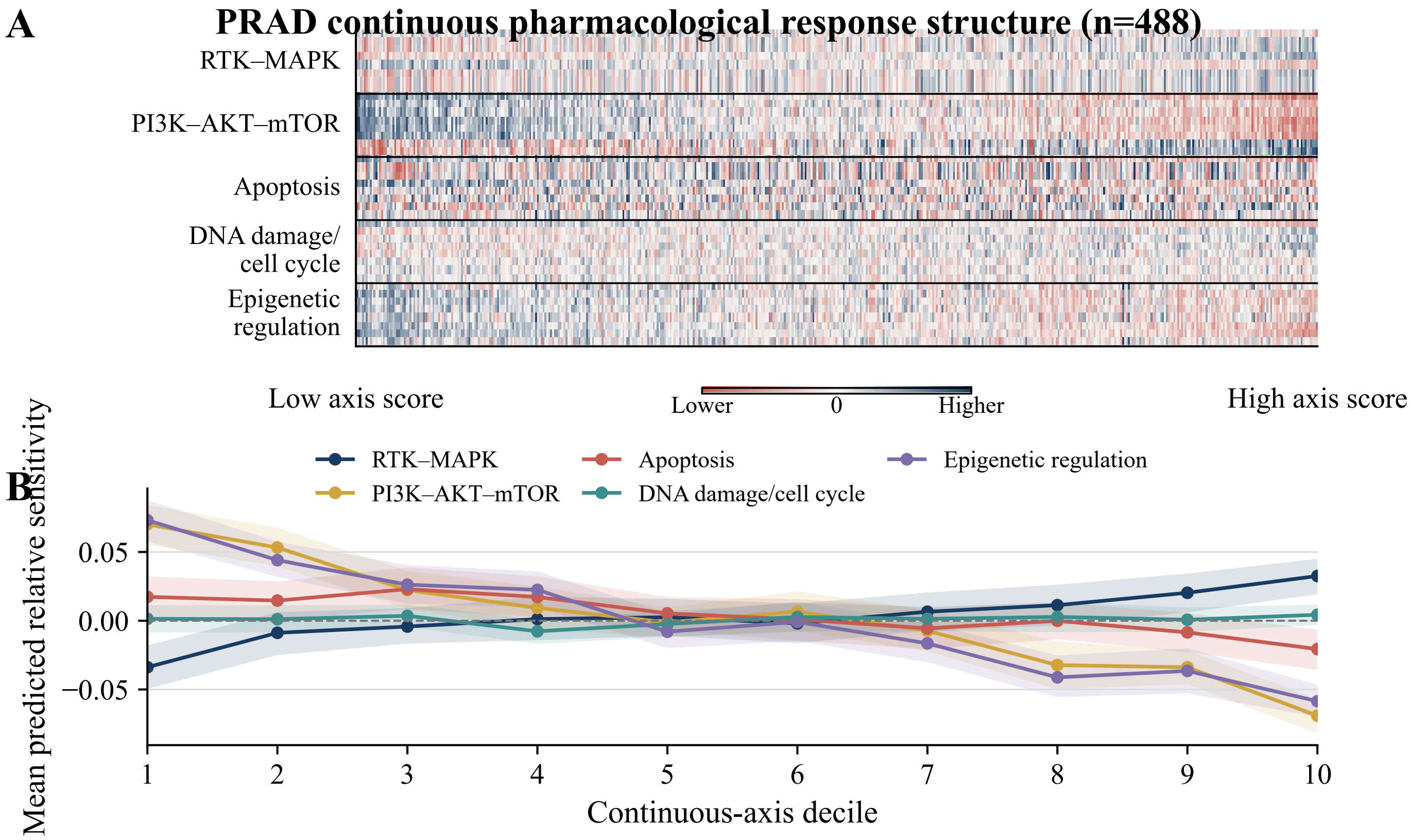

Figure S22 Continuous pharmacological response structure in PRAD. A, PRS-construction-excluded-drug response heat map. Patients are ordered by the first principal-component score of the patient-centered 120-drug PRS-construction profile; the 40 displayed PRS-construction-excluded drugs did not contribute to this axis. **B,** Mean predicted relative sensitivity across five PRS-construction-excluded-drug mechanism modules in ten equal-frequency intervals of the continuous axis. Shaded bands denote 95% confidence intervals from 2,000 patient bootstraps. The axis direction is arbitrary and does not denote absolute sensitivity or resistance.

Alt text: Two-panel continuous pharmacological response structure in PRAD. Panel A shows predicted responses to 40 PRS-construction-excluded drugs, with patients ordered by the first principal-component score derived from the 120-drug PRS-construction profile. Panel B shows mean predicted relative sensitivity across five drug-mechanism modules along ten equal-frequency intervals of the continuous axis, with 95% bootstrap confidence intervals.

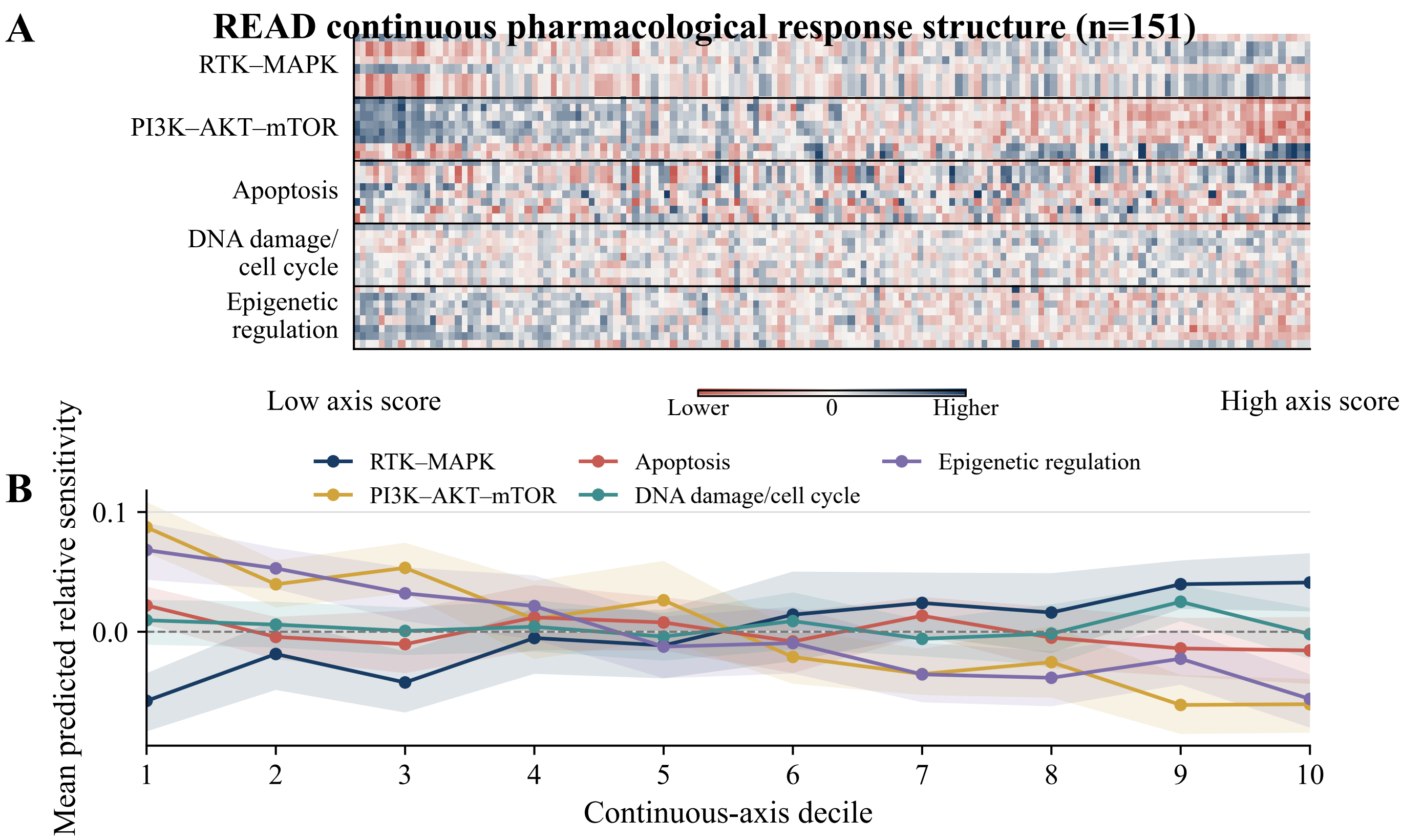

Figure S23 Continuous pharmacological response structure in READ. A, PRS-construction-excluded-drug response heat map. Patients are ordered by the first principal-component score of the patient-centered 120-drug PRS-construction profile; the 40 displayed PRS-construction-excluded drugs did not contribute to this axis. **B,** Mean predicted relative sensitivity across five PRS-construction-excluded-drug mechanism modules in ten equal-frequency intervals of the continuous axis. Shaded bands denote 95% confidence intervals from 2,000 patient bootstraps. The axis direction is arbitrary and does not denote absolute sensitivity or resistance.

Alt text: Two-panel continuous pharmacological response structure in READ. Panel A shows predicted responses to 40 PRS-construction-excluded drugs, with patients ordered by the first principal-component score derived from the 120-drug PRS-construction profile. Panel B shows mean predicted relative sensitivity across five drug-mechanism modules along ten equal-frequency intervals of the continuous axis, with 95% bootstrap confidence intervals.

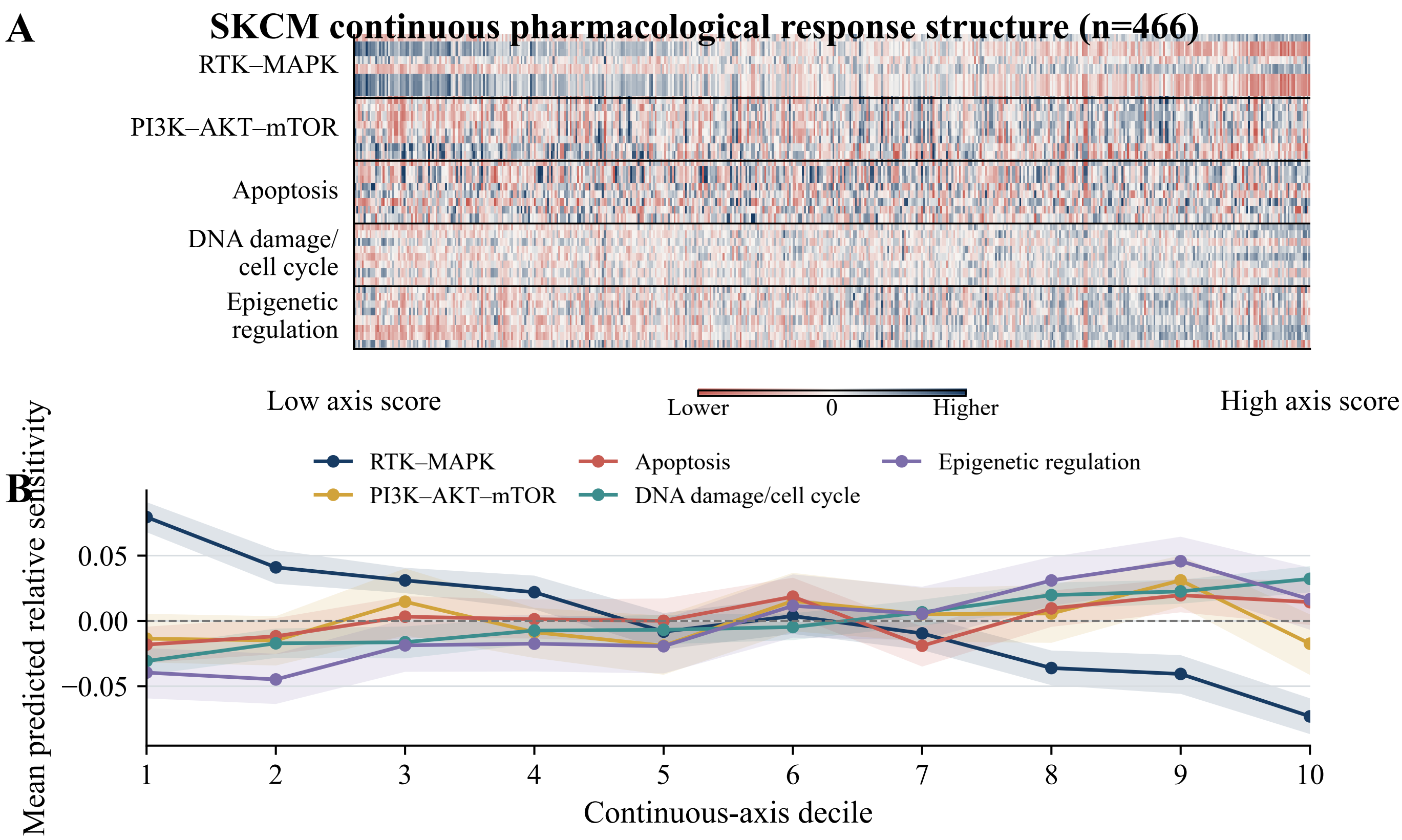

Figure S24 Continuous pharmacological response structure in SKCM. A, PRS-construction-excluded-drug response heat map. Patients are ordered by the first principal-component score of the patient-centered 120-drug PRS-construction profile; the 40 displayed PRS-construction-excluded drugs did not contribute to this axis. **B,** Mean predicted relative sensitivity across five PRS-construction-excluded-drug mechanism modules in ten equal-frequency intervals of the continuous axis. Shaded bands denote 95% confidence intervals from 2,000 patient bootstraps. The axis direction is arbitrary and does not denote absolute sensitivity or resistance.

Alt text: Two-panel continuous pharmacological response structure in SKCM. Panel A shows predicted responses to 40 PRS-construction-excluded drugs, with patients ordered by the first principal-component score derived from the 120-drug PRS-construction profile. Panel B shows mean predicted relative sensitivity across five drug-mechanism modules along ten equal-frequency intervals of the continuous axis, with 95% bootstrap confidence intervals.
