# Supplementary material for: TRACER: response-protected cross-domain alignment of cancer pharmacogenomic models with patient tumors
Source: Brief Bioinform. 2026 Oct 28;27(5):bbag593. doi: 10.1093/bib/bbag593 (PMC13714793; doi:10.1093/bib/bbag593)
Supplement: Supplementary file 5 [file supplementary_figures_part5_bbag593.pdf]

Briefings in Bioinformatics, , pp. 1–8

Supplementary Figures

Supplementary Figures, Part 5: TRACER Patient Pharmacological Response Structures

Zhenxing Wang[1] Qihe Wang[1] Guangchao Wang[1] Guohua Wang[2,1,*] and Yuming Zhao[1,*]

[1]College of Computer Science and Artificial Intelligence, Northeast Forestry University, Harbin 150040, China

[2]Department of Computer Science and Technology, Faculty of Computing, Harbin Institute of Technology, Harbin 150001, China

[*]Corresponding authors. Guohua Wang, Department of Computer Science and Technology, Faculty of Computing, Harbin Institute of Technology, Harbin 150001, China; College of Computer Science and Artificial Intelligence, Northeast Forestry University, Harbin 150040, China. E-mail: ghwang@nefu.edu.cn; Yuming Zhao, College of Computer Science and Artificial Intelligence, Northeast Forestry University, Harbin 150040, China. E-mail: zym@nefu.edu.cn.

© The Author(s) . Copyright and licence statements to be updated by the publisher during production.

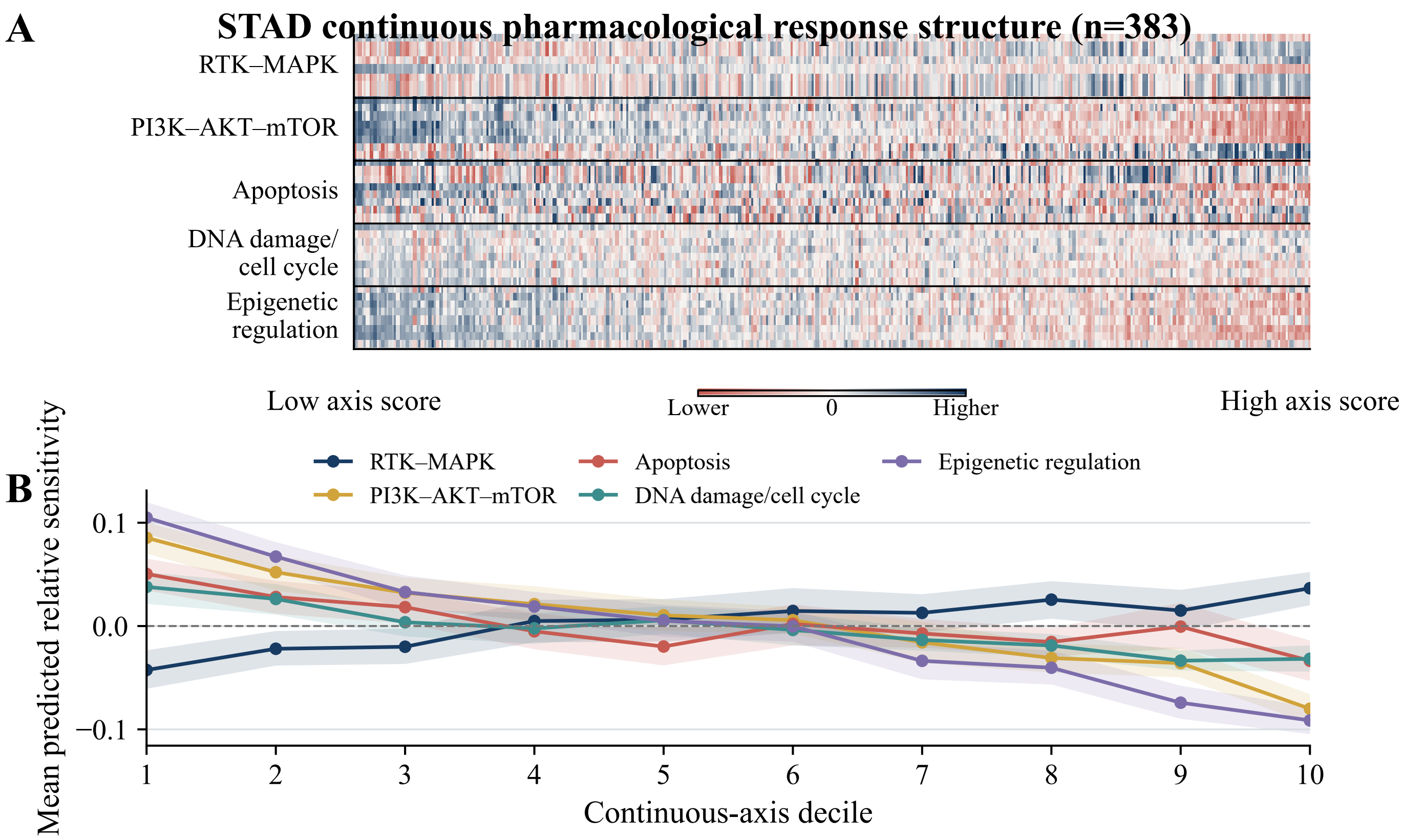

Figure S25 Continuous pharmacological response structure in STAD. A, PRS-construction-excluded-drug response heat map. Patients are ordered by the first principal-component score of the patient-centered 120-drug PRS-construction profile; the 40 displayed PRS-construction-excluded drugs did not contribute to this axis. **B,** Mean predicted relative sensitivity across five PRS-construction-excluded-drug mechanism modules in ten equal-frequency intervals of the continuous axis. Shaded bands denote 95% confidence intervals from 2,000 patient bootstraps. The axis direction is arbitrary and does not denote absolute sensitivity or resistance.

Alt text: Two-panel continuous pharmacological response structure in STAD. Panel A shows predicted responses to 40 PRS-construction-excluded drugs, with patients ordered by the first principal-component score derived from the 120-drug PRS-construction profile. Panel B shows mean predicted relative sensitivity across five drug-mechanism modules along ten equal-frequency intervals of the continuous axis, with 95% bootstrap confidence intervals.

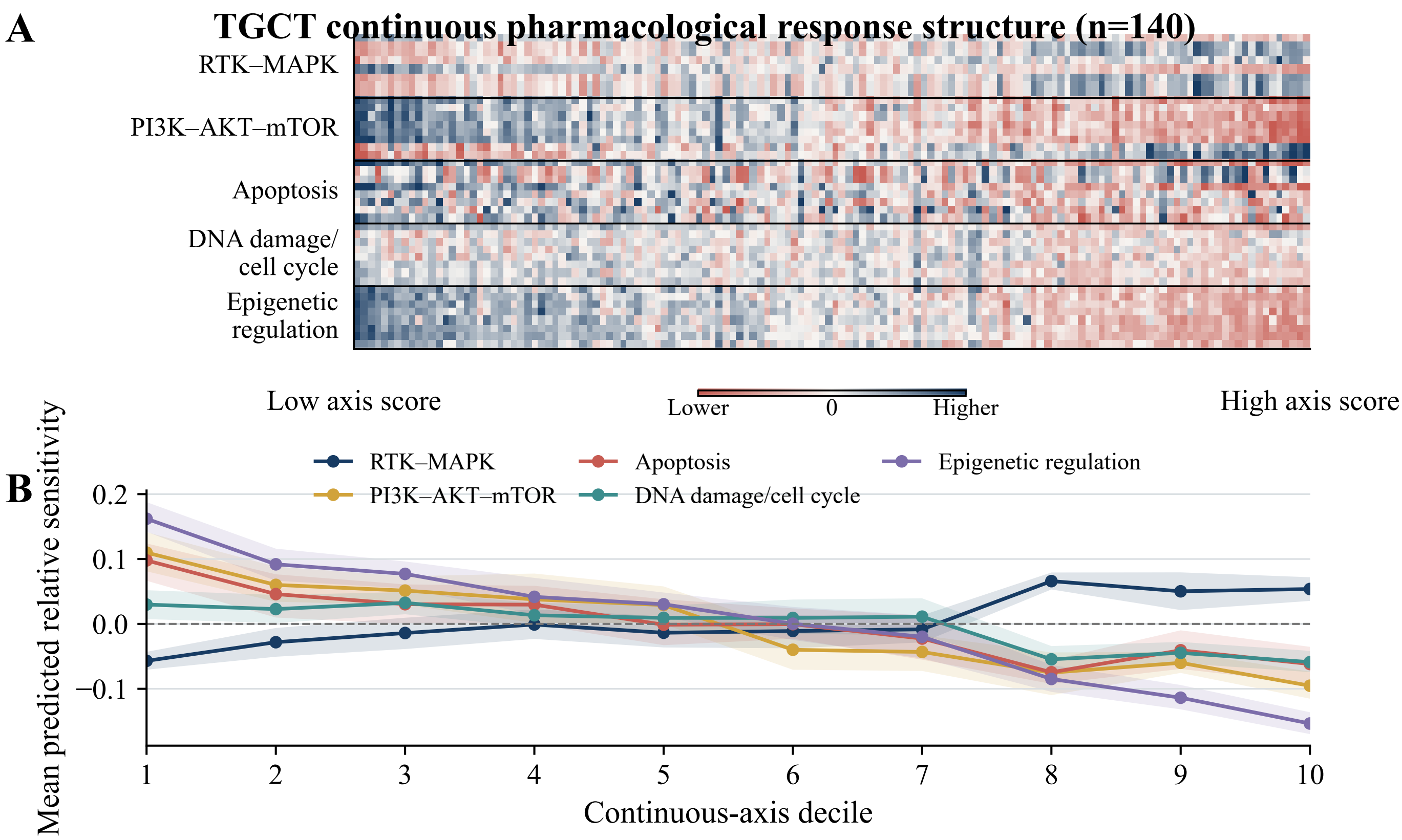

Figure S26 Continuous pharmacological response structure in TGCT. A, PRS-construction-excluded-drug response heat map. Patients are ordered by the first principal-component score of the patient-centered 120-drug PRS-construction profile; the 40 displayed PRS-construction-excluded drugs did not contribute to this axis. **B,** Mean predicted relative sensitivity across five PRS-construction-excluded-drug mechanism modules in ten equal-frequency intervals of the continuous axis. Shaded bands denote 95% confidence intervals from 2,000 patient bootstraps. The axis direction is arbitrary and does not denote absolute sensitivity or resistance.

Alt text: Two-panel continuous pharmacological response structure in TGCT. Panel A shows predicted responses to 40 PRS-construction-excluded drugs, with patients ordered by the first principal-component score derived from the 120-drug PRS-construction profile. Panel B shows mean predicted relative sensitivity across five drug-mechanism modules along ten equal-frequency intervals of the continuous axis, with 95% bootstrap confidence intervals.

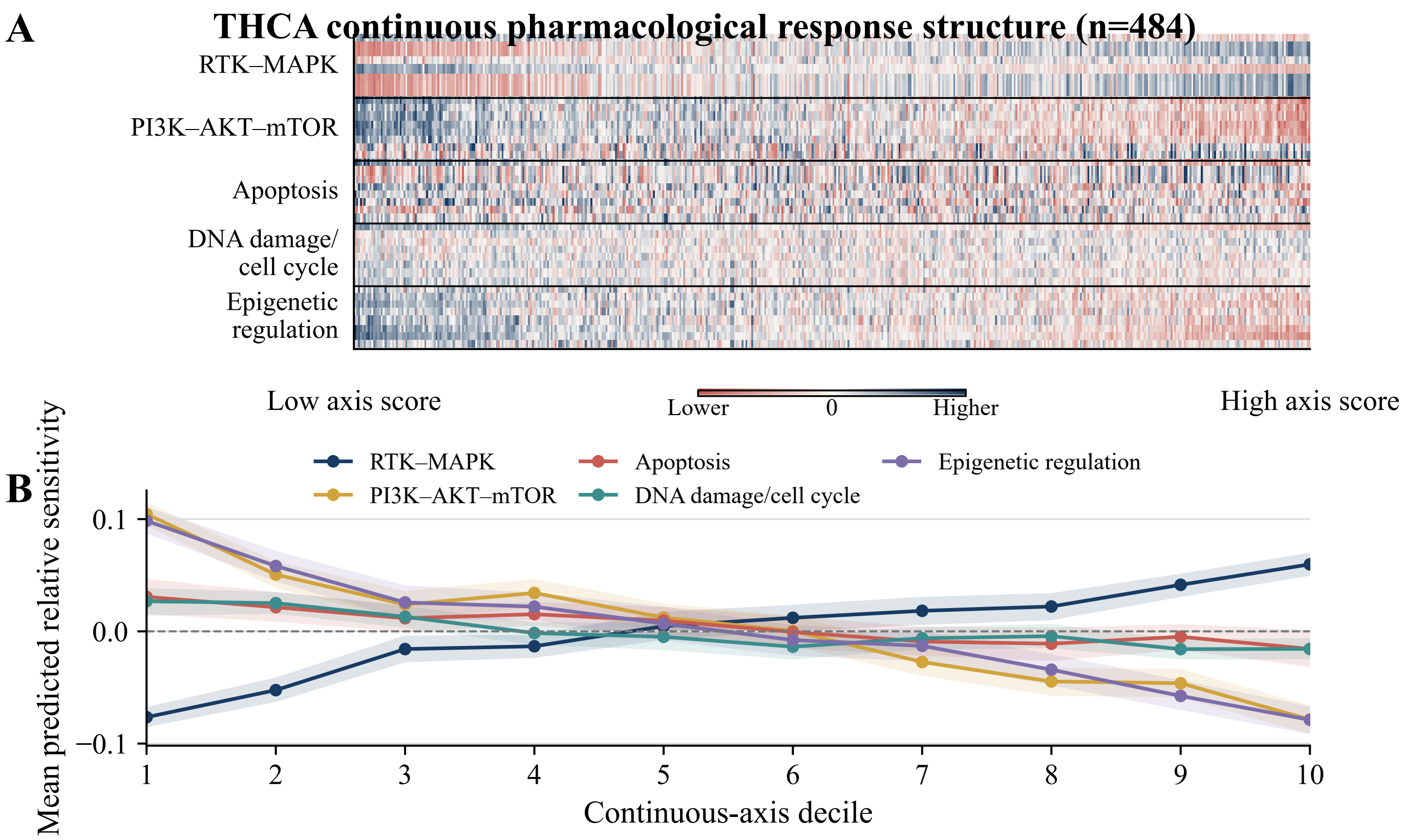

Figure S27 Continuous pharmacological response structure in THCA. A, PRS-construction-excluded-drug response heat map. Patients are ordered by the first principal-component score of the patient-centered 120-drug PRS-construction profile; the 40 displayed PRS-construction-excluded drugs did not contribute to this axis. **B,** Mean predicted relative sensitivity across five PRS-construction-excluded-drug mechanism modules in ten equal-frequency intervals of the continuous axis. Shaded bands denote 95% confidence intervals from 2,000 patient bootstraps. The axis direction is arbitrary and does not denote absolute sensitivity or resistance.

Alt text: Two-panel continuous pharmacological response structure in THCA. Panel A shows predicted responses to 40 PRS-construction-excluded drugs, with patients ordered by the first principal-component score derived from the 120-drug PRS-construction profile. Panel B shows mean predicted relative sensitivity across five drug-mechanism modules along ten equal-frequency intervals of the continuous axis, with 95% bootstrap confidence intervals.

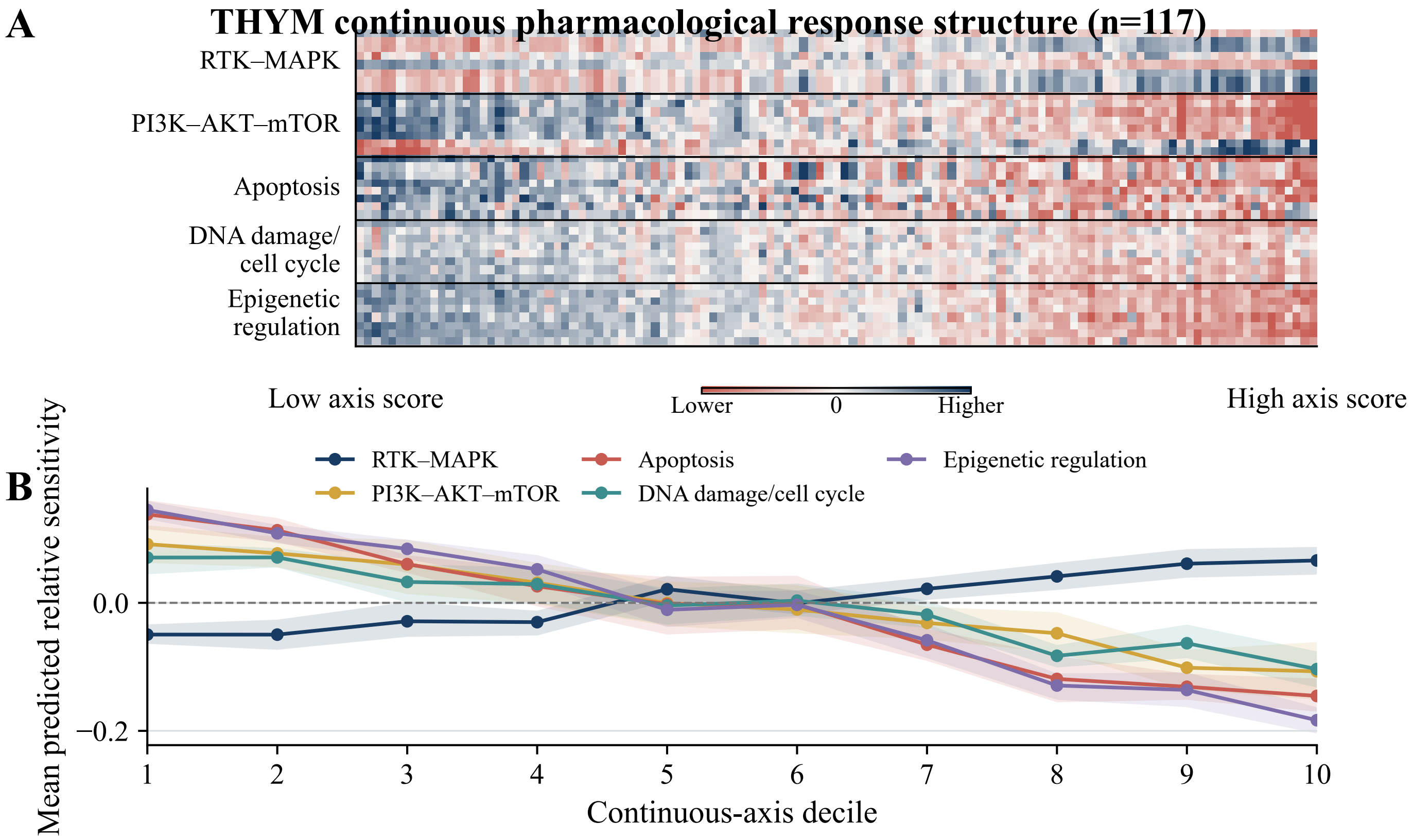

Figure S28 Continuous pharmacological response structure in THYM. A, PRS-construction-excluded-drug response heat map. Patients are ordered by the first principal-component score of the patient-centered 120-drug PRS-construction profile; the 40 displayed PRS-construction-excluded drugs did not contribute to this axis. **B,** Mean predicted relative sensitivity across five PRS-construction-excluded-drug mechanism modules in ten equal-frequency intervals of the continuous axis. Shaded bands denote 95% confidence intervals from 2,000 patient bootstraps. The axis direction is arbitrary and does not denote absolute sensitivity or resistance.

Alt text: Two-panel continuous pharmacological response structure in THYM. Panel A shows predicted responses to 40 PRS-construction-excluded drugs, with patients ordered by the first principal-component score derived from the 120-drug PRS-construction profile. Panel B shows mean predicted relative sensitivity across five drug-mechanism modules along ten equal-frequency intervals of the continuous axis, with 95% bootstrap confidence intervals.

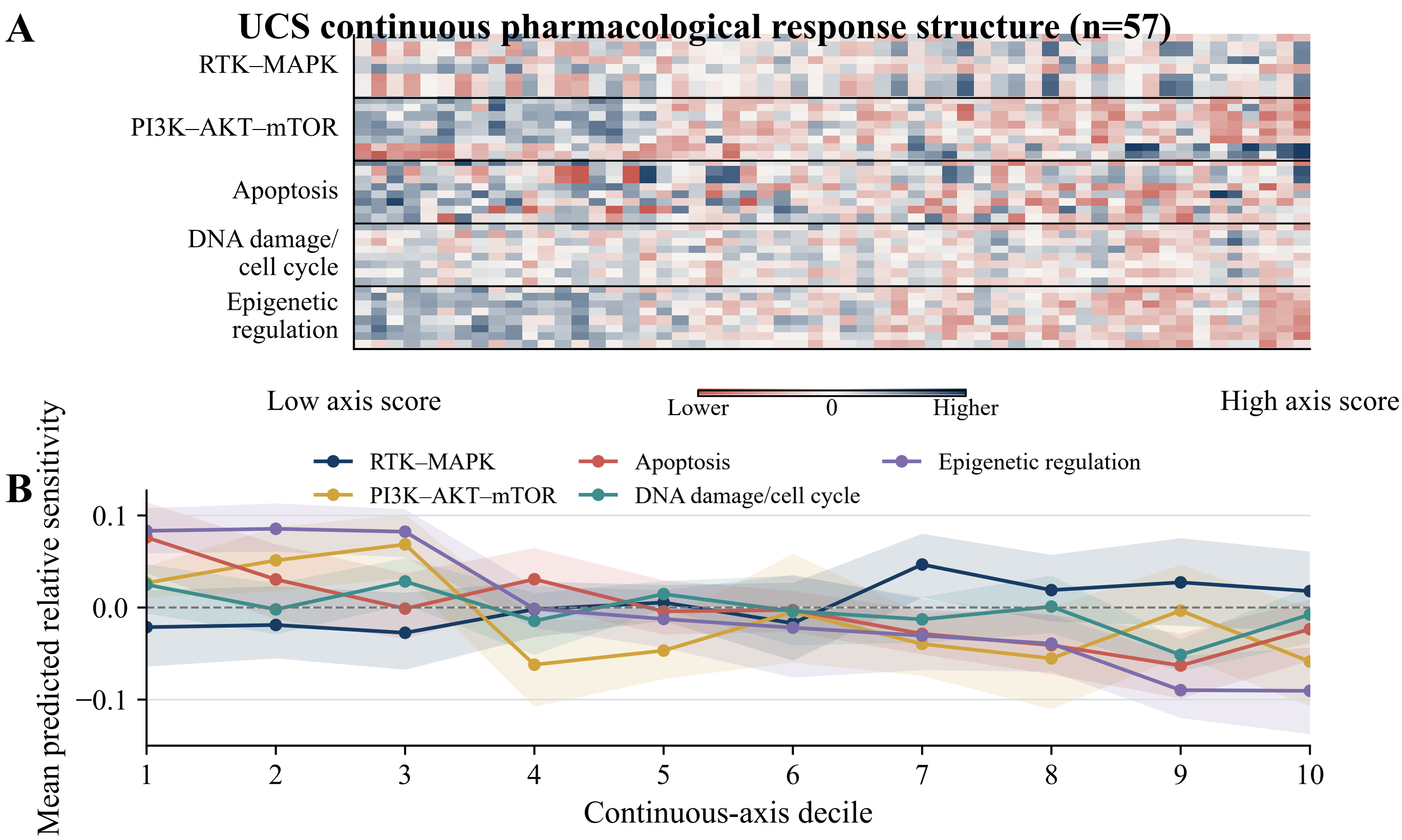

Figure S29 Continuous pharmacological response structure in UCS. A, PRS-construction-excluded-drug response heat map. Patients are ordered by the first principal-component score of the patient-centered 120-drug PRS-construction profile; the 40 displayed PRS-construction-excluded drugs did not contribute to this axis. **B,** Mean predicted relative sensitivity across five PRS-construction-excluded-drug mechanism modules in ten equal-frequency intervals of the continuous axis. Shaded bands denote 95% confidence intervals from 2,000 patient bootstraps. The axis direction is arbitrary and does not denote absolute sensitivity or resistance.

Alt text: Two-panel continuous pharmacological response structure in UCS. Panel A shows predicted responses to 40 PRS-construction-excluded drugs, with patients ordered by the first principal-component score derived from the 120-drug PRS-construction profile. Panel B shows mean predicted relative sensitivity across five drug-mechanism modules along ten equal-frequency intervals of the continuous axis, with 95% bootstrap confidence intervals.

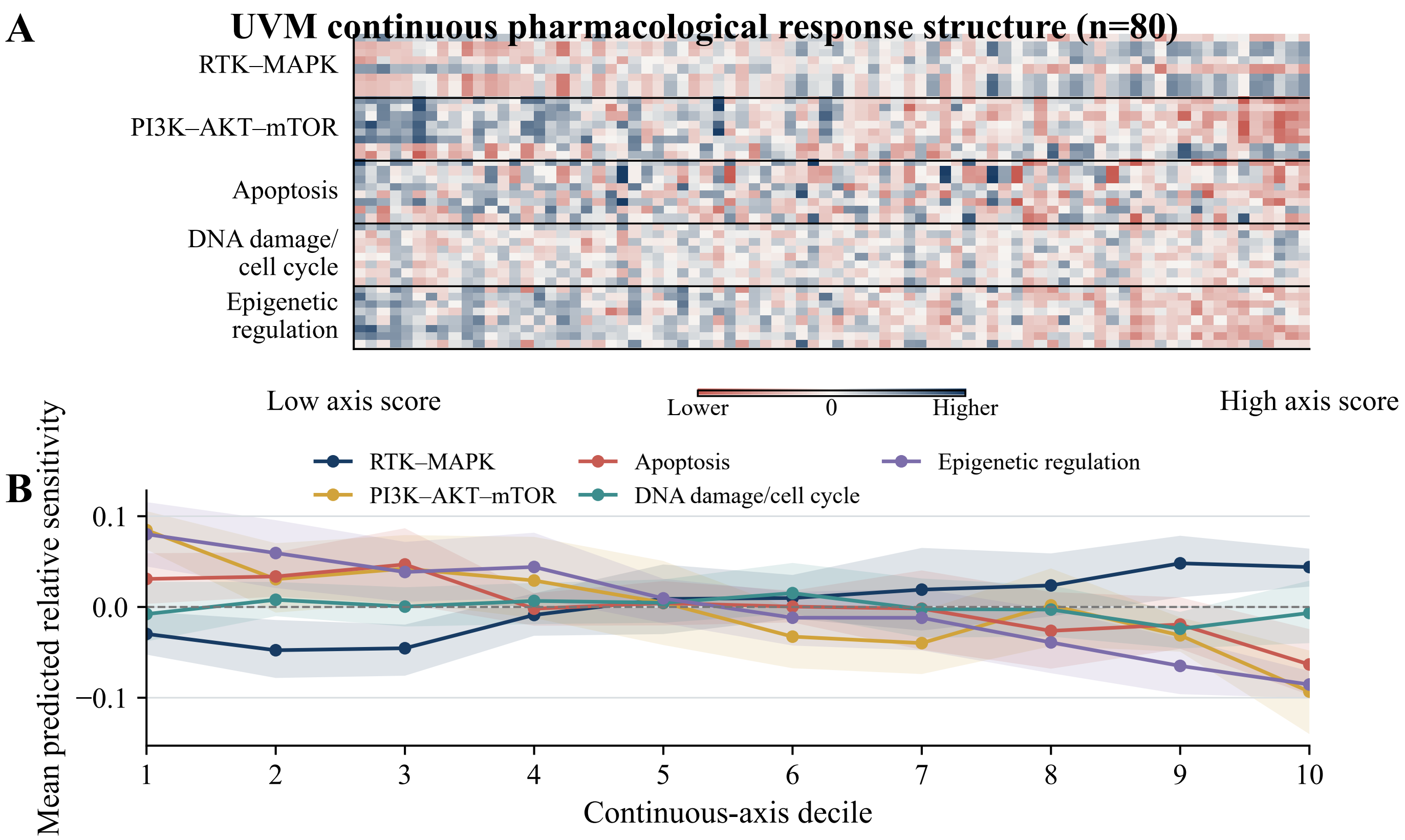

Figure S30 Continuous pharmacological response structure in UVM. A, PRS-construction-excluded-drug response heat map. Patients are ordered by the first principal-component score of the patient-centered 120-drug PRS-construction profile; the 40 displayed PRS-construction-excluded drugs did not contribute to this axis. **B,** Mean predicted relative sensitivity across five PRS-construction-excluded-drug mechanism modules in ten equal-frequency intervals of the continuous axis. Shaded bands denote 95% confidence intervals from 2,000 patient bootstraps. The axis direction is arbitrary and does not denote absolute sensitivity or resistance.

Alt text: Two-panel continuous pharmacological response structure in UVM. Panel A shows predicted responses to 40 PRS-construction-excluded drugs, with patients ordered by the first principal-component score derived from the 120-drug PRS-construction profile. Panel B shows mean predicted relative sensitivity across five drug-mechanism modules along ten equal-frequency intervals of the continuous axis, with 95% bootstrap confidence intervals.

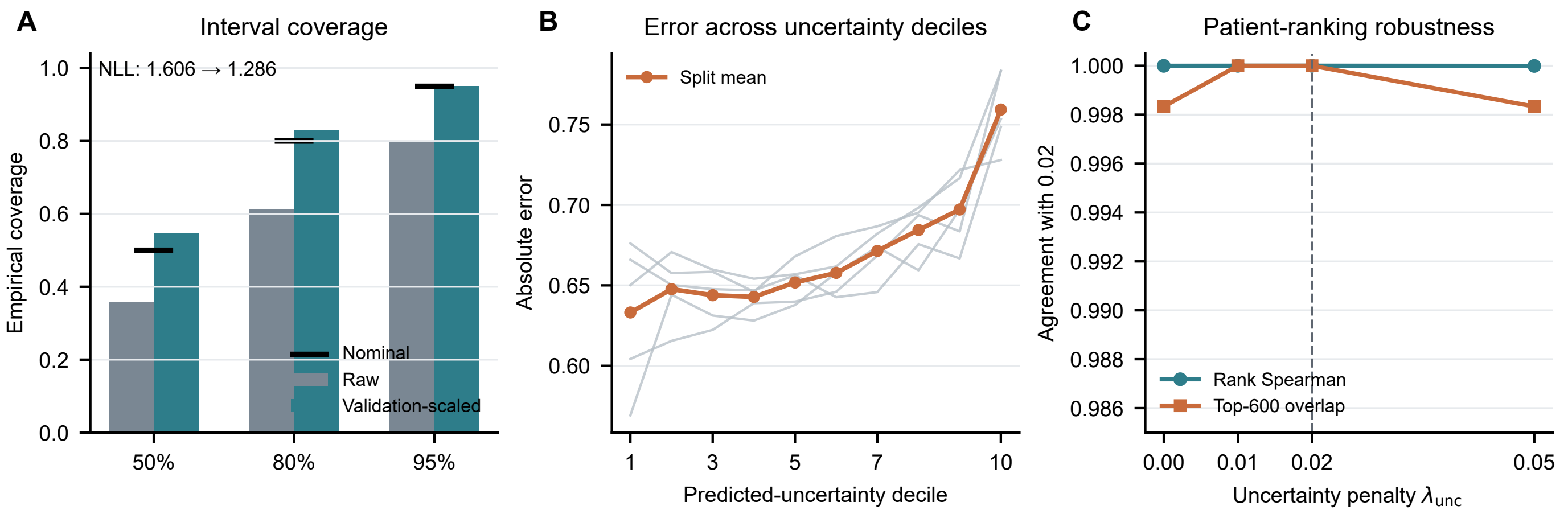

Figure S31 Predictive-uncertainty calibration and patient-ranking robustness. A, Empirical coverage of nominal 50%, 80% and 95% Gaussian intervals across 131,114 held-out cell-line test observations before and after validation-set scalar calibration. Black markers denote nominal coverage. **B,** Absolute standardized-response error across predicted-uncertainty deciles. Gray lines show individual test splits and the orange line shows the split mean. **C,** Median within-cancer rank correlation and top-600 overlap relative to $\lambda_{\text{unc}} = 0.02$ across the evaluated uncertainty penalties.

Alt text: Three-panel evaluation of predictive uncertainty and patient-ranking robustness. Panel A compares empirical and nominal interval coverage before and after validation-set scalar calibration. Panel B shows absolute standardized-response error across predicted-uncertainty deciles for five held-out cell-line test splits. Panel C shows within-cancer rank correlation and top-600 overlap across uncertainty-penalty values relative to $\lambda_{\text{unc}} = 0.02$.
