# Supplementary material for: TRACER: response-protected cross-domain alignment of cancer pharmacogenomic models with patient tumors
Source: Brief Bioinform. 2026 Oct 28;27(5):bbag593. doi: 10.1093/bib/bbag593 (PMC13714793; doi:10.1093/bib/bbag593)
Supplement: Supplementary file 6 [file supplementary_methods_and_tables_bbag593.pdf]

Briefings in Bioinformatics, , pp. 1–27
Supplementary Information

Supplementary Information for: TRACER: Response-Protected Cross-Domain Alignment of Cancer Pharmacogenomic Models with Patient Tumors

Zhenxing Wang,[1] Qihe Wang,[1] Guangchao Wang,[1] Guohua Wang[2,1,*] and Yuming Zhao[1,*]

[1]College of Computer Science and Artificial Intelligence, Northeast Forestry University, Harbin 150040, China
[2]Department of Computer Science and Technology, Faculty of Computing, Harbin Institute of Technology, Harbin 150001, China

[*]Corresponding authors. Guohua Wang, Department of Computer Science and Technology, Faculty of Computing, Harbin Institute of Technology, Harbin 150001, China; College of Computer Science and Artificial Intelligence, Northeast Forestry University, Harbin 150040, China. E-mail: ghwang@nefu.edu.cn; Yuming Zhao, College of Computer Science and Artificial Intelligence, Northeast Forestry University, Harbin 150040, China. E-mail: zym@nefu.edu.cn.

Supplementary Methods

Formal split and training configuration

The five formal data partitions were designated Split 01, Split 02, Split 03, Split 04, and Split 05, with random seeds 2026, 2027, 2031, 2033, and 2035, respectively. Within each partition, cell-line ModelIDs were assigned to training, validation, and test sets in proportions of 70%/15%/15%. The sets were ModelID-disjoint, and every evaluated drug occurred in the training data. All methods used identical pair keys, labels, and source-training per-drug normalization statistics within a split.

TRACER-Full used a maximum training budget of 80 epochs with validation-based early stopping. The response-only stage occupied the first 12 epochs, and alignment was eligible from epoch 13 until training terminated. A stage transition occurred at epoch 25. The drug encoder was frozen after alignment activation, and the drug-prior branch was additionally frozen during epochs 13–24. Early stopping was permitted from epoch 30 with patience 10 and a minimum improvement of 10^{-5}. Checkpoint selection prioritized migration-eligible validation z-MSE and used validation Pearson correlation for tie-breaking. All five splits used the same architecture, loss, optimization, and stopping configuration. The model was implemented in Python with PyTorch. Numerical optimization settings and the frozen training schedule are reported in Table S1.

Exact model architecture

The sample encoder accepted 4,000 expression and 5,000 mutation features. The expression branch used a hidden width of 768, and the fused sample representation had dimension 384. The drug encoder received 14 continuous descriptors, SMILES tokens with vocabulary size 40 and maximum length 96, and up to 48 drug-target and target-pathway relational tokens with 260 node and three relation identifiers, including padding. Drug–disease, drug–indication, approval, treatment, and off-label-use relations were not used for model training or inference. PrimeKG indication relations were used only to curate the downstream indication-recovery evaluation set. The SMILES branch used two Transformer layers. The fused drug representation had dimension 256. Four-head cross-attention produced a 512-dimensional sample–drug interaction representation. The alignment-projection head comprised two transformations and returned a 384-dimensional alignment embedding. Input and model dropout were 0.05 and 0.16. The response head used a hybrid drug prior $p_j = \mu_j + \delta_j$, where $\delta_j = 0.2 \tanh(g_{\text{prior}}(z_j))$, and predicted the standardized residual q_{ij} and z-scale log variance ℓ_{ij}. For response standardization, an invalid or unavailable per-drug source-training standard deviation was replaced by the global source-training response standard deviation, after which the positive floor $\epsilon_\sigma = 10^{-4}$ was applied.

Exact response and alignment objectives

The eight principal response terms were raw mean-squared error, z-scale mean-squared error, raw residual mean-squared error, a combined raw/z Huber loss, z-scale heteroscedastic negative log-likelihood, drug-centered z-residual loss, within-drug ranking, and within-sample ranking. The Huber transition parameters were 1.0 on the raw scale and 0.75 on the z scale, with equal contributions to the combined Huber term. The negative log-likelihood for an observed standardized response y^z_{ij} was

$$\tfrac{1}{2}\exp(-\ell_{ij})(\hat{y}^z_{ij} - y^z_{ij})^2 + \tfrac{1}{2}\ell_{ij},$$

© The Author(s) . Copyright and licence statements to be updated by the publisher during production.

where ℓ_{ij} was clipped to the interval reported in Table S1. Ranking used the group-mean softplus loss

$$\log[1+\exp\{-\operatorname{sign}(y_a^z - y_b^z)(r_a - r_b)\}]\,.$$

Pairs below the target-response separation threshold were excluded. For within-drug ranking, $r=\hat{y}^z$ and pairs were formed among cell lines exposed to the same drug. For within-sample ranking, $r=q$ and pairs were formed among drugs measured in the same ModelID.

Two additional guardrails were active. For a response batch $\mathcal{B}$, let $\mathcal{I}_j = \{n \in \mathcal{B} : d_n = j\}$ and let $\mathcal{J}_{\mathcal{B}} = \{j : |\mathcal{I}_j| \geq n_{\min}\}$ contain drugs meeting the minimum within-batch sample count. Let $\bar{r}_j^{\text{raw}} = |\mathcal{I}_j|^{-1}\sum_{n\in\mathcal{I}_j} r_n^{\text{raw}}$ be the drug-level mean raw sample-specific predicted residual, and let μ_j be the corresponding source-training drug-response mean. After centering both vectors across $\mathcal{J}_{\mathcal{B}}$, define

$$x_j = \bar{r}_j^{\text{raw}} - \frac{1}{|\mathcal{J}_{\mathcal{B}}|}\sum_{q\in\mathcal{J}_{\mathcal{B}}} \bar{r}_q^{\text{raw}}, \qquad u_j = \mu_j - \frac{1}{|\mathcal{J}_{\mathcal{B}}|}\sum_{q\in\mathcal{J}_{\mathcal{B}}} \mu_q.$$

The drug-prior dominance-relief loss is

$$L_{\text{dom}} = \left[\frac{\sum_{j\in\mathcal{J}_{\mathcal{B}}} x_j u_j}{\max(\|x\|_2, \epsilon_{\text{dom}})\max(\|u\|_2, \epsilon_{\text{dom}})}\right]^2.$$

It is set to zero when fewer than three drugs satisfy the eligibility rule. For the standardized sample-specific residual r_n^z, let $\bar{r}_j^z = |\mathcal{I}_j|^{-1}\sum_{n\in\mathcal{I}_j} r_n^z$. The residual-identifiability loss is

$$L_{\text{id}} = \frac{1}{|\mathcal{J}_{\mathcal{B}}|}\sum_{j\in\mathcal{J}_{\mathcal{B}}} (\bar{r}_j^z)^2,$$

and is set to zero when $\mathcal{J}_{\mathcal{B}}$ is empty. The complete guardrail contribution is

$$L_{\text{guard}} = \lambda_{\text{dom}}(t)L_{\text{dom}} + \lambda_{\text{id}}(t)L_{\text{id}},$$

where t is the training epoch and the two nonnegative scheduled weights follow the activation, ramp, and cap settings in Table S1. The implementation used $n_{\min} = 8$ and $\epsilon_{\text{dom}} = 10^{-6}$.

For mutation conditioning, let I_{ik} be the number of shared alterations and B_i^s, B_k^t the mutation burdens. With $B_{\min} = \min(B_i^s, B_k^t)$ and $B_{\max} = \max(B_i^s, B_k^t)$,

$$C_{ik}^{\text{mut}} = \mathbf{1}\{I_{ik} \geq I_{\min}\}\,\operatorname{clip}_{[0,1]}\left[\frac{w_D\, 2I_{ik}/\max(B_i^s + B_k^t, 1) + w_C\, I_{ik}/\max(B_{\min}, 1)}{w_D + w_C}\left(\frac{B_{\min}}{\max(B_{\max}, 1)}\right)^{\eta}\right].$$

C^{cancer} was binary after mapping source lineage and target TCGA cancer codes. C^{path} was the nonnegative cosine similarity between Hallmark activity vectors after L2 normalization with denominator floor $\epsilon_{\text{path}} = 10^{-12}$; a zero vector therefore produced a condition score of zero. Hallmark activity was the mean expression of retained genes in each gene set, standardized for both domains with source-training statistics.

For each source cell line, condition scores determined the eligible target patients and their Top-K ordering. The unconditioned component used an all-one condition matrix, yielding a fixed equal-score subset without embedding-based neighbor ranking. Empty conditional selections contributed zero. All selected edges were aggregated in one condition-weighted ratio with denominator floor ϵ_{NN}. The implemented dispersion contribution is

$$\lambda_{\text{disp}}\frac{1}{d}\sum_{r=1}^{d}\{\max(0, m - s_r^s) + \max(0, m - s_r^t)\},$$

where the effective coefficient λ_{disp} is the sum of the two additive contributions reported in Table S1.

Gradient-regulation algorithm

At each active joint-training step, response and alignment gradients were computed separately. Conflict detection and projection used the flattened gradient over all parameters of the shared sample encoder. If $g_a^\top g_r < 0$, the response component was removed from g_a; otherwise, g_a was retained. Let e denote the global epoch and R_{e-1} the cumulative raw-conflict rate before the current batch. The scheduled target ratio was

$$\rho_e = \rho_{\text{target}}\, w_e\, c(R_{e-1})\, l_e,$$

where w_e is the configured global-epoch ramp, $c(R_{e-1})$ applies high-conflict attenuation, and l_e applies late-stage geometric attenuation from epoch 25. Because alignment began after the configured six-epoch global ramp had elapsed, the first active alignment epoch used the fully ramped value before conflict and late-stage attenuation. The projected auxiliary gradient was rescaled toward ρ_e, constrained by the configured ratio bounds and scale cap, and combined with the unchanged response gradient. The same scaling coefficient was applied to unprojected auxiliary gradients outside the shared sample encoder. Global gradient-norm clipping was applied after gradient combination.

Baseline and ablation definitions

The external baselines were CaDRReS, CODE-AE, GPDRP, HiDRA, and PANCDR. Their implementation, preprocessing, model-selection, hyperparameter, optimization-budget, and TCGA-use details are provided in Supplementary Table S14. Response-only optimized the supervised response objective without alignment. Simple joint optimized response and alignment together without gradient regulation and without the staged migration schedule used by TRACER-Full. Unprotected joint retained the TRACER-Full training schedule but summed the two task gradients without projection. Symmetric surgery projected both task gradients when they conflicted. TRACER-Full projected only the conflicting auxiliary component and preserved the response gradient.

Evaluation and alignment-excluded TCGA audit protocol

Global Pearson and Spearman correlations were calculated over all test pairs after concatenation within a split. z-RMSE was the square root of the mean squared difference between $\hat{y}^z$ and y^z. Means and sample standard deviations were then calculated across the five repeated data partitions, which served as computational evaluation units. Per-drug analysis retained drugs with at least 20 test pairs and finite Pearson correlation in every method and split, producing a common set of 276 drugs.

Within each split, primary-tumor TCGA samples were stratified by cancer type, shuffled with the split seed, and assigned cyclically to five alignment-audit subsets. One subset was excluded from alignment and constituted the alignment-excluded TCGA audit samples; the other four subsets formed the unlabeled alignment pool. The alignment-excluded TCGA audit samples comprised 1,853, 1,848, 1,854, 1,849, and 1,846 patients in Split 01–05, respectively. For Fig. 3, the first five of eight exported source neighbors were averaged within each alignment-excluded TCGA audit sample before aggregation across patients or cancer types. Hallmark neighbor consistency used all Hallmark gene sets; the oncogenic Hallmark subset comprised the 12 oncogenic programs defined for this evaluation.

Gradient diagnostics used valid two-task mini-batches from epochs 13–39. Raw conflict was defined by $\cos(g_r, g_a) < 0$, and meaningful post-projection conflict by a post-rule cosine below -10^{-7}. Projection magnitude was $\|g_a - g_a^{\text{controlled}}\|_2/(\|g_a\|_2 + \epsilon_{\text{grad}})$. Late validation-loss variability was the sample standard deviation divided by the mean of validation z-MSE over epochs 26–39. Mini-batches were descriptive units, while splits were the units for split-level summaries and exact Wilcoxon tests.

Patient ensemble and uncertainty-adjusted response profiles

The five TRACER-Full patient–drug score matrices used identical patient and drug keys and were averaged elementwise. The primary TCGA atlas analysis was transductive because unlabeled target-cohort molecular profiles contributed to cross-domain alignment. Split-specific alignment-excluded TCGA audit samples were excluded from alignment and used for objective-aligned representation diagnostics. Patient sensitivity was represented by

$$s_{kj} = -\hat{y}^z_{kj} - \lambda_{\text{unc}} \exp(\ell_{kj}/2),$$

where $\hat{y}^z_{kj}$ is the z-scale predicted response, $\exp(\ell_{kj}/2)$ is the z-scale predictive standard deviation, and higher s_{kj} denotes greater predicted relative sensitivity. The uncertainty contribution was used as a source-derived ranking penalty.

Predictive-uncertainty calibration and penalty sensitivity

Predictive uncertainty was evaluated on the five ModelID-disjoint held-out cell-line test samples. Within each split, the raw predictive standard deviation $\sigma_i = \exp(\ell_i/2)$ was compared with absolute standardized-response error using Spearman correlation. Gaussian negative log-likelihood and empirical coverage were calculated for central 50%, 80% and 95% normal intervals. A single positive scale factor c_b was fitted on the corresponding source validation set by minimizing Gaussian negative log-likelihood, giving $\sigma_i^{\text{cal}} = c_b\sigma_i$; the fitted factor was then applied unchanged to the test set. Stratified bootstrap resampling within each split generated 95% confidence intervals, and uncertainty deciles were formed separately within each test split.

Patient-ranking sensitivity was evaluated at $\lambda_{\text{unc}} \in \{0, 0.01, 0.02, 0.05\}$. For each alternative coefficient, within-cancer Spearman correlation and top-300, top-600 and top-1,000 overlap were calculated relative to $\lambda_{\text{unc}} = 0.02$. PRS selection was repeated at $\lambda_{\text{unc}} = 0$ for BRCA, UCEC and SARC, and adjusted Rand index and normalized mutual information quantified label agreement at the primary resolutions.

Eligible drugs and PRS-construction separation

A drug was eligible for the partition analysis if it had a unique assignment to one of five mechanism modules, at least 20 source-domain validation pairs in every formal split, a median source-validation per-drug Pearson correlation of at least 0.30 across the five splits, and finite patient-profile variability and cross-split profile-consistency statistics. These criteria yielded 160 drugs. For drug j, the source-validation quality score was

$$q_j = 0.9R_{\text{Pearson}}(r_j^{\text{val}}) + 0.1R_n(n_j^{\text{val}}),$$

where r_j^{val} is its median source-validation per-drug Pearson correlation, n_j^{val} is its minimum source-validation pair count, and the two R terms are percentile ranks among eligible drugs.

Drug pairs were linked when they shared a target, a valid non-acyclic Bemis–Murcko scaffold, or a Morgan-fingerprint Tanimoto similarity of at least 0.70; connected drugs formed a non-overlapping similarity group. Constrained selection assigned each similarity group wholly to one set, maximized the summed source-validation quality score of the excluded set, and selected eight PRS-construction-excluded drugs from

each mechanism module. The resulting 40 PRS-construction-excluded drugs and 120 PRS-construction drugs were used identically across cancer types. Drug eligibility, quality ranking and set construction used source training and validation information exclusively, and source test sets were reserved for response-model evaluation. The 40 excluded drugs were reserved for post-selection characterization and excluded from clustering, selection of k, and within-PRS patient ordering. All 160 drugs were observed during source-domain response-model training.

Uniform within-cancer stability selection

For every cancer type, the ensemble response profile across 120 PRS-construction drugs was centered within patient and evaluated with KMeans for $k = 2, \ldots, 6$. K-means++ initialization, 100 initializations, Euclidean distance and seed 2026 were used throughout. Each candidate required at least 20 patients and 10% of its cancer cohort per group, patient-resampling adjusted Rand index (ARI) of at least 0.80, and proportion of ambiguous clustering (PAC) no greater than 0.20. ARI used 100 repeated 80% patient resamples, whereas PAC was calculated from the corresponding consensus matrix. Threshold sensitivity was evaluated on the common grid of ARI cutoffs 0.80, 0.85 and 0.90 and PAC cutoffs 0.15, 0.20 and 0.25, with all other criteria unchanged.

Joint stability integrated prediction strength, patient-resampling ARI, inverse PAC, silhouette surplus and cross-view ARI. Silhouette surplus was the observed silhouette minus the mean silhouette in matched null datasets at the same k. Cross-view ARI summarized agreement with partitions obtained across individual TRACER models, PRS-construction-drug folds and alternative clustering algorithms. After orienting each metric so that larger values denoted stronger evidence, each was standardized against 1,000 Gaussian-copula unimodal null datasets that preserved the empirical marginal distributions and drug-correlation structure. The joint statistic was the minimum of the five standardized values and therefore reflected the weakest evidence component. Its empirical add-one probability was

$$p_k = \frac{1 + \sum_{b=1}^{B} \mathbf{1}\{J_{kb}^{\text{null}} \geq J_k^{\text{obs}}\}}{B + 1}, \qquad B = 1000.$$

The five candidate probabilities were adjusted within each cancer using the Benjamini–Hochberg procedure. Candidates required a null-model FDR below 0.10, and the largest candidate satisfying every criterion was retained. The multiplicity correction covered the five candidate resolutions evaluated for each cancer type. The resulting discrete PRS structures were interpreted as cancer-specific hypotheses supported by the common stability criteria. If no candidate passed, patient variation was summarized along a continuous pharmacological response axis.

Discrete PRSs and continuous pharmacological response axes

Passing discrete partitions were termed model-derived predicted pharmacological response subtypes (PRSs). After labels were frozen, associations with cancer-specific reference classifications were summarized by Cramér's V, adjusted Rand index, normalized mutual information and 10,000 label permutations. Reference labels comprised PAM50 for BRCA, TCGA molecular classes for UCEC and GDC clinical histological types for SARC; SARC labels were harmonized as DDLPS, LMS, UPS/MFH, MFS, MPNST, SS and Other. Each PRS was compared with all remaining patients using only the 40 PRS-construction-excluded drugs. Module-level mean differences and 95% confidence intervals were obtained using 2,000 patient bootstraps. The excluded drugs were reserved for post-selection pharmacological characterization.

For cancers summarized by a continuous pharmacological response axis, the axis was the first principal-component score of the patient-centered 120-PRS-construction-drug profile. Its arbitrary sign specified patient order without assigning an absolute sensitivity direction. Ten equal-frequency intervals summarized the five PRS-construction-excluded-drug mechanism trajectories using means and 2,000 patient-bootstrap 95% confidence intervals. The excluded-drug values were reserved for visualization and module-level characterization, and the intervals served as descriptive axis bins.

Lineage-aware internal consistency of indication recovery

Known drug–cancer indication recovery ranked all 9,599 patients by ensemble sensitivity for each curated drug. The complete set of 31 eligible mappings was evaluated independently of the 120/40 drug split. At the primary threshold, the 600 highest-ranked patients were compared with all remaining patients using a one-sided Fisher exact test. Benjamini–Hochberg correction was applied across the 31 tests. Forest-plot effect sizes used a Haldane–Anscombe correction of 0.5 in each contingency-table cell and Wald confidence intervals for the log2 odds ratio. Top-300 and top-1,000 analyses assessed threshold sensitivity. Because cancer-type comparability contributed to alignment, indication recovery served as a lineage-aware internal consistency assessment of cancer-type structure retained in the patient rankings.

Supplementary Results

Pan-cancer patient-representation results

The source-validation-defined panel comprised 120 PRS-construction drugs and 40 PRS-construction-excluded drugs. All 33 cancer types were evaluated at (k=2)–6 using 1,000 matched Gaussian-copula null datasets per candidate resolution. Under the common study-defined criteria, BRCA supported (k=4), UCEC supported (k=4), SARC supported (k=2) and COAD supported (k=3); the remaining 29 cancer types were represented by continuous pharmacological response axes (Tables S6–S7). The selected representations were unchanged between 100 and 1,000 null datasets. BRCA, UCEC, SARC and COAD retained the same resolutions when the ARI threshold was increased from 0.80 to 0.85 with PAC fixed at 0.20 (Table S13). Supplementary Figures S1–S30 show the COAD PRSs and the continuous response structures of the remaining cancer types using the revised drug panel.

Predictive-uncertainty calibration and penalty robustness

Across 131,114 held-out cell-line test observations, raw predictive standard deviation showed a Spearman correlation of 0.0506 with absolute error. The raw 50%, 80% and 95% interval coverages were 35.7%, 61.2% and 80.0%. Validation-set scalar calibration increased coverage to 54.5%, 82.8% and 95.1% and reduced Gaussian negative log-likelihood from 1.606 to 1.286 (Supplementary Figure S31A; Table S12). Absolute error generally increased across predicted-uncertainty deciles, with variation among the five test splits (Supplementary Figure S31B).

Patient rankings were stable across $\lambda_{\text{unc}} \in \{0, 0.01, 0.02, 0.05\}$ (Supplementary Figure S31C). At $\lambda_{\text{unc}} = 0$, the median within-cancer Spearman correlation relative to 0.02 was greater than 0.9999 and the minimum top-600 overlap was 0.9917. At the primary resolutions, BRCA, UCEC and SARC yielded adjusted Rand indices of 0.955, 0.983 and 1.000, respectively. The maximum supported resolution changed from $k = 4$ to $k = 5$ in BRCA, while UCEC retained $k = 4$ and SARC retained $k = 2$. The stability across the evaluated range supports retaining $\lambda_{\text{unc}} = 0.02$ as a small ranking regularizer.

Supplementary Tables

Table S1: **Frozen numerical configuration used in the formal TRACER analyses.** The settings correspond to the symbols and procedures defined in the main and Supplementary Methods.

Module	Parameter	Setting
Formal splits	Split labels and random seeds	Split 01: 2026; Split 02: 2027; Split 03: 2031; Split 04: 2033; Split 05: 2035
Input dimensions	Sample and drug inputs	expression 4,000; mutation 5,000; continuous drug descriptors 14
Sample encoder	Hidden and output dimensions	expression hidden width 768; mutation hidden width at least 128; fused sample representation 384
Drug encoder	Branch configuration	drug hidden width 512; fused drug representation 256; SMILES vocabulary 40; two Transformer layers; maximum SMILES length 96; maximum drug-target/target-pathway neighborhood 48; node vocabulary 260; relation vocabulary 3 including padding
Interaction module	Cross-attention	four attention heads; interaction representation 512
Alignment-projection head	Projection architecture	two transformations; alignment embedding 384
Response head	Prior and uncertainty branches	hybrid drug prior; learned correction scale 0.20; heteroscedastic branch enabled
Regularization	Dropout	input dropout 0.05; model dropout 0.16
Training budget	Stages and early stopping	maximum 80 epochs; response-only epochs 1–12; alignment eligible from epoch 13 until training terminated; stage transition at epoch 25; early stopping from epoch 30 with patience 10 and minimum improvement 10^{-5}
Stage freezing	Frozen components	epochs 13–24: drug encoder and drug-prior branch; from epoch 25: drug encoder
Optimizer	AdamW and schedule	initial learning rate 1.2×10^{-4}; minimum learning rate 4×10^{-6}; weight decay 0.012; cosine schedule with three global warm-up epochs; stage learning-rate multipliers 0.35 and 0.12
Batch sizes	Statistical unit per optimizer/evaluation step	768 source response pairs; 512 target alignment patients; 1,536 validation/test pairs; 4,096 patient-inference pairs
Response loss weights	Eight principal terms	$\lambda_{\text{raw}} = 0.08$; $\lambda_z = 0.56$; $\lambda_{\text{res}} = 0.10$; $\lambda_{\text{Huber}} = 0.24$; $\lambda_{\text{NLL}} = 0.002$; $\lambda_{\text{center}} = 0.06$; $\lambda_{\text{drug}} = 0.10$; $\lambda_{\text{sample}} = 0.12$
Robust regression	Huber and log-variance settings	raw Huber transition 1.0; z Huber transition 0.75; equal raw/z Huber contributions; log-variance clip $[-6, 4]$
Response stability	Numerical floors	per-drug response standard deviation $\epsilon_\sigma = 10^{-4}$; weighted-loss denominator 10^{-6}; guardrail cosine floor $\epsilon_{\text{dom}} = 10^{-6}$
Ranking loss	Pair construction	minimum target gap 0.20; maximum 160 ordered pairs per group; random subsampling above the cap; group-mean aggregation
Residual schedule	Principal residual terms	initial residual-focus multiplier 1.00; from epoch 18 residual-focus multiplier 0.85 and response-loss multiplier 1.08
Dominance guardrail	Drug-prior dominance relief	target weight 0.015; start epoch 10; ten-epoch ramp; maximum weight 0.05; minimum eight observations per drug
Identifiability guardrail	Drug-level residual centering	target weight 0.04; start epoch 1; 12-epoch ramp; maximum weight 0.08; minimum eight observations per drug
Mutation condition	Overlap and burden adjustment	$w_D = 0.50$; $w_C = 0.50$; burden exponent $\eta = 0.25$; minimum shared mutations $I_{\min} = 2$
Pathway condition	Hallmark activity	minimum ten retained genes per Hallmark; source-training standardization; nonnegative cosine similarity; L2-normalization floor $\epsilon_{\text{path}} = 10^{-12}$
Conditional pairing	Selection and stability	$K = 8$; conditional threshold $\tau = 0.02$; unconditioned threshold 0; denominator floor $\epsilon_{\text{NN}} = 10^{-6}$
Alignment weights	Neighbor and dispersion objective	$\lambda_{\text{align}} = 0.08$; $\lambda_0 = 0.22$; $\lambda_{\text{mut}} = 0.32$; $\lambda_{\text{cancer}} = 0.28$; $\lambda_{\text{path}} = 0.08$; additive dispersion contributions 0.20 and 0.40, giving effective $\lambda_{\text{disp}} = 0.60$
Dispersion floor	Population standard deviation	$m = 0.12$ for source and target alignment dimensions
Gradient projection	Scope and numerical settings	shared sample encoder; conflict threshold 0; $\epsilon_{\text{grad}} = 10^{-8}$; scale cap $\alpha_{\max} = 25$; global gradient-norm clip 5.0
Auxiliary norm ratio	Scheduled ratio	nominal minimum 0.015; target 0.025; maximum 0.040; high-conflict threshold 0.60; high-conflict multiplier 0.75
Auxiliary attenuation	Global-epoch schedule	six-epoch global ramp parameter, elapsed before alignment activation; geometric multiplier 0.92 from epoch 25; minimum late-stage multiplier 0.40

Continued on next page

Table S1 (continued)

Module	Parameter	Setting
Checkpoint selection	Validation criterion	migration-eligible validation z-MSE prioritized; validation Pearson used for tie-breaking; best eligible checkpoint restored
Patient scoring	Main profile score	$s = -\hat{y}^z - 0.02\exp(\ell/2)$; standard-deviation penalty on the z scale; higher score indicates greater predicted relative sensitivity

Note: All numerical settings were fixed for formal evaluation and applied identically across the five splits without split-specific retuning. The two additive dispersion contributions call the same implemented dispersion-floor function and therefore yield the effective coefficient shown above.

Alt text: Table S1. Frozen settings for the model architecture, response objective, biologically conditioned neighbor alignment, gradient regulation and formal evaluation. Each row identifies a component, parameter and setting used across the five data splits.

Table S2: **Split-level drug-response prediction performance of TRACER training strategies.** Metrics were computed on identical ModelID-disjoint, seen-drug test pairs.

Method	Data split	Test pairs	Pearson	Spearman	z-RMSE
Response-only	Split 01 (seed 2026)	26,168	0.5145	0.4893	0.8431
Response-only	Split 02 (seed 2027)	26,479	0.5180	0.4959	0.8737
Response-only	Split 03 (seed 2031)	25,737	0.5133	0.4860	0.8321
Response-only	Split 04 (seed 2033)	26,059	0.5486	0.5449	0.8781
Response-only	Split 05 (seed 2035)	26,671	0.4951	0.4823	0.8394
Response-only	Mean ± SD	–	0.5179 ± 0.0193	0.4997 ± 0.0258	0.8533 ± 0.0211
Simple joint	Split 01 (seed 2026)	26,168	0.4148	0.4115	0.9278
Simple joint	Split 02 (seed 2027)	26,479	0.4440	0.4355	0.9219
Simple joint	Split 03 (seed 2031)	25,737	0.4590	0.4516	0.8794
Simple joint	Split 04 (seed 2033)	26,059	0.4770	0.4655	0.9299
Simple joint	Split 05 (seed 2035)	26,671	0.4676	0.4609	0.8633
Simple joint	Mean ± SD	–	0.4525 ± 0.0243	0.4450 ± 0.0220	0.9045 ± 0.0309
Unprotected joint	Split 01 (seed 2026)	26,168	0.4978	0.4820	0.8547
Unprotected joint	Split 02 (seed 2027)	26,479	0.4915	0.4740	0.8937
Unprotected joint	Split 03 (seed 2031)	25,737	0.5032	0.4877	0.8438
Unprotected joint	Split 04 (seed 2033)	26,059	0.5345	0.5241	0.8879
Unprotected joint	Split 05 (seed 2035)	26,671	0.4873	0.4811	0.8511
Unprotected joint	Mean ± SD	–	0.5028 ± 0.0187	0.4898 ± 0.0198	0.8662 ± 0.0229
TRACER-Full	Split 01 (seed 2026)	26,168	0.5058	0.4878	0.8521
TRACER-Full	Split 02 (seed 2027)	26,479	0.5159	0.4941	0.8774
TRACER-Full	Split 03 (seed 2031)	25,737	0.5037	0.4867	0.8476
TRACER-Full	Split 04 (seed 2033)	26,059	0.5437	0.5340	0.8847
TRACER-Full	Split 05 (seed 2035)	26,671	0.4933	0.4856	0.8482
TRACER-Full	Mean ± SD	–	0.5125 ± 0.0192	0.4976 ± 0.0206	0.8620 ± 0.0177

Note: The summary rows report the arithmetic mean and sample standard deviation across the five formal data splits.

Alt text: Table S2. Split-level drug-response prediction metrics for the TRACER training strategies on identical held-out cell-line test samples. Rows list each strategy and split, followed by five-split summaries; columns report test-pair counts, Pearson and Spearman correlations, and standardized root-mean-square prediction error.

Table S3: **Split-level drug-response prediction performance of external baseline methods.** Metrics were computed on identical ModelID-disjoint, seen-drug test pairs.

Method	Data split	Test pairs	Pearson	Spearman	z-RMSE
CaDRReS	Split 01 (seed 2026)	26,168	0.4983	0.4854	0.9221
CaDRReS	Split 02 (seed 2027)	26,479	0.5159	0.4873	0.9189
CaDRReS	Split 03 (seed 2031)	25,737	0.4331	0.4159	0.8983
CaDRReS	Split 04 (seed 2033)	26,059	0.5194	0.5242	0.9261
CaDRReS	Split 05 (seed 2035)	26,671	0.4743	0.4665	0.8620
CaDRReS	Mean ± SD	–	0.4882 ± 0.0356	0.4759 ± 0.0395	0.9055 ± 0.0266
CODE-AE	Split 01 (seed 2026)	26,168	0.4754	0.4562	0.8631
CODE-AE	Split 02 (seed 2027)	26,479	0.4915	0.4732	0.8915
CODE-AE	Split 03 (seed 2031)	25,737	0.4862	0.4695	0.8447
CODE-AE	Split 04 (seed 2033)	26,059	0.5270	0.5248	0.8932

Continued on next page

Table S3 (continued)

Method	Data split	Test pairs	Pearson	Spearman	z-RMSE
CODE-AE	Split 05 (seed 2035)	26,671	0.4765	0.4625	0.8414
CODE-AE	Mean ± SD	–	0.4913 ± 0.0210	0.4772 ± 0.0274	0.8668 ± 0.0248
GPDRP	Split 01 (seed 2026)	26,168	0.4275	0.3759	0.8838
GPDRP	Split 02 (seed 2027)	26,479	0.4375	0.3844	0.9184
GPDRP	Split 03 (seed 2031)	25,737	0.4312	0.3853	0.8801
GPDRP	Split 04 (seed 2033)	26,059	0.4783	0.4423	0.9231
GPDRP	Split 05 (seed 2035)	26,671	0.4096	0.3598	0.8707
GPDRP	Mean ± SD	–	0.4368 ± 0.0254	0.3895 ± 0.0312	0.8952 ± 0.0238
HiDRA	Split 01 (seed 2026)	26,168	0.3233	0.3587	1.0368
HiDRA	Split 02 (seed 2027)	26,479	0.4244	0.3847	0.9514
HiDRA	Split 03 (seed 2031)	25,737	0.4199	0.4049	0.9052
HiDRA	Split 04 (seed 2033)	26,059	0.4365	0.4514	0.9724
HiDRA	Split 05 (seed 2035)	26,671	0.3004	0.2864	0.9584
HiDRA	Mean ± SD	–	0.3809 ± 0.0638	0.3772 ± 0.0611	0.9648 ± 0.0475
PANCDR	Split 01 (seed 2026)	26,168	0.4628	0.4371	0.8698
PANCDR	Split 02 (seed 2027)	26,479	0.5228	0.4780	0.8724
PANCDR	Split 03 (seed 2031)	25,737	0.4771	0.4698	0.8454
PANCDR	Split 04 (seed 2033)	26,059	0.5311	0.5230	0.8899
PANCDR	Split 05 (seed 2035)	26,671	0.4514	0.4428	0.8550
PANCDR	Mean ± SD	–	0.4890 ± 0.0359	0.4701 ± 0.0343	0.8665 ± 0.0171

Note: The summary rows report the arithmetic mean and sample standard deviation across the five formal data splits.

Alt text: Table S3. Split-level drug-response prediction metrics for the external baselines evaluated on the same held-out cell-line test samples. Rows identify methods and splits, with five-split summaries; columns report test-pair counts, Pearson and Spearman correlations, and standardized root-mean-square prediction error.

Table S4: **Cancer-resolved gain in oncogenic Hallmark neighbor consistency relative to Response-only.** Values correspond to Fig. 3A and use Top-5 neighbor pathway cosine similarity.

Cancer	Mean audit-sample n	Simple joint	Unprotected joint	TRACER-Full	Full highest
ACC	16	-0.1883 ± 0.1111 (0/5)	-0.1602 ± 0.0999 (0/5)	-0.0649 ± 0.0398 (0/5)	Yes
BLCA	81	-0.1522 ± 0.1313 (0/5)	-0.1576 ± 0.1172 (1/5)	0.0024 ± 0.0736 (4/5)	Yes
BRCA	193	0.2583 ± 0.0993 (5/5)	0.1896 ± 0.2025 (4/5)	0.3923 ± 0.0988 (5/5)	Yes
CESC	57	-0.1722 ± 0.1005 (0/5)	-0.2038 ± 0.0776 (0/5)	-0.0732 ± 0.0811 (1/5)	Yes
CHOL	7	-0.0315 ± 0.0630 (3/5)	-0.1459 ± 0.1998 (2/5)	0.0919 ± 0.0408 (5/5)	Yes
COAD	86	0.1302 ± 0.1536 (3/5)	0.0866 ± 0.0680 (5/5)	0.2662 ± 0.1499 (5/5)	Yes
DLBC	10	0.3697 ± 0.1620 (5/5)	0.3669 ± 0.2405 (5/5)	0.4615 ± 0.1687 (5/5)	Yes
ESCA	37	-0.1060 ± 0.1313 (1/5)	-0.1766 ± 0.1210 (0/5)	0.0191 ± 0.0620 (4/5)	Yes
GBM	49	0.1033 ± 0.1589 (4/5)	0.0452 ± 0.2592 (3/5)	0.2446 ± 0.1715 (5/5)	Yes
HNSC	100	-0.2134 ± 0.1282 (0/5)	-0.2252 ± 0.1028 (0/5)	-0.0844 ± 0.0451 (0/5)	Yes
KICH	14	-0.1259 ± 0.1419 (1/5)	-0.0739 ± 0.0996 (1/5)	-0.0083 ± 0.0795 (3/5)	Yes
KIRC	75	-0.1109 ± 0.0867 (0/5)	-0.1945 ± 0.1075 (0/5)	0.0012 ± 0.0585 (2/5)	Yes
KIRP	56	-0.1507 ± 0.0534 (0/5)	-0.1667 ± 0.1083 (1/5)	-0.0110 ± 0.0656 (2/5)	Yes
LGG	102	0.2487 ± 0.0959 (5/5)	0.2542 ± 0.2166 (5/5)	0.3578 ± 0.1193 (5/5)	Yes
LIHC	73	-0.1139 ± 0.1444 (2/5)	-0.1234 ± 0.1129 (0/5)	-0.0018 ± 0.0898 (4/5)	Yes
LUAD	101	-0.1345 ± 0.1064 (0/5)	-0.1446 ± 0.0996 (0/5)	0.0444 ± 0.0691 (4/5)	Yes
LUSC	97	-0.1949 ± 0.1433 (0/5)	-0.2256 ± 0.1257 (0/5)	-0.0504 ± 0.0773 (1/5)	Yes
MESO	16	-0.1312 ± 0.0474 (0/5)	-0.2439 ± 0.1574 (1/5)	-0.0433 ± 0.0776 (2/5)	Yes
OV	57	-0.1606 ± 0.0840 (0/5)	-0.1995 ± 0.1010 (0/5)	-0.0360 ± 0.0554 (1/5)	Yes
PAAD	33	-0.1439 ± 0.1701 (0/5)	-0.1724 ± 0.1133 (0/5)	0.0323 ± 0.1547 (3/5)	Yes
PCPG	36	0.1169 ± 0.1159 (3/5)	0.1770 ± 0.1666 (5/5)	0.2541 ± 0.1519 (5/5)	Yes
PRAD	98	0.1212 ± 0.1192 (4/5)	0.1271 ± 0.1722 (4/5)	0.2440 ± 0.0647 (5/5)	Yes
READ	31	0.1217 ± 0.1682 (4/5)	0.0488 ± 0.1108 (4/5)	0.2367 ± 0.1495 (5/5)	Yes
SARC	47	-0.1565 ± 0.1321 (0/5)	-0.2379 ± 0.1222 (0/5)	-0.0044 ± 0.0724 (1/5)	Yes
SKCM	22	0.0316 ± 0.0746 (4/5)	0.0278 ± 0.1289 (3/5)	0.2037 ± 0.1099 (5/5)	Yes
STAD	77	-0.0132 ± 0.1315 (3/5)	-0.0735 ± 0.1065 (2/5)	0.1307 ± 0.0601 (5/5)	Yes
TGCT	28	0.2528 ± 0.0981 (5/5)	0.1409 ± 0.1828 (4/5)	0.2841 ± 0.0908 (5/5)	Yes
THCA	97	-0.1911 ± 0.0636 (0/5)	-0.1961 ± 0.1047 (0/5)	-0.0501 ± 0.1012 (3/5)	Yes
THYM	24	0.3271 ± 0.1274 (5/5)	0.2705 ± 0.1636 (5/5)	0.3522 ± 0.0982 (5/5)	Yes
UCEC	102	-0.0607 ± 0.0621 (1/5)	-0.0799 ± 0.1342 (1/5)	0.0961 ± 0.0542 (5/5)	Yes
UCS	12	-0.0296 ± 0.0948 (2/5)	-0.1174 ± 0.0943 (1/5)	0.1050 ± 0.1067 (5/5)	Yes
UVM	16	-0.0349 ± 0.2517 (2/5)	-0.0382 ± 0.1553 (1/5)	0.0431 ± 0.1535 (4/5)	Yes

Note: Each strategy cell reports mean ± SD across five splits, followed by the number of splits with a positive gain in parentheses.

Alt text: Table S4. Cancer-specific changes in oncogenic Hallmark neighbor consistency relative to Response-only. Rows identify cancer types, and strategy columns report five-split means, standard deviations and the number of splits with a positive change.

Table S5: **Split-level gradient-conflict, intervention, and training-outcome diagnostics.** Values correspond to Fig. 4.

Strategy	Data split	Raw conflict	Post conflict	Projection	Aux/response	Response-gradient modification	Pearson loss	Validation-loss CV
Simple joint	Split 01 (seed 2026)	73.07%	73.07%	0.00%	3.64%	0.00%	19.38%	2.34%
Simple joint	Split 02 (seed 2027)	71.63%	71.63%	0.00%	3.70%	0.00%	14.29%	2.33%
Simple joint	Split 03 (seed 2031)	73.12%	73.12%	0.00%	3.79%	0.00%	10.58%	2.49%
Simple joint	Split 04 (seed 2033)	72.22%	72.22%	0.00%	3.58%	0.00%	13.05%	3.24%
Simple joint	Split 05 (seed 2035)	70.91%	70.91%	0.00%	3.95%	0.00%	5.55%	2.91%
Simple joint	Mean ± SD	72.19 ± 0.95%	72.19 ± 0.95%	0.00 ± 0.00%	3.73 ± 0.14%	0.00 ± 0.00%	12.57 ± 5.07%	2.66 ± 0.40%
Unprotected joint	Split 01 (seed 2026)	68.58%	68.58%	0.00%	3.13%	0.00%	3.26%	0.89%
Unprotected joint	Split 02 (seed 2027)	72.47%	72.47%	0.00%	3.47%	0.00%	5.11%	0.66%
Unprotected joint	Split 03 (seed 2031)	71.76%	71.76%	0.00%	3.42%	0.00%	1.97%	0.79%
Unprotected joint	Split 04 (seed 2033)	65.30%	65.30%	0.00%	2.83%	0.00%	2.58%	0.98%
Unprotected joint	Split 05 (seed 2035)	74.26%	74.26%	0.00%	3.63%	0.00%	1.59%	0.83%
Unprotected joint	Mean ± SD	70.47 ± 3.55%	70.47 ± 3.55%	0.00 ± 0.00%	3.30 ± 0.31%	0.00 ± 0.00%	2.90 ± 1.39%	0.83 ± 0.12%
Symmetric surgery	Split 01 (seed 2026)	71.95%	0.00%	4.44%	0.88%	4.44%	1.87%	0.88%
Symmetric surgery	Split 02 (seed 2027)	77.85%	0.00%	4.81%	0.97%	4.81%	3.33%	0.41%
Symmetric surgery	Split 03 (seed 2031)	75.97%	0.00%	4.74%	0.96%	4.74%	0.70%	0.51%
Symmetric surgery	Split 04 (seed 2033)	67.69%	0.00%	4.16%	0.75%	4.16%	1.57%	0.87%
Symmetric surgery	Split 05 (seed 2035)	78.34%	0.00%	4.94%	1.01%	4.94%	0.88%	0.50%
Symmetric surgery	Mean ± SD	74.36 ± 4.50%	0.00 ± 0.00%	4.62 ± 0.31%	0.91 ± 0.10%	4.62 ± 0.31%	1.67 ± 1.05%	0.63 ± 0.22%
TRACER-Full	Split 01 (seed 2026)	61.47%	0.00%	2.25%	1.43%	0.00%	1.69%	0.74%
TRACER-Full	Split 02 (seed 2027)	63.27%	0.00%	2.12%	1.39%	0.00%	0.41%	0.82%
TRACER-Full	Split 03 (seed 2031)	62.38%	0.00%	2.14%	1.38%	0.00%	1.87%	0.88%
TRACER-Full	Split 04 (seed 2033)	59.79%	0.00%	2.38%	1.54%	0.00%	0.89%	1.08%
TRACER-Full	Split 05 (seed 2035)	63.27%	0.00%	2.10%	1.43%	0.00%	0.37%	1.05%
TRACER-Full	Mean ± SD	62.04 ± 1.46%	0.00 ± 0.00%	2.20 ± 0.12%	1.43 ± 0.06%	0.00 ± 0.00%	1.05 ± 0.71%	0.91 ± 0.15%

Note: Gradient statistics use valid two-task batches from epochs 13–39. Meaningful post-rule conflict is defined using a numerical tolerance of -10^{-7}. The split is the statistical summary unit, and mini-batches are descriptive units.

Alt text: Table S5. Split-level gradient-conflict, intervention and training-outcome diagnostics for four joint-training strategies. Columns describe conflict before and after gradient regulation, projection effects and response-prediction outcomes, with summaries across the five formal splits.

Table S6: **Candidate-resolution metrics for all 33 cancer types.** Each cancer was evaluated at $k = 2$–6 using 1,000 matched Gaussian-copula null datasets. Pass denotes minimum group size ≥ 20, minimum group fraction $\geq 10\%$, ARI ≥ 0.80, PAC ≤ 0.20 and within-cancer BH-FDR < 0.10.

Cancer	k	n	Min. n	Min. frac.	Pred. strength	ARI	PAC	Sil. surplus	Cross-view ARI	Joint z	BH-FDR	Pass
ACC	2	77	35	0.455	0.623	0.870	0.242	0.0000	0.554	0.002	0.494	No
ACC	3	77	20	0.260	0.223	0.640	0.523	0.0064	0.363	-0.680	0.494	No
ACC	4	77	7	0.091	0.045	0.498	0.514	0.0052	0.361	-0.651	0.494	No
ACC	5	77	7	0.091	0.000	0.514	0.476	0.0126	0.376	-0.641	0.494	No
ACC	6	77	7	0.091	0.000	0.459	0.434	0.0102	0.335	-0.554	0.494	No
BLCA	2	401	186	0.464	0.788	0.919	0.112	0.0080	0.563	0.121	0.169	No
BLCA	3	401	97	0.242	0.461	0.733	0.378	0.0059	0.472	0.141	0.161	No
BLCA	4	401	83	0.207	0.380	0.735	0.311	0.0079	0.446	0.886	0.115	No
BLCA	5	401	61	0.152	0.301	0.571	0.346	0.0072	0.342	0.166	0.161	No
BLCA	6	401	60	0.150	0.257	0.504	0.334	0.0045	0.333	0.333	0.155	No
BRCA	2	964	443	0.460	0.764	0.903	0.163	0.0038	0.539	-1.107	0.680	No
BRCA	3	964	256	0.266	0.526	0.790	0.366	0.0050	0.430	-0.628	0.541	No
BRCA	4	964	197	0.204	0.497	0.869	0.167	0.0046	0.408	1.028	0.057	Yes
BRCA	5	964	164	0.170	0.360	0.797	0.188	0.0053	0.415	1.058	0.057	No
BRCA	6	964	115	0.119	0.279	0.615	0.292	0.0072	0.325	-0.017	0.193	No
CESC	2	284	138	0.486	0.800	0.961	0.063	0.0066	0.623	0.722	0.105	No
CESC	3	284	69	0.243	0.515	0.810	0.239	0.0105	0.472	0.845	0.105	No
CESC	4	284	62	0.218	0.371	0.695	0.368	0.0020	0.432	0.130	0.219	No
CESC	5	284	45	0.158	0.267	0.582	0.377	-0.0022	0.360	-0.416	0.299	No
CESC	6	284	30	0.106	0.209	0.490	0.366	-0.0002	0.315	-0.106	0.219	No
CHOL	2	35	11	0.314	0.371	0.788	0.363	0.0064	0.522	-0.005	0.905	No
CHOL	3	35	6	0.171	0.017	0.506	0.667	0.0121	0.338	-0.843	0.905	No
CHOL	4	35	4	0.114	0.000	0.396	0.645	-0.0121	0.371	-1.505	0.905	No
CHOL	5	35	3	0.086	0.000	0.399	0.593	0.0108	0.263	-1.769	0.905	No
CHOL	6	35	3	0.086	0.000	0.404	0.494	0.0047	0.308	-1.225	0.905	No
COAD	2	427	178	0.417	0.758	0.920	0.152	0.0128	0.580	-0.482	0.404	No
COAD	3	427	117	0.274	0.596	0.879	0.150	0.0162	0.503	1.087	0.055	Yes
COAD	4	427	91	0.213	0.398	0.800	0.248	0.0186	0.501	0.397	0.136	No
COAD	5	427	65	0.152	0.304	0.617	0.327	0.0195	0.399	0.126	0.136	No
COAD	6	427	56	0.131	0.259	0.584	0.313	0.0212	0.396	0.315	0.136	No
DLBC	2	47	21	0.447	0.564	0.875	0.270	0.0070	0.583	0.302	0.629	No
DLBC	3	47	8	0.170	0.047	0.563	0.491	0.0219	0.380	-0.601	0.672	No
DLBC	4	47	8	0.170	0.000	0.486	0.528	-0.0034	0.377	-0.492	0.672	No
DLBC	5	47	5	0.106	0.000	0.516	0.493	0.0019	0.361	-0.702	0.672	No
DLBC	6	47	5	0.106	0.000	0.381	0.461	-0.0208	0.281	-1.506	0.820	No
ESCA	2	182	88	0.484	0.555	0.650	0.545	-0.0061	0.402	-1.175	0.725	No
ESCA	3	182	46	0.253	0.387	0.520	0.588	0.0010	0.343	-1.158	0.725	No
ESCA	4	182	42	0.231	0.309	0.637	0.390	0.0105	0.458	0.789	0.060	No
ESCA	5	182	32	0.176	0.224	0.576	0.364	0.0059	0.360	0.989	0.045	No
ESCA	6	182	28	0.154	0.126	0.508	0.353	0.0038	0.401	0.617	0.060	No
GBM	2	245	119	0.486	0.730	0.916	0.120	0.0016	0.549	0.169	0.746	No
GBM	3	245	70	0.286	0.413	0.509	0.550	-0.0016	0.329	-1.346	0.746	No
GBM	4	245	52	0.212	0.318	0.541	0.482	-0.0042	0.354	-1.051	0.746	No
GBM	5	245	42	0.171	0.232	0.457	0.433	-0.0073	0.316	-1.379	0.746	No
GBM	6	245	30	0.122	0.183	0.442	0.393	-0.0037	0.332	-0.906	0.746	No
HNSC	2	500	242	0.484	0.773	0.928	0.109	0.0021	0.519	0.329	0.265	No
HNSC	3	500	152	0.304	0.463	0.730	0.313	0.0018	0.361	0.133	0.265	No
HNSC	4	500	100	0.200	0.364	0.573	0.377	0.0023	0.332	0.007	0.265	No

Continued on next page

Table S6 (continued)

Cancer	k	n	Min. n	Min. frac.	Pred. strength	ARI	PAC	Sil. surplus	Cross-view ARI	Joint z	BH-FDR	Pass
HNSC	5	500	93	0.186	0.306	0.612	0.336	-0.0012	0.325	-0.366	0.336	No
HNSC	6	500	76	0.152	0.248	0.448	0.369	-0.0047	0.271	-1.428	0.776	No
KICH	2	66	32	0.485	0.601	0.833	0.297	-0.0058	0.544	-0.311	0.442	No
KICH	3	66	13	0.197	0.279	0.675	0.373	0.0056	0.423	0.407	0.375	No
KICH	4	66	12	0.182	0.026	0.584	0.477	-0.0009	0.394	-0.390	0.442	No
KICH	5	66	10	0.152	0.000	0.494	0.472	-0.0062	0.322	-0.601	0.442	No
KICH	6	66	3	0.045	0.000	0.427	0.419	0.0052	0.325	-0.242	0.442	No
KIRC	2	372	181	0.487	0.705	0.889	0.184	0.0006	0.463	0.091	0.728	No
KIRC	3	372	112	0.301	0.461	0.610	0.479	-0.0057	0.353	-1.221	0.728	No
KIRC	4	372	85	0.228	0.348	0.522	0.480	-0.0036	0.275	-1.056	0.728	No
KIRC	5	372	62	0.167	0.279	0.461	0.455	-0.0050	0.266	-1.385	0.728	No
KIRC	6	372	53	0.142	0.225	0.418	0.417	-0.0009	0.272	-1.371	0.728	No
KIRP	2	277	117	0.422	0.772	0.948	0.085	0.0074	0.557	0.744	0.185	No
KIRP	3	277	85	0.307	0.427	0.555	0.527	-0.0118	0.322	-1.795	0.910	No
KIRP	4	277	56	0.202	0.324	0.382	0.513	-0.0033	0.240	-1.843	0.910	No
KIRP	5	277	43	0.155	0.251	0.496	0.440	-0.0003	0.323	-0.961	0.910	No
KIRP	6	277	36	0.130	0.185	0.449	0.396	-0.0011	0.282	-0.667	0.910	No
LAML	2	59	21	0.356	0.481	0.670	0.512	-0.0004	0.410	-0.575	0.434	No
LAML	3	59	16	0.271	0.209	0.704	0.475	0.0061	0.512	0.218	0.328	No
LAML	4	59	10	0.169	0.017	0.584	0.454	0.0013	0.461	-0.242	0.328	No
LAML	5	59	4	0.068	0.000	0.554	0.441	0.0196	0.445	-0.093	0.328	No
LAML	6	59	3	0.051	0.000	0.508	0.418	0.0127	0.380	-0.056	0.328	No
LGG	2	507	203	0.400	0.789	0.938	0.092	0.0062	0.531	0.319	0.433	No
LGG	3	507	137	0.270	0.455	0.637	0.373	-0.0004	0.369	-0.314	0.433	No
LGG	4	507	112	0.221	0.359	0.550	0.443	-0.0021	0.267	-0.741	0.547	No
LGG	5	507	87	0.172	0.309	0.524	0.411	-0.0050	0.315	-1.342	0.734	No
LGG	6	507	69	0.136	0.262	0.597	0.329	-0.0013	0.350	-0.389	0.433	No
LIHC	2	362	171	0.472	0.764	0.921	0.138	0.0007	0.543	-0.065	0.946	No
LIHC	3	362	111	0.307	0.424	0.580	0.528	-0.0074	0.327	-1.366	0.946	No
LIHC	4	362	83	0.229	0.325	0.458	0.520	-0.0085	0.296	-2.058	0.946	No
LIHC	5	362	57	0.157	0.279	0.562	0.411	-0.0008	0.345	-0.724	0.946	No
LIHC	6	362	53	0.146	0.232	0.430	0.396	-0.0057	0.278	-1.496	0.946	No
LUAD	2	505	222	0.440	0.803	0.926	0.090	0.0057	0.580	-0.392	0.411	No
LUAD	3	505	145	0.287	0.506	0.771	0.311	0.0102	0.416	0.389	0.348	No
LUAD	4	505	122	0.242	0.365	0.630	0.368	-0.0013	0.391	-0.798	0.502	No
LUAD	5	505	87	0.172	0.301	0.584	0.367	0.0021	0.353	-0.278	0.348	No
LUAD	6	505	58	0.115	0.264	0.486	0.351	0.0005	0.304	-0.095	0.348	No
LUSC	2	482	224	0.465	0.733	0.908	0.151	-0.0034	0.521	-0.814	0.531	No
LUSC	3	482	146	0.303	0.440	0.666	0.473	-0.0017	0.375	-0.783	0.531	No
LUSC	4	482	92	0.191	0.376	0.756	0.257	0.0017	0.401	0.335	0.470	No
LUSC	5	482	79	0.164	0.295	0.540	0.385	0.0025	0.329	-0.388	0.470	No
LUSC	6	482	53	0.110	0.243	0.518	0.364	0.0005	0.286	-0.252	0.470	No
MESO	2	78	29	0.372	0.508	0.694	0.531	-0.0103	0.407	-0.823	0.544	No
MESO	3	78	22	0.282	0.277	0.508	0.609	-0.0035	0.421	-0.546	0.456	No
MESO	4	78	14	0.179	0.091	0.448	0.523	0.0052	0.317	0.151	0.260	No
MESO	5	78	11	0.141	0.011	0.428	0.502	0.0015	0.293	-0.315	0.260	No
MESO	6	78	6	0.077	0.000	0.407	0.451	0.0057	0.266	-0.101	0.408	No
OV	2	283	125	0.442	0.690	0.854	0.201	-0.0026	0.473	-0.337	0.765	No
OV	3	283	87	0.307	0.497	0.796	0.259	0.0038	0.432	0.703	0.290	No
OV	4	283	67	0.237	0.330	0.498	0.486	-0.0048	0.317	-1.084	0.765	No
OV	5	283	48	0.170	0.240	0.420	0.469	-0.0064	0.279	-1.537	0.802	No

Continued on next page

Table S6 (continued)

Cancer	k	n	Min. n	Min. frac.	Pred. strength	ARI	PAC	Sil. surplus	Cross-view ARI	Joint z	BH-FDR	Pass
OV	6	283	32	0.113	0.183	0.413	0.417	0.0014	0.255	-1.018	0.765	No
PAAD	2	162	77	0.475	0.641	0.881	0.242	-0.0018	0.543	-0.785	0.572	No
PAAD	3	162	34	0.210	0.414	0.586	0.548	-0.0042	0.373	-0.990	0.572	No
PAAD	4	162	29	0.179	0.271	0.529	0.492	-0.0041	0.334	-0.639	0.572	No
PAAD	5	162	24	0.148	0.164	0.482	0.470	0.0031	0.345	-0.978	0.572	No
PAAD	6	162	10	0.062	0.053	0.427	0.429	0.0030	0.316	-1.013	0.572	No
PCPG	2	177	86	0.486	0.674	0.885	0.220	0.0039	0.519	-0.290	0.370	No
PCPG	3	177	38	0.215	0.424	0.681	0.446	-0.0002	0.437	-0.337	0.370	No
PCPG	4	177	34	0.192	0.314	0.543	0.463	-0.0005	0.375	-0.590	0.370	No
PCPG	5	177	29	0.164	0.208	0.523	0.403	0.0004	0.381	0.068	0.300	No
PCPG	6	177	22	0.124	0.119	0.453	0.384	0.0010	0.311	0.052	0.300	No
PRAD	2	488	240	0.492	0.625	0.798	0.337	-0.0029	0.358	-0.551	0.465	No
PRAD	3	488	141	0.289	0.498	0.845	0.250	0.0004	0.417	-0.170	0.332	No
PRAD	4	488	112	0.230	0.371	0.621	0.385	0.0031	0.341	-0.178	0.330	No
PRAD	5	488	89	0.182	0.317	0.670	0.305	0.0023	0.361	0.396	0.147	No
PRAD	6	488	68	0.139	0.264	0.578	0.327	0.0030	0.335	0.376	0.147	No
READ	2	151	66	0.437	0.615	0.822	0.279	-0.0011	0.468	-0.096	0.378	No
READ	3	151	40	0.265	0.385	0.661	0.493	-0.0008	0.329	-0.399	0.378	No
READ	4	151	31	0.205	0.275	0.545	0.492	0.0022	0.339	-0.772	0.478	No
READ	5	151	26	0.172	0.192	0.610	0.382	0.0094	0.377	0.677	0.142	No
READ	6	151	19	0.126	0.083	0.495	0.377	0.0044	0.321	0.372	0.142	No
SARC	2	232	105	0.453	0.742	0.955	0.069	0.0114	0.562	1.097	0.055	Yes
SARC	3	232	64	0.276	0.435	0.773	0.321	0.0025	0.400	0.193	0.312	No
SARC	4	232	43	0.185	0.303	0.557	0.480	-0.0027	0.303	-0.609	0.470	No
SARC	5	232	33	0.142	0.243	0.458	0.446	-0.0044	0.307	-0.970	0.555	No
SARC	6	232	25	0.108	0.145	0.439	0.408	-0.0007	0.296	-0.534	0.470	No
SKCM	2	466	219	0.470	0.612	0.791	0.322	0.0015	0.367	-0.512	0.473	No
SKCM	3	466	142	0.305	0.541	0.833	0.200	0.0033	0.528	0.370	0.397	No
SKCM	4	466	91	0.195	0.387	0.715	0.327	0.0067	0.438	-0.036	0.397	No
SKCM	5	466	81	0.174	0.311	0.527	0.386	0.0001	0.358	-0.528	0.473	No
SKCM	6	466	57	0.122	0.264	0.494	0.369	0.0012	0.339	-0.814	0.473	No
STAD	2	383	170	0.444	0.657	0.784	0.275	-0.0015	0.431	-1.962	0.900	No
STAD	3	383	125	0.326	0.451	0.735	0.348	0.0013	0.399	-0.411	0.395	No
STAD	4	383	81	0.211	0.353	0.698	0.320	0.0122	0.429	-0.186	0.321	No
STAD	5	383	63	0.164	0.298	0.687	0.285	0.0081	0.408	0.210	0.321	No
STAD	6	383	50	0.131	0.237	0.563	0.333	0.0050	0.341	-0.198	0.321	No
TGCT	2	140	56	0.400	0.768	0.948	0.058	0.0122	0.619	0.538	0.260	No
TGCT	3	140	38	0.271	0.450	0.733	0.339	0.0194	0.531	0.279	0.260	No
TGCT	4	140	32	0.229	0.262	0.580	0.438	-0.0070	0.409	-0.743	0.666	No
TGCT	5	140	13	0.093	0.145	0.471	0.439	0.0055	0.344	-0.955	0.666	No
TGCT	6	140	11	0.079	0.045	0.424	0.417	-0.0039	0.306	-1.309	0.666	No
THCA	2	484	197	0.407	0.707	0.852	0.225	0.0055	0.498	-1.229	0.721	No
THCA	3	484	149	0.308	0.592	0.867	0.181	0.0004	0.505	0.068	0.288	No
THCA	4	484	95	0.196	0.426	0.800	0.234	0.0108	0.431	0.667	0.265	No
THCA	5	484	83	0.171	0.309	0.734	0.248	0.0136	0.394	0.127	0.272	No
THCA	6	484	53	0.110	0.243	0.598	0.305	0.0162	0.379	-0.407	0.302	No
THYM	2	117	54	0.462	0.717	0.895	0.104	0.0114	0.668	-0.203	0.313	No
THYM	3	117	35	0.299	0.452	0.850	0.184	-0.0030	0.575	-0.208	0.313	No
THYM	4	117	19	0.162	0.251	0.759	0.286	0.0021	0.515	0.156	0.275	No
THYM	5	117	18	0.154	0.062	0.609	0.348	0.0149	0.442	-0.564	0.313	No
THYM	6	117	14	0.120	0.025	0.587	0.342	0.0245	0.445	0.044	0.275	No

Continued on next page

Table S6 (continued)

Cancer	k	n	Min. n	Min. frac.	Pred. strength	ARI	PAC	Sil. surplus	Cross-view ARI	Joint z	BH-FDR	Pass
UCEC	2	509	252	0.495	0.785	0.931	0.111	0.0088	0.604	-0.251	0.280	No
UCEC	3	509	144	0.283	0.562	0.834	0.215	0.0170	0.477	0.601	0.060	No
UCEC	4	509	108	0.212	0.488	0.873	0.148	0.0081	0.487	1.192	0.030	Yes
UCEC	5	509	78	0.153	0.344	0.670	0.280	0.0101	0.377	0.583	0.060	No
UCEC	6	509	71	0.139	0.297	0.641	0.270	0.0103	0.404	1.112	0.010	No
UCS	2	57	28	0.491	0.476	0.710	0.463	-0.0127	0.454	-0.719	0.603	No
UCS	3	57	13	0.228	0.208	0.641	0.419	-0.0027	0.406	-0.192	0.542	No
UCS	4	57	10	0.175	0.010	0.533	0.487	-0.0036	0.356	-0.288	0.542	No
UCS	5	57	5	0.088	0.000	0.455	0.487	-0.0136	0.307	-1.095	0.710	No
UCS	6	57	2	0.035	0.000	0.429	0.437	-0.0063	0.312	-0.517	0.603	No
UVM	2	80	36	0.450	0.610	0.850	0.276	-0.0085	0.544	-0.467	0.987	No
UVM	3	80	26	0.325	0.305	0.571	0.541	-0.0052	0.332	-0.636	0.987	No
UVM	4	80	12	0.150	0.078	0.450	0.559	-0.0105	0.313	-1.189	0.987	No
UVM	5	80	9	0.113	0.012	0.367	0.515	-0.0000	0.277	-1.394	0.987	No
UVM	6	80	7	0.087	0.000	0.372	0.472	-0.0225	0.276	-2.450	0.987	No

Alt text: Table S6. Stability and null-model evidence for candidate PRS resolutions from two to six across all 33 cancer types. Each row reports a cancer-resolution combination, group size, resampling and consensus metrics, null-model statistics, false-discovery rate and whether the common selection criteria were met.

Table S7: **Final patient-representation mode across 33 cancer types.**

Cancer	Patients	Selected k	Representation
ACC	77	–	Continuous pharmacological response axis
BLCA	401	–	Continuous pharmacological response axis
BRCA	964	4	Discrete PRS (k=4)
CESC	284	–	Continuous pharmacological response axis
CHOL	35	–	Continuous pharmacological response axis
COAD	427	3	Discrete PRS (k=3)
DLBC	47	–	Continuous pharmacological response axis
ESCA	182	–	Continuous pharmacological response axis
GBM	245	–	Continuous pharmacological response axis
HNSC	500	–	Continuous pharmacological response axis
KICH	66	–	Continuous pharmacological response axis
KIRC	372	–	Continuous pharmacological response axis
KIRP	277	–	Continuous pharmacological response axis
LAML	59	–	Continuous pharmacological response axis
LGG	507	–	Continuous pharmacological response axis
LIHC	362	–	Continuous pharmacological response axis
LUAD	505	–	Continuous pharmacological response axis
LUSC	482	–	Continuous pharmacological response axis
MESO	78	–	Continuous pharmacological response axis
OV	283	–	Continuous pharmacological response axis
PAAD	162	–	Continuous pharmacological response axis
PCPG	177	–	Continuous pharmacological response axis
PRAD	488	–	Continuous pharmacological response axis
READ	151	–	Continuous pharmacological response axis
SARC	232	2	Discrete PRS (k=2)
SKCM	466	–	Continuous pharmacological response axis
STAD	383	–	Continuous pharmacological response axis
TGCT	140	–	Continuous pharmacological response axis
THCA	484	–	Continuous pharmacological response axis
THYM	117	–	Continuous pharmacological response axis
UCEC	509	4	Discrete PRS (k=4)
UCS	57	–	Continuous pharmacological response axis
UVM	80	–	Continuous pharmacological response axis

Alt text: Table S7. Patient counts and final representation modes for all 33 cancer types. The selected resolution identifies stability-supported discrete PRSs; cancer types without a supported discrete solution are represented by a continuous pharmacological response axis.

Table S8: **PRS-construction-excluded-drug module profiles for all stability-supported PRSs.** Differences compare each PRS with all remaining patients of the same cancer type. BRCA, UCEC and SARC are displayed in the main text; COAD is reported in the Supplementary Information.

Cancer	PRS	n	Module	Mean difference	95% CI low	95% CI high
BRCA	PRS1	295	RTK–MAPK	0.0439	0.0367	0.0510
BRCA	PRS1	295	PI3K–AKT–mTOR	-0.0602	-0.0681	-0.0525
BRCA	PRS1	295	Apoptosis	-0.0155	-0.0230	-0.0077
BRCA	PRS1	295	DNA damage/cell cycle	-0.0015	-0.0062	0.0034
BRCA	PRS1	295	Epigenetic regulation	-0.0341	-0.0419	-0.0262
BRCA	PRS2	230	RTK–MAPK	-0.0081	-0.0160	-0.0000
BRCA	PRS2	230	PI3K–AKT–mTOR	-0.0408	-0.0484	-0.0328
BRCA	PRS2	230	Apoptosis	-0.0221	-0.0299	-0.0139
BRCA	PRS2	230	DNA damage/cell cycle	-0.0033	-0.0081	0.0018
BRCA	PRS2	230	Epigenetic regulation	-0.0664	-0.0748	-0.0584
BRCA	PRS3	242	RTK–MAPK	-0.0436	-0.0509	-0.0364
BRCA	PRS3	242	PI3K–AKT–mTOR	0.0837	0.0757	0.0917
BRCA	PRS3	242	Apoptosis	0.0273	0.0185	0.0359
BRCA	PRS3	242	DNA damage/cell cycle	-0.0154	-0.0208	-0.0101
BRCA	PRS3	242	Epigenetic regulation	0.0417	0.0327	0.0505
BRCA	PRS4	197	RTK–MAPK	0.0021	-0.0060	0.0102
BRCA	PRS4	197	PI3K–AKT–mTOR	0.0274	0.0191	0.0366
BRCA	PRS4	197	Apoptosis	0.0134	0.0041	0.0226
BRCA	PRS4	197	DNA damage/cell cycle	0.0235	0.0180	0.0294
BRCA	PRS4	197	Epigenetic regulation	0.0705	0.0621	0.0787
UCEC	PRS1	127	RTK–MAPK	0.0475	0.0358	0.0592
UCEC	PRS1	127	PI3K–AKT–mTOR	-0.0761	-0.0873	-0.0656
UCEC	PRS1	127	Apoptosis	-0.0530	-0.0651	-0.0415
UCEC	PRS1	127	DNA damage/cell cycle	-0.0216	-0.0299	-0.0136
UCEC	PRS1	127	Epigenetic regulation	-0.1175	-0.1280	-0.1065
UCEC	PRS2	125	RTK–MAPK	0.0059	-0.0058	0.0178
UCEC	PRS2	125	PI3K–AKT–mTOR	-0.0593	-0.0716	-0.0465
UCEC	PRS2	125	Apoptosis	-0.0005	-0.0130	0.0111
UCEC	PRS2	125	DNA damage/cell cycle	-0.0309	-0.0383	-0.0238
UCEC	PRS2	125	Epigenetic regulation	-0.0240	-0.0373	-0.0093
UCEC	PRS3	149	RTK–MAPK	-0.0096	-0.0201	-0.0000
UCEC	PRS3	149	PI3K–AKT–mTOR	0.0313	0.0198	0.0432
UCEC	PRS3	149	Apoptosis	0.0052	-0.0052	0.0158
UCEC	PRS3	149	DNA damage/cell cycle	0.0119	0.0034	0.0202
UCEC	PRS3	149	Epigenetic regulation	0.0218	0.0090	0.0335
UCEC	PRS4	108	RTK–MAPK	-0.0479	-0.0584	-0.0379
UCEC	PRS4	108	PI3K–AKT–mTOR	0.1122	0.1006	0.1240
UCEC	PRS4	108	Apoptosis	0.0535	0.0428	0.0638
UCEC	PRS4	108	DNA damage/cell cycle	0.0438	0.0351	0.0521
UCEC	PRS4	108	Epigenetic regulation	0.1313	0.1204	0.1425
SARC	PRS1	105	RTK–MAPK	0.0289	0.0142	0.0428
SARC	PRS1	105	PI3K–AKT–mTOR	-0.0675	-0.0818	-0.0523
SARC	PRS1	105	Apoptosis	-0.0092	-0.0228	0.0040
SARC	PRS1	105	DNA damage/cell cycle	-0.0142	-0.0242	-0.0041
SARC	PRS1	105	Epigenetic regulation	-0.0745	-0.0886	-0.0610
SARC	PRS2	127	RTK–MAPK	-0.0289	-0.0430	-0.0149
SARC	PRS2	127	PI3K–AKT–mTOR	0.0675	0.0517	0.0818
SARC	PRS2	127	Apoptosis	0.0092	-0.0033	0.0223
SARC	PRS2	127	DNA damage/cell cycle	0.0142	0.0044	0.0242
SARC	PRS2	127	Epigenetic regulation	0.0745	0.0598	0.0887
COAD	PRS1	152	RTK–MAPK	0.0562	0.0448	0.0680
COAD	PRS1	152	PI3K–AKT–mTOR	-0.0057	-0.0198	0.0079
COAD	PRS1	152	Apoptosis	0.0055	-0.0047	0.0154
COAD	PRS1	152	DNA damage/cell cycle	0.0140	0.0051	0.0233
COAD	PRS1	152	Epigenetic regulation	0.0149	0.0023	0.0276
COAD	PRS2	158	RTK–MAPK	0.0385	0.0261	0.0506
COAD	PRS2	158	PI3K–AKT–mTOR	-0.0825	-0.0951	-0.0705
COAD	PRS2	158	Apoptosis	-0.0340	-0.0439	-0.0240
COAD	PRS2	158	DNA damage/cell cycle	-0.0221	-0.0304	-0.0139
COAD	PRS2	158	Epigenetic regulation	-0.0894	-0.1004	-0.0790
COAD	PRS3	117	RTK–MAPK	-0.1099	-0.1202	-0.1004

Table S8 (continued)

Cancer	PRS	n	Module	Mean difference	95% CI low	95% CI high
COAD	PRS3	117	PI3K–AKT–mTOR	0.1033	0.0914	0.1147
COAD	PRS3	117	Apoptosis	0.0335	0.0237	0.0433
COAD	PRS3	117	DNA damage/cell cycle	0.0097	-0.0003	0.0196
COAD	PRS3	117	Epigenetic regulation	0.0876	0.0760	0.0995

Alt text: Table S8. Mechanism-module response profiles for stability-supported PRSs in BRCA, UCEC, SARC and COAD, using PRS-construction-excluded drugs. Rows identify a cancer type, PRS, patient count and module; estimates compare each PRS with the remaining patients and include bootstrap confidence intervals.

Table S9: **Association between main-text PRSs and reference classifications.**

Cancer	Total n	Matched n	Coverage	Cramér's V	ARI	NMI	Permutation P
BRCA	964	920	95.4%	0.281	0.062	0.086	0.00010
UCEC	509	486	95.5%	0.435	0.175	0.199	0.00010
SARC	232	232	100.0%	0.228	0.018	0.036	0.00320

Alt text: Table S9. Associations of BRCA, UCEC and SARC PRSs with their reference classifications. Columns report cohort size, matched-label coverage, Cramer's V, adjusted Rand index, normalized mutual information and permutation-test probability.

Table S10: **PRS-construction and PRS-construction-excluded drug composition and source-validation quality scores.**

Module	Set	Drugs	Mean	SD	Minimum	Maximum
Apoptosis	PRS-construction	8	0.286	0.266	0.038	0.849
Apoptosis	PRS-construction-excluded	8	0.732	0.150	0.516	0.953
DNA damage/cell cycle	PRS-construction	44	0.529	0.183	0.154	0.861
DNA damage/cell cycle	PRS-construction-excluded	8	0.806	0.110	0.624	0.931
Epigenetic regulation	PRS-construction	22	0.405	0.238	0.061	0.833
Epigenetic regulation	PRS-construction-excluded	8	0.791	0.114	0.674	0.934
PI3K–AKT–mTOR	PRS-construction	12	0.189	0.126	0.041	0.455
PI3K–AKT–mTOR	PRS-construction-excluded	8	0.514	0.214	0.121	0.740
RTK–MAPK	PRS-construction	34	0.407	0.248	0.039	0.951
RTK–MAPK	PRS-construction-excluded	8	0.898	0.067	0.810	0.986

Alt text: Table S10. Drug counts and source-validation quality-score summaries for the PRS-construction and PRS-construction-excluded sets in five mechanism modules. Columns report the number of drugs and the mean, standard deviation, minimum and maximum quality score for each module-set combination.

Table S11: **Complete results for the lineage-aware internal consistency analysis of known drug–cancer indications.** Each mapping is reported at the top-300, top-600 and top-1,000 predicted-sensitive patient thresholds.

Drug ID	Drug	TCGA indication codes	Threshold	Top ind.	Top other	Rest ind.	Rest other	Fisher OR	Haldane OR	95% CI (log2)	*P*	FDR
1021	Axitinib	KICH;KIRC;KIRP	300	1	299	714	8585	0.0402	0.0602	-6.37, -1.74	1.00e+00	1.00e+00
1021	Axitinib	KICH;KIRC;KIRP	600	2	598	713	8286	0.0389	0.0485	-6.16, -2.57	1.00e+00	1.00e+00
1021	Axitinib	KICH;KIRC;KIRP	1000	4	996	711	7888	0.0446	0.0501	-5.66, -2.98	1.00e+00	1.00e+00
1812	Bleomycin	CESC;DLBC;HNSC	300	141	159	690	8609	11.1	11.1	3.12, 3.81	4.41e-72	2.28e-71
1812	Bleomycin	CESC;DLBC;HNSC	600	235	365	596	8403	9.08	9.08	2.92, 3.45	9.38e-103	9.69e-102
1812	Bleomycin	CESC;DLBC;HNSC	1000	318	682	513	8086	7.35	7.35	2.65, 3.11	2.68e-114	2.76e-113
1005	Cisplatin	OV	300	0	300	283	9016	0	0.0529	-8.25, -0.23	1.00e+00	1.00e+00
1005	Cisplatin	OV	600	0	600	283	8716	0	0.0256	-9.29, -1.28	1.00e+00	1.00e+00
1005	Cisplatin	OV	1000	1	999	282	8317	0.0295	0.0442	-6.82, -2.18	1.00e+00	1.00e+00
1083	Crizotinib	LUAD;LUSC	300	0	300	987	8312	0	0.014	-10.16, -2.15	1.00e+00	1.00e+00
1083	Crizotinib	LUAD;LUSC	600	3	597	984	8015	0.0409	0.0477	-5.91, -2.87	1.00e+00	1.00e+00
1083	Crizotinib	LUAD;LUSC	1000	25	975	962	7637	0.204	0.207	-2.84, -1.69	1.00e+00	1.00e+00
1512	Cyclophosphamide	BRCA	300	3	297	961	8338	0.0876	0.102	-4.82, -1.77	1.00e+00	1.00e+00
1512	Cyclophosphamide	BRCA	600	18	582	946	8053	0.263	0.27	-2.56, -1.21	1.00e+00	1.00e+00
1512	Cyclophosphamide	BRCA	1000	37	963	927	7672	0.318	0.322	-2.12, -1.15	1.00e+00	1.00e+00
1006	Cytarabine	LAML	300	59	241	0	9299	inf	4.58e+03	8.14, 16.18	4.17e-92	3.24e-91
1006	Cytarabine	LAML	600	59	541	0	8999	inf	1.98e+03	6.93, 14.97	5.71e-73	2.95e-72
1006	Cytarabine	LAML	1000	59	941	0	8599	inf	1.09e+03	6.07, 14.10	2.33e-59	1.21e-58
1373	Dabrafenib	SKCM	300	124	176	342	8957	18.5	18.4	3.84, 4.57	6.23e-87	3.86e-86
1373	Dabrafenib	SKCM	600	256	344	210	8789	31.1	31.1	4.65, 5.26	5.14e-201	7.97e-200
1373	Dabrafenib	SKCM	1000	319	681	147	8452	26.9	26.9	4.44, 5.05	1.08e-216	1.67e-215
1815	Dacarbazine	SKCM	300	10	290	456	8843	0.669	0.7	-1.41, 0.38	9.23e-01	1.00e+00
1815	Dacarbazine	SKCM	600	52	548	414	8585	1.97	1.98	0.55, 1.42	2.78e-05	1.08e-04
1815	Dacarbazine	SKCM	1000	92	908	374	8225	2.23	2.24	0.82, 1.50	5.77e-10	2.24e-09
1811	Dactinomycin	TGCT	300	29	271	111	9188	8.86	8.95	2.55, 3.77	2.27e-16	1.01e-15
1811	Dactinomycin	TGCT	600	48	552	92	8907	8.42	8.45	2.56, 3.60	1.05e-23	4.64e-23
1811	Dactinomycin	TGCT	1000	70	930	70	8529	9.17	9.17	2.71, 3.68	1.47e-32	6.51e-32
1007	Docetaxel	BRCA;HNSC;LUAD;LUSC;PRAD;STAD	300	94	206	3228	6071	0.858	0.861	-0.57, 0.14	8.99e-01	1.00e+00
1007	Docetaxel	BRCA;HNSC;LUAD;LUSC;PRAD;STAD	600	224	376	3098	5901	1.13	1.14	-0.06, 0.43	8.05e-02	2.77e-01
1007	Docetaxel	BRCA;HNSC;LUAD;LUSC;PRAD;STAD	1000	401	599	2921	5678	1.3	1.3	0.19, 0.57	7.60e-05	2.62e-04
1511	Epirubicin	BRCA	300	1	299	963	8336	0.029	0.0433	-6.84, -2.21	1.00e+00	1.00e+00
1511	Epirubicin	BRCA	600	8	592	956	8043	0.114	0.121	-4.03, -2.07	1.00e+00	1.00e+00
1511	Epirubicin	BRCA	1000	21	979	943	7656	0.174	0.178	-3.11, -1.86	1.00e+00	1.00e+00
1168	Erlotinib	LUAD;LUSC;PAAD	300	14	286	1135	8164	0.352	0.364	-2.22, -0.69	1.00e+00	1.00e+00
1168	Erlotinib	LUAD;LUSC;PAAD	600	41	559	1108	7891	0.522	0.528	-1.39, -0.46	1.00e+00	1.00e+00
1168	Erlotinib	LUAD;LUSC;PAAD	1000	103	897	1046	7553	0.829	0.832	-0.57, 0.04	9.64e-01	1.00e+00
1010	Gefitinib	LUAD;LUSC	300	19	281	968	8331	0.582	0.596	-1.42, -0.08	9.94e-01	1.00e+00
1010	Gefitinib	LUAD;LUSC	600	50	550	937	8062	0.782	0.789	-0.77, 0.08	9.58e-01	1.00e+00
1010	Gefitinib	LUAD;LUSC	1000	108	892	879	7720	1.06	1.07	-0.21, 0.40	3.01e-01	8.47e-01
1190	Gemcitabine	LUAD;LUSC;OV;PAAD	300	17	283	1415	7884	0.335	0.344	-2.24, -0.84	1.00e+00	1.00e+00
1190	Gemcitabine	LUAD;LUSC;OV;PAAD	600	59	541	1373	7626	0.606	0.61	-1.11, -0.32	1.00e+00	1.00e+00
1190	Gemcitabine	LUAD;LUSC;OV;PAAD	1000	122	878	1310	7289	0.773	0.776	-0.65, -0.08	9.96e-01	1.00e+00
1088	Irinotecan	PAAD	300	0	300	162	9137	0	0.0936	-7.43, 0.59	1.00e+00	1.00e+00
1088	Irinotecan	PAAD	600	0	600	162	8837	0	0.0453	-8.47, -0.46	1.00e+00	1.00e+00
1088	Irinotecan	PAAD	1000	0	1000	162	8437	0	0.0259	-9.27, -1.26	1.00e+00	1.00e+00

Continued on next page

Table S11 (continued)

Drug ID	Drug	TCGA indication codes	Threshold	Top ind.	Top other	Rest ind.	Rest other	Fisher OR	Haldane OR	95% CI (log2)	*P*	FDR
1558	Lapatinib	BRCA	300	11	289	953	8346	0.333	0.348	-2.38, -0.67	1.00e+00	1.00e+00
1558	Lapatinib	BRCA	600	29	571	935	8064	0.438	0.445	-1.71, -0.63	1.00e+00	1.00e+00
1558	Lapatinib	BRCA	1000	58	942	906	7693	0.523	0.527	-1.32, -0.53	1.00e+00	1.00e+00
1008	Methotrexate	BRCA;HNSC;LUAD;LUSC	300	49	251	2402	6897	0.561	0.565	-1.27, -0.38	1.00e+00	1.00e+00
1008	Methotrexate	BRCA;HNSC;LUAD;LUSC	600	118	482	2333	6666	0.699	0.702	-0.81, -0.21	1.00e+00	1.00e+00
1008	Methotrexate	BRCA;HNSC;LUAD;LUSC	1000	250	750	2201	6398	0.969	0.97	-0.26, 0.17	6.71e-01	1.00e+00
1810	Mitoxantrone	LAML	300	59	241	0	9299	inf	4.58e+03	8.14, 16.18	4.17e-92	3.24e-91
1810	Mitoxantrone	LAML	600	59	541	0	8999	inf	1.98e+03	6.93, 14.97	5.71e-73	2.95e-72
1810	Mitoxantrone	LAML	1000	59	941	0	8599	inf	1.09e+03	6.07, 14.10	2.33e-59	1.21e-58
1177	Niraparib	OV	300	0	300	283	9016	0	0.0529	-8.25, -0.23	1.00e+00	1.00e+00
1177	Niraparib	OV	600	0	600	283	8716	0	0.0256	-9.29, -1.28	1.00e+00	1.00e+00
1177	Niraparib	OV	1000	2	998	281	8318	0.0593	0.074	-5.56, -1.96	1.00e+00	1.00e+00
1017	Olaparib	OV	300	0	300	283	9016	0	0.0529	-8.25, -0.23	1.00e+00	1.00e+00
1017	Olaparib	OV	600	0	600	283	8716	0	0.0256	-9.29, -1.28	1.00e+00	1.00e+00
1017	Olaparib	OV	1000	2	998	281	8318	0.0593	0.074	-5.56, -1.96	1.00e+00	1.00e+00
1080	Paclitaxel	BRCA;LUAD;LUSC;OV	300	31	269	2203	7096	0.371	0.376	-1.95, -0.87	1.00e+00	1.00e+00
1080	Paclitaxel	BRCA;LUAD;LUSC;OV	600	94	506	2140	6859	0.595	0.598	-1.07, -0.42	1.00e+00	1.00e+00
1080	Paclitaxel	BRCA;LUAD;LUSC;OV	1000	182	818	2052	6547	0.71	0.711	-0.73, -0.25	1.00e+00	1.00e+00
1175	Rucaparib	OV	300	0	300	283	9016	0	0.0529	-8.25, -0.23	1.00e+00	1.00e+00
1175	Rucaparib	OV	600	0	600	283	8716	0	0.0256	-9.29, -1.28	1.00e+00	1.00e+00
1175	Rucaparib	OV	1000	0	1000	283	8316	0	0.0147	-10.10, -2.09	1.00e+00	1.00e+00
1085	Sorafenib	KICH;KIRC;KIRP;LIHC;THCA	300	18	282	1543	7756	0.321	0.329	-2.29, -0.92	1.00e+00	1.00e+00
1085	Sorafenib	KICH;KIRC;KIRP;LIHC;THCA	600	64	536	1497	7502	0.598	0.602	-1.11, -0.35	1.00e+00	1.00e+00
1085	Sorafenib	KICH;KIRC;KIRP;LIHC;THCA	1000	127	873	1434	7165	0.727	0.729	-0.74, -0.18	1.00e+00	1.00e+00
1199	Tamoxifen	BRCA	300	18	282	946	8353	0.564	0.578	-1.48, -0.11	9.96e-01	1.00e+00
1199	Tamoxifen	BRCA	600	49	551	915	8084	0.786	0.793	-0.77, 0.10	9.54e-01	1.00e+00
1199	Tamoxifen	BRCA	1000	97	903	867	7732	0.958	0.962	-0.37, 0.26	6.65e-01	1.00e+00
1375	Temozolomide	GBM;LGG	300	5	295	747	8552	0.194	0.213	-3.45, -1.01	1.00e+00	1.00e+00
1375	Temozolomide	GBM;LGG	600	14	586	738	8261	0.267	0.277	-2.61, -1.09	1.00e+00	1.00e+00
1375	Temozolomide	GBM;LGG	1000	48	952	704	7895	0.565	0.571	-1.24, -0.38	1.00e+00	1.00e+00
1016	Temsirolimus	KICH;KIRC;KIRP	300	11	289	704	8595	0.465	0.485	-1.90, -0.19	9.98e-01	1.00e+00
1016	Temsirolimus	KICH;KIRC;KIRP	600	33	567	682	8317	0.71	0.719	-0.99, 0.04	9.78e-01	1.00e+00
1016	Temsirolimus	KICH;KIRC;KIRP	1000	53	947	662	7937	0.671	0.677	-0.98, -0.15	9.98e-01	1.00e+00
1808	Topotecan	CESC;OV	300	10	290	557	8742	0.541	0.567	-1.72, 0.08	9.86e-01	1.00e+00
1808	Topotecan	CESC;OV	600	32	568	535	8464	0.891	0.904	-0.67, 0.38	7.56e-01	1.00e+00
1808	Topotecan	CESC;OV	1000	72	928	495	8104	1.27	1.28	-0.02, 0.72	4.18e-02	1.29e-01
1372	Trametinib	SKCM	300	163	137	303	8996	35.3	35.2	4.77, 5.51	8.55e-142	2.65e-140
1372	Trametinib	SKCM	600	256	344	210	8789	31.1	31.1	4.65, 5.26	5.14e-201	7.97e-200
1372	Trametinib	SKCM	1000	332	668	134	8465	31.4	31.3	4.66, 5.28	1.05e-235	3.27e-234
1009	Tretinoin	LAML	300	59	241	0	9299	inf	4.58e+03	8.14, 16.18	4.17e-92	3.24e-91
1009	Tretinoin	LAML	600	59	541	0	8999	inf	1.98e+03	6.93, 14.97	5.71e-73	2.95e-72
1009	Tretinoin	LAML	1000	59	941	0	8599	inf	1.09e+03	6.07, 14.10	2.33e-59	1.21e-58
1004	Vinblastine	BRCA;TGCT	300	26	274	1078	8221	0.724	0.736	-1.02, 0.14	9.55e-01	1.00e+00
1004	Vinblastine	BRCA;TGCT	600	52	548	1052	7947	0.717	0.723	-0.89, -0.05	9.92e-01	1.00e+00
1004	Vinblastine	BRCA;TGCT	1000	83	917	1021	7578	0.672	0.675	-0.90, -0.23	1.00e+00	1.00e+00
2048	Vinorelbine	LUAD;LUSC	300	19	281	968	8331	0.582	0.596	-1.42, -0.08	9.94e-01	1.00e+00
2048	Vinorelbine	LUAD;LUSC	600	49	551	938	8061	0.764	0.771	-0.81, 0.06	9.70e-01	1.00e+00
2048	Vinorelbine	LUAD;LUSC	1000	93	907	894	7705	0.884	0.888	-0.50, 0.15	8.73e-01	1.00e+00

Alt text: Table S11. Complete lineage-aware internal consistency results for known drug–cancer indications at three predicted-sensitive patient thresholds. Each row reports a drug-indication mapping, contingency counts, odds ratios, confidence intervals, test probability and false-discovery rate.

Table S12: **Predictive-uncertainty calibration and patient-ranking robustness.** Calibration metrics are reported for each held-out cell-line test split before and after applying the corresponding validation-fitted scalar. Penalty sensitivity is summarized relative to $\lambda_{\text{unc}} = 0.02$.

Split or analysis	Variant	Spearman	Gaussian NLL	Coverage 50%	Coverage 80%	Coverage 95%
Split 01	Raw	0.0797	1.444	0.389	0.657	0.842
Split 01	Validation-scaled	0.0797	1.266	0.542	0.828	0.950
Split 02	Raw	0.0289	1.645	0.353	0.603	0.792
Split 02	Validation-scaled	0.0289	1.305	0.540	0.822	0.947
Split 03	Raw	0.0346	1.658	0.336	0.588	0.783
Split 03	Validation-scaled	0.0346	1.281	0.561	0.845	0.960
Split 04	Raw	0.0511	1.557	0.376	0.637	0.821
Split 04	Validation-scaled	0.0511	1.313	0.542	0.822	0.950
Split 05	Raw	0.0714	1.722	0.330	0.577	0.766
Split 05	Validation-scaled	0.0714	1.262	0.543	0.823	0.948
Pooled	Raw	0.0506	1.606	0.357	0.612	0.800
Pooled	Validation-scaled	0.0539	1.286	0.545	0.828	0.951

Penalty	Median rank Spearman	Minimum rank Spearman	Minimum top-600 overlap	Minimum top-1,000 overlap
0	0.999997	0.999978	0.9917	0.9920
0.01	0.999999	0.999994	0.9950	0.9950
0.02	1.000000	1.000000	1.0000	1.0000
0.05	0.999994	0.999953	0.9917	0.9920

Cancer	k at 0.02	k at 0	ARI	NMI
BRCA	4	5	0.955	0.935
UCEC	4	4	0.983	0.979
SARC	2	2	1.000	1.000

Alt text: Table S12. Predictive-uncertainty calibration and uncertainty-penalty sensitivity. The first block compares raw and validation-scaled uncertainty on five held-out cell-line test splits; the second reports patient-ranking correlations and top-ranked overlap; the third reports selected PRS resolutions and partition agreement with and without the penalty.

Table S13: **Pan-cancer sensitivity to the study-defined ARI and PAC thresholds.** Group-size requirements and within-cancer BH-FDR < 0.10 were held fixed.

Cancer	ARI cutoff	PAC cutoff	Selected representation
ACC	0.80	0.15	Continuous pharmacological response axis
ACC	0.80	0.20	Continuous pharmacological response axis
ACC	0.80	0.25	Continuous pharmacological response axis
ACC	0.85	0.15	Continuous pharmacological response axis
ACC	0.85	0.20	Continuous pharmacological response axis
ACC	0.85	0.25	Continuous pharmacological response axis
ACC	0.90	0.15	Continuous pharmacological response axis
ACC	0.90	0.20	Continuous pharmacological response axis
ACC	0.90	0.25	Continuous pharmacological response axis
BLCA	0.80	0.15	Continuous pharmacological response axis
BLCA	0.80	0.20	Continuous pharmacological response axis
BLCA	0.80	0.25	Continuous pharmacological response axis
BLCA	0.85	0.15	Continuous pharmacological response axis
BLCA	0.85	0.20	Continuous pharmacological response axis
BLCA	0.85	0.25	Continuous pharmacological response axis
BLCA	0.90	0.15	Continuous pharmacological response axis
BLCA	0.90	0.20	Continuous pharmacological response axis
BLCA	0.90	0.25	Continuous pharmacological response axis
BRCA	0.80	0.15	Continuous pharmacological response axis
BRCA	0.80	0.20	Discrete PRS (k=4)
BRCA	0.80	0.25	Discrete PRS (k=4)
BRCA	0.85	0.15	Continuous pharmacological response axis
BRCA	0.85	0.20	Discrete PRS (k=4)
BRCA	0.85	0.25	Discrete PRS (k=4)
BRCA	0.90	0.15	Continuous pharmacological response axis
BRCA	0.90	0.20	Continuous pharmacological response axis
BRCA	0.90	0.25	Continuous pharmacological response axis
CESC	0.80	0.15	Continuous pharmacological response axis
CESC	0.80	0.20	Continuous pharmacological response axis
CESC	0.80	0.25	Continuous pharmacological response axis
CESC	0.85	0.15	Continuous pharmacological response axis
CESC	0.85	0.20	Continuous pharmacological response axis
CESC	0.85	0.25	Continuous pharmacological response axis
CESC	0.90	0.15	Continuous pharmacological response axis
CESC	0.90	0.20	Continuous pharmacological response axis
CESC	0.90	0.25	Continuous pharmacological response axis
CHOL	0.80	0.15	Continuous pharmacological response axis
CHOL	0.80	0.20	Continuous pharmacological response axis
CHOL	0.80	0.25	Continuous pharmacological response axis
CHOL	0.85	0.15	Continuous pharmacological response axis
CHOL	0.85	0.20	Continuous pharmacological response axis
CHOL	0.85	0.25	Continuous pharmacological response axis
CHOL	0.90	0.15	Continuous pharmacological response axis
CHOL	0.90	0.20	Continuous pharmacological response axis
CHOL	0.90	0.25	Continuous pharmacological response axis
COAD	0.80	0.15	Discrete PRS (k=3)
COAD	0.80	0.20	Discrete PRS (k=3)
COAD	0.80	0.25	Discrete PRS (k=3)
COAD	0.85	0.15	Discrete PRS (k=3)
COAD	0.85	0.20	Discrete PRS (k=3)
COAD	0.85	0.25	Discrete PRS (k=3)

Table S13 (continued)

Cancer	ARI cutoff	PAC cutoff	Selected representation
COAD	0.90	0.15	Continuous pharmacological response axis
COAD	0.90	0.20	Continuous pharmacological response axis
COAD	0.90	0.25	Continuous pharmacological response axis
DLBC	0.80	0.15	Continuous pharmacological response axis
DLBC	0.80	0.20	Continuous pharmacological response axis
DLBC	0.80	0.25	Continuous pharmacological response axis
DLBC	0.85	0.15	Continuous pharmacological response axis
DLBC	0.85	0.20	Continuous pharmacological response axis
DLBC	0.85	0.25	Continuous pharmacological response axis
DLBC	0.90	0.15	Continuous pharmacological response axis
DLBC	0.90	0.20	Continuous pharmacological response axis
DLBC	0.90	0.25	Continuous pharmacological response axis
ESCA	0.80	0.15	Continuous pharmacological response axis
ESCA	0.80	0.20	Continuous pharmacological response axis
ESCA	0.80	0.25	Continuous pharmacological response axis
ESCA	0.85	0.15	Continuous pharmacological response axis
ESCA	0.85	0.20	Continuous pharmacological response axis
ESCA	0.85	0.25	Continuous pharmacological response axis
ESCA	0.90	0.15	Continuous pharmacological response axis
ESCA	0.90	0.20	Continuous pharmacological response axis
ESCA	0.90	0.25	Continuous pharmacological response axis
GBM	0.80	0.15	Continuous pharmacological response axis
GBM	0.80	0.20	Continuous pharmacological response axis
GBM	0.80	0.25	Continuous pharmacological response axis
GBM	0.85	0.15	Continuous pharmacological response axis
GBM	0.85	0.20	Continuous pharmacological response axis
GBM	0.85	0.25	Continuous pharmacological response axis
GBM	0.90	0.15	Continuous pharmacological response axis
GBM	0.90	0.20	Continuous pharmacological response axis
GBM	0.90	0.25	Continuous pharmacological response axis
HNSC	0.80	0.15	Continuous pharmacological response axis
HNSC	0.80	0.20	Continuous pharmacological response axis
HNSC	0.80	0.25	Continuous pharmacological response axis
HNSC	0.85	0.15	Continuous pharmacological response axis
HNSC	0.85	0.20	Continuous pharmacological response axis
HNSC	0.85	0.25	Continuous pharmacological response axis
HNSC	0.90	0.15	Continuous pharmacological response axis
HNSC	0.90	0.20	Continuous pharmacological response axis
HNSC	0.90	0.25	Continuous pharmacological response axis
KICH	0.80	0.15	Continuous pharmacological response axis
KICH	0.80	0.20	Continuous pharmacological response axis
KICH	0.80	0.25	Continuous pharmacological response axis
KICH	0.85	0.15	Continuous pharmacological response axis
KICH	0.85	0.20	Continuous pharmacological response axis
KICH	0.85	0.25	Continuous pharmacological response axis
KICH	0.90	0.15	Continuous pharmacological response axis
KICH	0.90	0.20	Continuous pharmacological response axis
KICH	0.90	0.25	Continuous pharmacological response axis
KIRC	0.80	0.15	Continuous pharmacological response axis
KIRC	0.80	0.20	Continuous pharmacological response axis
KIRC	0.80	0.25	Continuous pharmacological response axis
KIRC	0.85	0.15	Continuous pharmacological response axis

Table S13 (continued)

Cancer	ARI cutoff	PAC cutoff	Selected representation
KIRC	0.85	0.20	Continuous pharmacological response axis
KIRC	0.85	0.25	Continuous pharmacological response axis
KIRC	0.90	0.15	Continuous pharmacological response axis
KIRC	0.90	0.20	Continuous pharmacological response axis
KIRC	0.90	0.25	Continuous pharmacological response axis
KIRP	0.80	0.15	Continuous pharmacological response axis
KIRP	0.80	0.20	Continuous pharmacological response axis
KIRP	0.80	0.25	Continuous pharmacological response axis
KIRP	0.85	0.15	Continuous pharmacological response axis
KIRP	0.85	0.20	Continuous pharmacological response axis
KIRP	0.85	0.25	Continuous pharmacological response axis
KIRP	0.90	0.15	Continuous pharmacological response axis
KIRP	0.90	0.20	Continuous pharmacological response axis
KIRP	0.90	0.25	Continuous pharmacological response axis
LAML	0.80	0.15	Continuous pharmacological response axis
LAML	0.80	0.20	Continuous pharmacological response axis
LAML	0.80	0.25	Continuous pharmacological response axis
LAML	0.85	0.15	Continuous pharmacological response axis
LAML	0.85	0.20	Continuous pharmacological response axis
LAML	0.85	0.25	Continuous pharmacological response axis
LAML	0.90	0.15	Continuous pharmacological response axis
LAML	0.90	0.20	Continuous pharmacological response axis
LAML	0.90	0.25	Continuous pharmacological response axis
LGG	0.80	0.15	Continuous pharmacological response axis
LGG	0.80	0.20	Continuous pharmacological response axis
LGG	0.80	0.25	Continuous pharmacological response axis
LGG	0.85	0.15	Continuous pharmacological response axis
LGG	0.85	0.20	Continuous pharmacological response axis
LGG	0.85	0.25	Continuous pharmacological response axis
LGG	0.90	0.15	Continuous pharmacological response axis
LGG	0.90	0.20	Continuous pharmacological response axis
LGG	0.90	0.25	Continuous pharmacological response axis
LIHC	0.80	0.15	Continuous pharmacological response axis
LIHC	0.80	0.20	Continuous pharmacological response axis
LIHC	0.80	0.25	Continuous pharmacological response axis
LIHC	0.85	0.15	Continuous pharmacological response axis
LIHC	0.85	0.20	Continuous pharmacological response axis
LIHC	0.85	0.25	Continuous pharmacological response axis
LIHC	0.90	0.15	Continuous pharmacological response axis
LIHC	0.90	0.20	Continuous pharmacological response axis
LIHC	0.90	0.25	Continuous pharmacological response axis
LUAD	0.80	0.15	Continuous pharmacological response axis
LUAD	0.80	0.20	Continuous pharmacological response axis
LUAD	0.80	0.25	Continuous pharmacological response axis
LUAD	0.85	0.15	Continuous pharmacological response axis
LUAD	0.85	0.20	Continuous pharmacological response axis
LUAD	0.85	0.25	Continuous pharmacological response axis
LUAD	0.90	0.15	Continuous pharmacological response axis
LUAD	0.90	0.20	Continuous pharmacological response axis
LUAD	0.90	0.25	Continuous pharmacological response axis
LUSC	0.80	0.15	Continuous pharmacological response axis
LUSC	0.80	0.20	Continuous pharmacological response axis

Table S13 (continued)

Cancer	ARI cutoff	PAC cutoff	Selected representation
LUSC	0.80	0.25	Continuous pharmacological response axis
LUSC	0.85	0.15	Continuous pharmacological response axis
LUSC	0.85	0.20	Continuous pharmacological response axis
LUSC	0.85	0.25	Continuous pharmacological response axis
LUSC	0.90	0.15	Continuous pharmacological response axis
LUSC	0.90	0.20	Continuous pharmacological response axis
LUSC	0.90	0.25	Continuous pharmacological response axis
MESO	0.80	0.15	Continuous pharmacological response axis
MESO	0.80	0.20	Continuous pharmacological response axis
MESO	0.80	0.25	Continuous pharmacological response axis
MESO	0.85	0.15	Continuous pharmacological response axis
MESO	0.85	0.20	Continuous pharmacological response axis
MESO	0.85	0.25	Continuous pharmacological response axis
MESO	0.90	0.15	Continuous pharmacological response axis
MESO	0.90	0.20	Continuous pharmacological response axis
MESO	0.90	0.25	Continuous pharmacological response axis
OV	0.80	0.15	Continuous pharmacological response axis
OV	0.80	0.20	Continuous pharmacological response axis
OV	0.80	0.25	Continuous pharmacological response axis
OV	0.85	0.15	Continuous pharmacological response axis
OV	0.85	0.20	Continuous pharmacological response axis
OV	0.85	0.25	Continuous pharmacological response axis
OV	0.90	0.15	Continuous pharmacological response axis
OV	0.90	0.20	Continuous pharmacological response axis
OV	0.90	0.25	Continuous pharmacological response axis
PAAD	0.80	0.15	Continuous pharmacological response axis
PAAD	0.80	0.20	Continuous pharmacological response axis
PAAD	0.80	0.25	Continuous pharmacological response axis
PAAD	0.85	0.15	Continuous pharmacological response axis
PAAD	0.85	0.20	Continuous pharmacological response axis
PAAD	0.85	0.25	Continuous pharmacological response axis
PAAD	0.90	0.15	Continuous pharmacological response axis
PAAD	0.90	0.20	Continuous pharmacological response axis
PAAD	0.90	0.25	Continuous pharmacological response axis
PCPG	0.80	0.15	Continuous pharmacological response axis
PCPG	0.80	0.20	Continuous pharmacological response axis
PCPG	0.80	0.25	Continuous pharmacological response axis
PCPG	0.85	0.15	Continuous pharmacological response axis
PCPG	0.85	0.20	Continuous pharmacological response axis
PCPG	0.85	0.25	Continuous pharmacological response axis
PCPG	0.90	0.15	Continuous pharmacological response axis
PCPG	0.90	0.20	Continuous pharmacological response axis
PCPG	0.90	0.25	Continuous pharmacological response axis
PRAD	0.80	0.15	Continuous pharmacological response axis
PRAD	0.80	0.20	Continuous pharmacological response axis
PRAD	0.80	0.25	Continuous pharmacological response axis
PRAD	0.85	0.15	Continuous pharmacological response axis
PRAD	0.85	0.20	Continuous pharmacological response axis
PRAD	0.85	0.25	Continuous pharmacological response axis
PRAD	0.90	0.15	Continuous pharmacological response axis
PRAD	0.90	0.20	Continuous pharmacological response axis
PRAD	0.90	0.25	Continuous pharmacological response axis

Table S13 (continued)

Cancer	ARI cutoff	PAC cutoff	Selected representation
READ	0.80	0.15	Continuous pharmacological response axis
READ	0.80	0.20	Continuous pharmacological response axis
READ	0.80	0.25	Continuous pharmacological response axis
READ	0.85	0.15	Continuous pharmacological response axis
READ	0.85	0.20	Continuous pharmacological response axis
READ	0.85	0.25	Continuous pharmacological response axis
READ	0.90	0.15	Continuous pharmacological response axis
READ	0.90	0.20	Continuous pharmacological response axis
READ	0.90	0.25	Continuous pharmacological response axis
SARC	0.80	0.15	Discrete PRS (k=2)
SARC	0.80	0.20	Discrete PRS (k=2)
SARC	0.80	0.25	Discrete PRS (k=2)
SARC	0.85	0.15	Discrete PRS (k=2)
SARC	0.85	0.20	Discrete PRS (k=2)
SARC	0.85	0.25	Discrete PRS (k=2)
SARC	0.90	0.15	Discrete PRS (k=2)
SARC	0.90	0.20	Discrete PRS (k=2)
SARC	0.90	0.25	Discrete PRS (k=2)
SKCM	0.80	0.15	Continuous pharmacological response axis
SKCM	0.80	0.20	Continuous pharmacological response axis
SKCM	0.80	0.25	Continuous pharmacological response axis
SKCM	0.85	0.15	Continuous pharmacological response axis
SKCM	0.85	0.20	Continuous pharmacological response axis
SKCM	0.85	0.25	Continuous pharmacological response axis
SKCM	0.90	0.15	Continuous pharmacological response axis
SKCM	0.90	0.20	Continuous pharmacological response axis
SKCM	0.90	0.25	Continuous pharmacological response axis
STAD	0.80	0.15	Continuous pharmacological response axis
STAD	0.80	0.20	Continuous pharmacological response axis
STAD	0.80	0.25	Continuous pharmacological response axis
STAD	0.85	0.15	Continuous pharmacological response axis
STAD	0.85	0.20	Continuous pharmacological response axis
STAD	0.85	0.25	Continuous pharmacological response axis
STAD	0.90	0.15	Continuous pharmacological response axis
STAD	0.90	0.20	Continuous pharmacological response axis
STAD	0.90	0.25	Continuous pharmacological response axis
TGCT	0.80	0.15	Continuous pharmacological response axis
TGCT	0.80	0.20	Continuous pharmacological response axis
TGCT	0.80	0.25	Continuous pharmacological response axis
TGCT	0.85	0.15	Continuous pharmacological response axis
TGCT	0.85	0.20	Continuous pharmacological response axis
TGCT	0.85	0.25	Continuous pharmacological response axis
TGCT	0.90	0.15	Continuous pharmacological response axis
TGCT	0.90	0.20	Continuous pharmacological response axis
TGCT	0.90	0.25	Continuous pharmacological response axis
THCA	0.80	0.15	Continuous pharmacological response axis
THCA	0.80	0.20	Continuous pharmacological response axis
THCA	0.80	0.25	Continuous pharmacological response axis
THCA	0.85	0.15	Continuous pharmacological response axis
THCA	0.85	0.20	Continuous pharmacological response axis
THCA	0.85	0.25	Continuous pharmacological response axis
THCA	0.90	0.15	Continuous pharmacological response axis

Table S13 (continued)

Cancer	ARI cutoff	PAC cutoff	Selected representation
THCA	0.90	0.20	Continuous pharmacological response axis
THCA	0.90	0.25	Continuous pharmacological response axis
THYM	0.80	0.15	Continuous pharmacological response axis
THYM	0.80	0.20	Continuous pharmacological response axis
THYM	0.80	0.25	Continuous pharmacological response axis
THYM	0.85	0.15	Continuous pharmacological response axis
THYM	0.85	0.20	Continuous pharmacological response axis
THYM	0.85	0.25	Continuous pharmacological response axis
THYM	0.90	0.15	Continuous pharmacological response axis
THYM	0.90	0.20	Continuous pharmacological response axis
THYM	0.90	0.25	Continuous pharmacological response axis
UCEC	0.80	0.15	Discrete PRS (k=4)
UCEC	0.80	0.20	Discrete PRS (k=4)
UCEC	0.80	0.25	Discrete PRS (k=4)
UCEC	0.85	0.15	Discrete PRS (k=4)
UCEC	0.85	0.20	Discrete PRS (k=4)
UCEC	0.85	0.25	Discrete PRS (k=4)
UCEC	0.90	0.15	Continuous pharmacological response axis
UCEC	0.90	0.20	Continuous pharmacological response axis
UCEC	0.90	0.25	Continuous pharmacological response axis
UCS	0.80	0.15	Continuous pharmacological response axis
UCS	0.80	0.20	Continuous pharmacological response axis
UCS	0.80	0.25	Continuous pharmacological response axis
UCS	0.85	0.15	Continuous pharmacological response axis
UCS	0.85	0.20	Continuous pharmacological response axis
UCS	0.85	0.25	Continuous pharmacological response axis
UCS	0.90	0.15	Continuous pharmacological response axis
UCS	0.90	0.20	Continuous pharmacological response axis
UCS	0.90	0.25	Continuous pharmacological response axis
UVM	0.80	0.15	Continuous pharmacological response axis
UVM	0.80	0.20	Continuous pharmacological response axis
UVM	0.80	0.25	Continuous pharmacological response axis
UVM	0.85	0.15	Continuous pharmacological response axis
UVM	0.85	0.20	Continuous pharmacological response axis
UVM	0.85	0.25	Continuous pharmacological response axis
UVM	0.90	0.15	Continuous pharmacological response axis
UVM	0.90	0.20	Continuous pharmacological response axis
UVM	0.90	0.25	Continuous pharmacological response axis

Alt text: Table S13. PRS-resolution sensitivity across combinations of resampling adjusted Rand index and consensus ambiguity thresholds. Rows report the selected resolution for each cancer type under each threshold combination, with group-size and null-model false-discovery criteria held fixed.

Table S14: Implementation, input, optimization and TCGA-use details for the external drug-response baselines.

Method	Architecture and inputs	Model-specific settings	Optimizer and executed epochs	TCGA use
CaDRReS	Inductive expression-to-latent matrix-factorization model using source-cell-line expression and indexed drug identity	Latent dimension, 10	Adam, learning rate 10^{-3}; 7–14 epochs	None
CODE-AE	Shared/private autoencoders with adversarial domain learning and a drug-conditioned regression head	Latent dimension, 128; drug-embedding dimension, 64; reconstruction- and domain-loss weights, 0.1	AdamW, learning rate 10^{-3}, weight decay 10^{-4}; 10–20 epochs	Unlabeled expression from 9,599 TCGA patients was used for domain alignment
GPDRP	GIN/Transformer drug-graph encoder combined with 50 Hallmark pathway-activity features	Hidden dimension, 128; four attention heads	Adam, learning rate 10^{-4}; 7–8 epochs	None
HiDRA	Drug-conditioned gene and pathway-attention model using expression, 50 Hallmark gene sets and 512-bit Morgan fingerprints	Hidden dimension, 128	Adam, learning rate 10^{-3}; 7–8 epochs	None
PANCDR	Expression MLP and three-layer drug GCN with adversarial domain learning	Expression dimension, 256; drug dimension, 128; domain-loss weight, 0.1	AdamW, learning rate 10^{-3}, weight decay 10^{-4}; 9–20 epochs	Unlabeled expression from 9,599 TCGA patients was used for domain alignment

Notes: All baselines were implemented as study-specific continuous-response PyTorch adaptations of their published architectures using a frozen source snapshot. They used the same five immutable ModelID-disjoint partitions, identical held-out test pairs and labels, source-training-fitted expression scaling, and source-training-fitted per-drug response standardization. Hyperparameters were fixed across the five formal splits. Checkpoints were selected by source-validation z-scale mean-squared error under a maximum of 20 epochs, batch size 128 and early-stopping patience of six. Held-out test labels were accessed exclusively for final evaluation. The reported epoch ranges describe the epochs executed across the five formal splits. CODE-AE and PANCDR used TCGA expression without patient drug-response labels.

Alt text: Table S14. Implementation details for CaDRReS, CODE-AE, GPDRP, HiDRA and PANCDR. Columns summarize architectures and inputs, method-specific settings, optimizers, executed epochs and use of unlabeled TCGA expression. The notes describe shared preprocessing, data partitions, checkpoint selection and optimization limits.
